# Mining for Halogenated Metabolites of *Aetokthonos hydrillicola*, the “Eagle Killer” Cyanobacterium

**DOI:** 10.1021/acs.jnatprod.5c00161

**Published:** 2025-05-16

**Authors:** Franziska Schanbacher, Valerie I. C. Rebhahn, Markus Schwark, Steffen Breinlinger, Lenka Štenclová, Kristin Röhrborn, Peter Schmieder, Heike Enke, Susan B. Wilde, Timo H. J. Niedermeyer

**Affiliations:** † Department of Pharmaceutical Biology, Institute of Pharmacy, 9166 Freie Universität Berlin, 14195 Berlin, Germany; ‡ Department of Pharmaceutical Biology/Pharmacognosy, Institute of Pharmacy, Martin-Luther-University Halle-Wittenberg, 06120 Halle (Saale), Germany; § Department of NMR-Supported Structural Biology, 28417 Leibniz-Forschungsinstitut für Molekulare Pharmakologie, 13125 Berlin, Germany; ∥ Simris Biologics GmbH, 12489 Berlin, Germany; ⊥ Warnell School of Forestry and Natural Resources, Fisheries and Wildlife, 1355 University of Georgia, Athens, Georgia 30602, United States

## Abstract

The cyanobacterium Aetokthonos hydrillicola has recently become famous as the “eagle killer”, producing the biindole alkaloid aetokthonotoxin (AETX), a pentabrominated neurotoxin causing the wildlife disease vacuolar myelinopathy. HPLC-HRMS^2^ analysis of extracts from environmental samples of the cyanobacterium revealed the presence of AETX derivatives and biosynthetic intermediates of the cyanobacterial neurotoxin. Mass spectrometry-based molecular networking and other advanced computational data mining techniques were employed to explore the chemical space of natural AETX derivatives. We identified a total of 43 biosynthetic intermediates and derivatives of AETX, including several iodinated derivatives, a rare halogenation in specialized metabolites of freshwater organisms. Structural characterization of these metabolites showed that most of them are AETX derivatives with varying substitution patterns of the bromo or iodo substituents, but also, AETX biosynthetic intermediates and other biindole derivatives were detected. Cytotoxicity assays of two isolated derivatives and AETX showed that they differ markedly in their activity.

## Graphical abstract

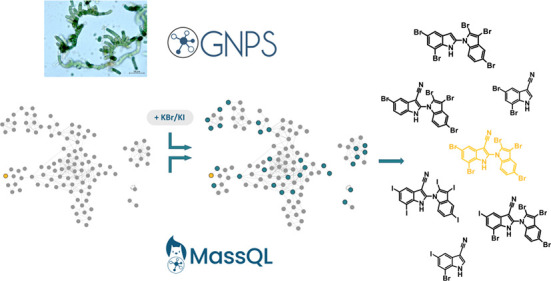

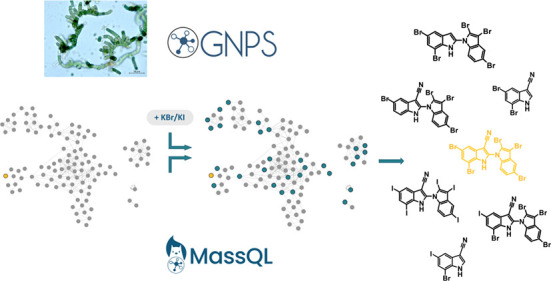

The “eagle killer” cyanobacterium Aetokthonos hydrillicola grows epiphytically on the invasive submerged aquatic plant Hydrilla verticillata in the southeastern United States. It has recently become famous for producing the biindole alkaloid aetokthonotoxin (AETX), a neurotoxin that causes the wildlife disease vacuolar myelinopathy (VM).^1^ From a natural product chemistry point of view, AETX has several unusual features: It is the first described natural 1,2′-bi-1H-indole, and it contains a rare indole-3-carbonitrile substructure and five bromine atoms.

Cyanobacteria are well-known to produce halogenated specialized metabolites (HSMs). The latest version of the cyanobacteria natural product database CyanoMetDB3.0 contains 3087 entries, 21% (641) of which feature halogen atoms.^2,3^ A thorough evaluation revealed that 14% (88) of the HSMs are exclusively brominated and only a small percentage of them contain iodine (0.2%). Eight compounds are both brominated and chlorinated, four are brominated and iodinated, and one is exclusively iodinated. However, all except one of these mixed HSMs contain only a single iodine atom in their scaffold. Most of the brominated compounds (86%) and all of the iodinated compounds originate from marine environments. Therefore, the occurrence of the highly brominated AETX in the freshwater cyanobacterium A. hydrillicola is rather unusual. Besides AETX, the only other polybrominated metabolites known from cyanobacteria are biindoles from Rivularia firma^4^ as well as (poly)­aryl ethers from Leptolyngbya crossbyana (crossbyanols),^5^ from symbiotic cyanobacteria of the sponge Dysidea herbacea,^6–8^ and from Salileptolyngbya sp. (bromoiesols).^9^ This evaluation of cyanobacterial HSMs is consistent with the general observation that bromination is more prevalent than iodination in nature, with iodination being quite unusual in HSMs, although both are much less common than chlorination.^10^

Low amounts of specialized metabolites, their derivatives, or biosynthetic intermediates in the biological starting materials often make these metabolites inaccessible to traditional research methods or resource-intensive and time-consuming to isolate and characterize. Recent methodological developments, particularly in mass spectrometry enhanced by computational approaches, are accelerating the discovery process of previously inaccessible chemical space.^11^ Accurate tandem mass spectrometry raw data are extremely information-rich, capturing unique features of chemical structures or isotope patterns. Comprehensive analysis of such datasets is crucial to gaining a complete understanding of the chemical space of the samples. MassQL is a powerful tool in this regard,^12^ enabling efficient and targeted searches for patterns in complex datasets. Together with Classical Molecular Networking^13^ and Feature-Based Molecular Networking,^14^ tools are now available to propose chemical structures quite fast, even without compound isolation from biomasses, if proper structure elucidation has been performed for a few key compounds beforehand.

As part of our ongoing investigation into the cyanobacterium A. hydrillicola, closer examination of its extracts revealed the presence of putative AETX derivatives.^15^ In this article, we describe the in-depth analysis of A. hydrillicola extracts as well as the isolation, structure elucidation, and cytotoxicity of selected AETX derivatives. Some of the detected derivatives were identified as biosynthetic intermediates, as described by Adak et al. and Li et al.^16–18^ Most interesting, however, was our discovery of iodinated AETX derivatives in Hydrilla verticillata/Aetokthonos hydrillicola samples collected from several water bodies. Supplementation studies confirmed that A. hydrillicola can indeed incorporate iodine at one or more positions in the AETX molecule.

## Results and Discussion

### Detection of Halogenated Metabolites in Aetokthonos hydrillicola Extracts

Our initial analytical screening of environmental samples of Hydrilla/Aetokthonos assemblages collected at J. Strom Thurmond Reservoir, Georgia, USA, indicated the presence of other brominated compounds, most likely derivatives of AETX, as well as biosynthetic precursors.^15^ Moreover, our data revealed the unexpected presence of iodine containing AETX derivatives, suggesting that the halogenases involved in AETX biosynthesis, AetA and AetF, are also capable of accepting iodide as substrate. In the meantime this has been confirmed for AetF by Jiang and Lewis et al.^19,20^ Thus, we collected additional environmental samples from geographically distinct Aetokthonos-positive reservoirs (Long Branch Reservoir, Tussahaw Reservoir, Covington Reservoir, Georgia, USA), in October and November 2021, when, due to its seasonal occurrence, it was likely that AETX was present in the samples.^15,21,22^ Indeed, we confirmed the presence of AETX in these samples. Subsequently, we focused on the characterization of the other brominated and iodinated AETX derivatives in these samples that were obvious from manual inspection of the HPLC-HRMS^2^ data (Figure 1).

**Figure 1:**
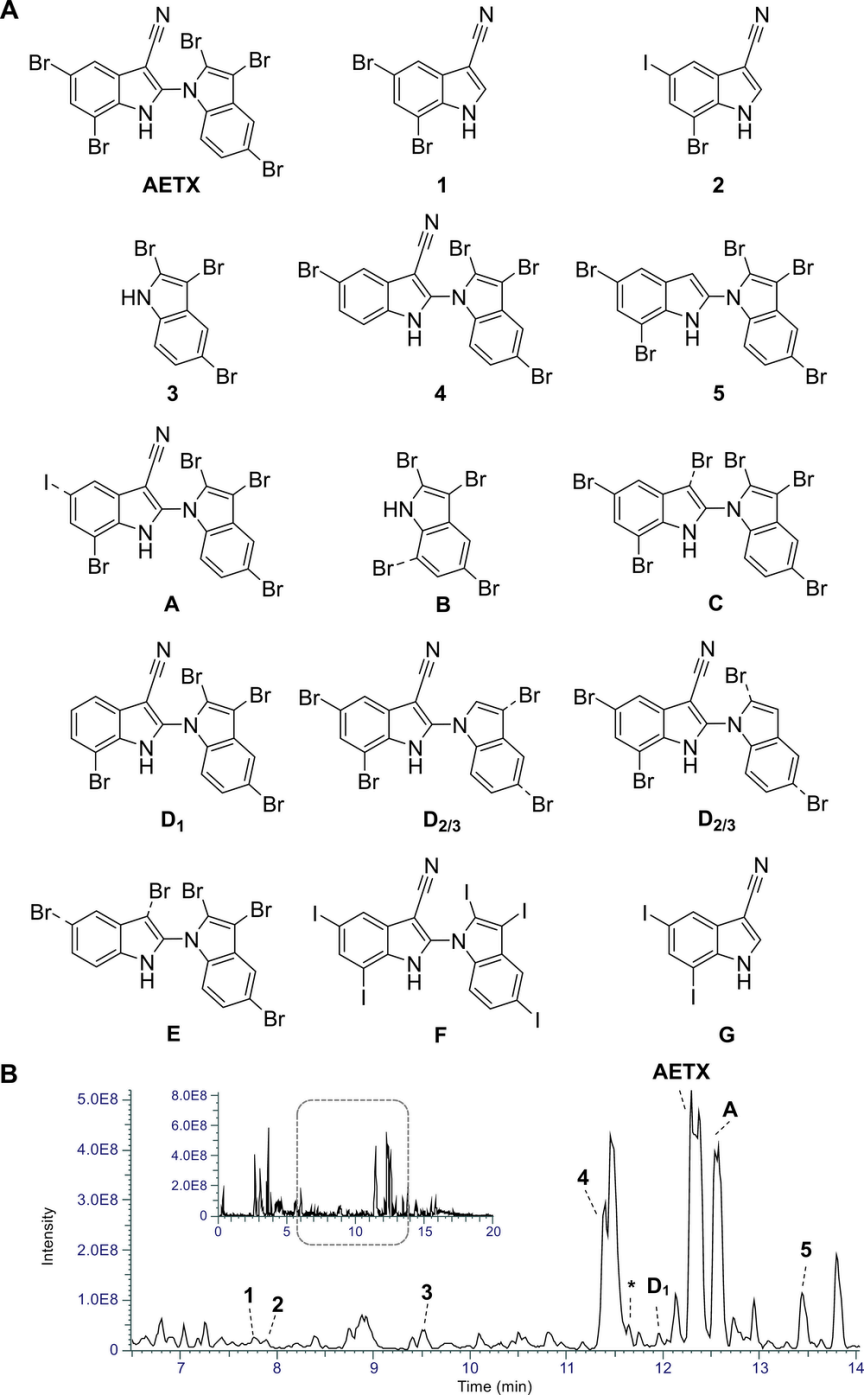
(A) AETX derivatives and biosynthetic intermediates isolated (compounds **1**–**5**) or characterized by HRMS^2^ (postulated structures **A**–**G**; lower confidence level due to missing NMR data). Dashed bonds indicate the most likely position of the respective halogen atom (undeterminable by MS/MS experiments; assignment based on FBMN analysis, annotation of the HRMS^2^ spectra (Figures S5–S27, Tables S4–S12), comparison with **1**–**5**, and prior knowledge about the reactions catalyzed by the AETX biosynthesis enzymes). (B) Base peak chromatograms (overview and expanded view at *t*_R_ = 6.5–14 min, neg. mode) of an extract of an environmental sample of Hydrilla/Aetokthonos collected at Long Branch Reservoir. [The asterisk symbol (*) denotes an uncharacterized brominated/halogenated AETX derivative.]

Among other halogenated metabolites, two molecular ions [M–H]^−^ at *m*/*z* 296.8670 (**1**, calc. molecular formula: C_9_H_4_N_2_Br_2_) and *m*/*z* 344.8533 (**2**, calc. molecular formula: C_9_H_4_N_2_BrI) that eluted earlier than AETX stood out, which we hypothesized correspond to the biosynthetic precursor of the western part of AETX and of the respective iodine-containing derivative (Figure 1, Figure S2). Also, we detected a compound with the molecular ion [M–H]^−^ at *m*/*z* 693.6270 (**A**, calc. molecular formula: C_17_H_6_N_3_Br_4_I), eluting shortly after AETX. The origin of the iodide needed for the biosynthesis of these iodinated derivatives is unknown, as the natural iodide content in freshwater and soil is generally low.^23,24^

As it is known that the biosynthesis of AETX depends on bromide availability,^1^ we supplemented cultures of A. hydrillicola that have not been cultivated in the presence of bromide for at least 5 years with 0.42 mM of potassium bromide (KBr), 0.42 mM of potassium iodide (KI), or a combination of both (0.42 mM each) to induce the biosynthesis of AETX and its derivatives detected in the environmental samples. A comparison of the base peak chromatograms (Figure S1) revealed that four distinct compounds appear to be produced in the presence of iodide only if bromide was absent. HRMS data (Figure S3) supported the hypothesis that these four peaks could be exclusively iodinated derivatives of AETX or biosynthetic intermediates (*m*/*z* 392.8392, C_9_H_3_N_2_I_2_ [M–H]^−^, calc. 392.8391, Δ 0.3 ppm; *m*/*z* 633.7784, C_17_H_7_N_3_I_3_ [M–H]^−^, calc. 633.7780, Δ 0.6 ppm; *m*/*z* 759.6753, C_17_H_6_N_3_I_4_ [M–H]^−^, calc. 759.6746, Δ 0.9 ppm; *m*/*z* 885.5718, C_17_H_5_N_3_I_5_ [M–H]^−^, calc. 885.5712, Δ 0.7 ppm). In the case of an exclusive KI supplementation, AETX derivatives and biosynthetic intermediates with an iodination in more than one position were detected (see Figures 2 and S3), suggesting that both halogenases AetA and AetF, which brominate tryptophane during AETX biosynthesis, can accept iodide as a substrate. In the case of both KBr and KI supplementation, iodinated derivatives with only one specific iodination position were formed and produced in lower abundance compared to the corresponding brominated derivatives (Figure S2), suggesting that the halogenases strongly prefer bromide over iodide. The natural occurrence of cyanobacterial iodinated specialized metabolites is rare, and there are no known iodinated compounds from freshwater cyanobacteria, yet. The known compounds comprise 3,6-diiodocarbazole, isolated from the cyanobacterium Kyrtuthrix maculans, along with two brominated carbazoles;^25^ jamaicamide F from Moorena producens,^26^ tasihalide A and B from Symploca sp.,^27^ and bromoiesol B/bromoiesol sulfate B, iodinated in one position, from Salileptolyngbya sp.,^9^ all of marine origin.

**Figure 2:**
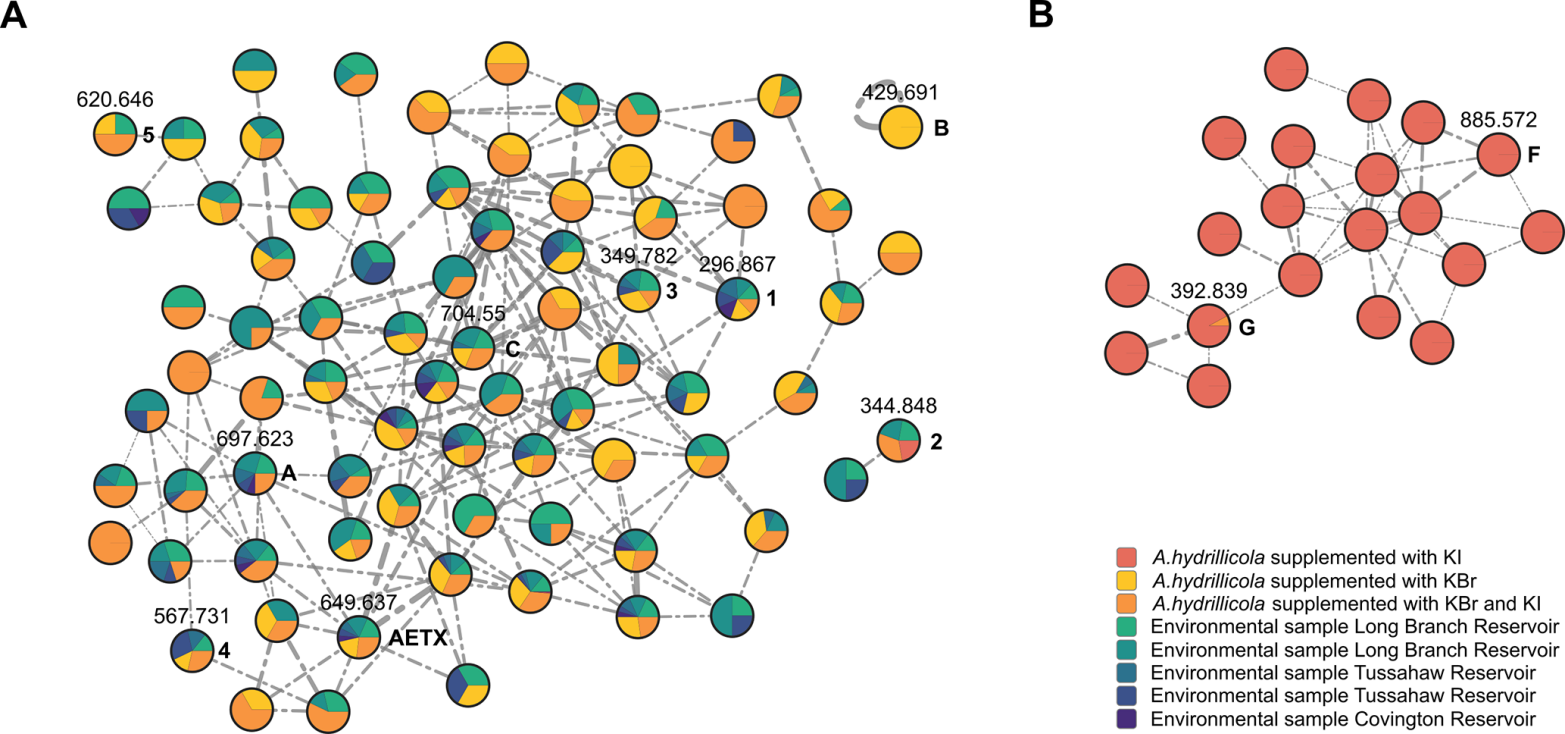
Classical Molecular Networking analysis of the environmental samples and the supplementation experiments. Cluster **A**: compounds detected in environmental samples of Hydrilla/Aetokthonos assemblages collected from different water bodies and at different sampling locations in each case, in KBr-supplemented biomass and in KBr-/KI-supplemented biomass. Cluster **B**: (poly)­iodinated derivatives were produced exclusively in the absence of bromide in KI-supplemented biomass. AETX derivatives first noticed in the environmental samples and two purely iodinated derivatives are highlighted, nodes are connected for cosine scores ≥ 0.7, edge width correlating to cosine score.

Intrigued by these findings, we decided to characterize the halogenated metabolites of A. hydrillicola in detail. First, we used Classical Molecular Networking^13^ to obtain an overview of the chemical space of all produced AETX derivatives. We identified the AETX-based networks by the [M–H]^−^ ion of AETX and the putative penta-iodinated derivative. We observed two distinct main clusters, differing according to the origin of the samples: The purely iodinated derivatives occurred exclusively in the KI supplemented samples but not in the environmental samples (Figure 2). In its cluster, AETX was surrounded by a series of other compounds that were found to be both purely brominated derivatives but also derivatives that originated from the combined supplementation. These compounds probably contain both halogens. The environmental samples included both variants (Figure 2). The previously mentioned nodes for the [M–H]^−^ ions *m*/*z* 296.8670 (**1**) and *m*/*z* 693.6270 (**A**) were also found in the cluster surrounding AETX, supporting the previous hypothesis about the structural relationship between these compounds. The same holds true for the later isolated compounds **3**, **4**, and **5**. The interpretation of the clusters was complicated by the fact that some individual compounds were represented by more than one node: ions of the same compound but with different isotopic compositions in significant intensities, such as in brominated compounds, result in separate nodes in the molecular network. Therefore, every brominated AETX derivative can cause several nodes. Care had to be taken when interpreting the data to assign the appropriate retention times. This became important again later when evaluating the results of the MassQL query and Feature Based Molecular Networking.

Upon closer examination of the high-resolution tandem mass spectra of the putative AETX derivatives, we observed that both bromide (*m*/*z* = 78.9183 and 80.9162) and iodide fragment ions (*m*/*z* = 126.9045) were always present for Br- or I-containing AETX derivatives, respectively. This prompted us to use MassQL queries (Figure S4) to mine the datasets for bromine- and iodine-containing compounds, which should reveal all brominated and iodinated derivatives as well as biosynthetic intermediates of AETX. The MassQL query identified a total of 43 compounds, of which 15 were brominated (Table S1), 11 were iodinated (Table S2), and 17 were identified as mixed brominated and iodinated derivatives (Table S3). AETX was also among the query results. The sum formulas calculated from the HRMS data suggested that all query results were either AETX derivatives or brominated/iodinated indole or indole nitrile variants (Tables S1–S3). Further analysis based on isolation and NMR structural elucidation, evaluation of the MS/MS spectra, and an in-depth Feature Based Molecular Networking analysis^14^ confirmed this assumption. First of all, we could detect both the western and the eastern indoles of the biindole AETX as individual compounds, again with changing extents of bromination and/or iodination. Our analysis revealed the presence of dibrominated, tribrominated, and even tetrabrominated eastern indoles in the dataset with the dibrominated indole present in three isomers. Interestingly, while diiodinated and triiodinated indoles were also identified, no tetraiodinated indole derivative was detected. The western indole nitrile moiety was found as both dibrominated and diiodinated derivatives. Additionally, two monoiodinated variants of the indole nitrile were also detected, indicating iodination at both C5 and C7. All biosynthetic intermediates postulated by Adak et al. were found, except for 5-bromo-tryptophan and 5,7-dibromo-tryptophan, which confirms the AETX biosynthetic pathway (Scheme 1).^17^ However, our results show that, in vivo, the biosynthesis enzymes have a higher degree of flexibility: The presence of tetrabrominated indole (**B**), as well as the presence of desnitrile-AETX (**5**), indicate that the tryptophanase AetE can also use 5,7-dibromotryptophan for transformation into an indole, and that the indole-coupling oxidase AetB is able to couple 2,3,5-tribromoindole and 5,7-dibromoindole to desnitrile-AETX (**3**). Moreover, AetD and AetA also have higher flexibility than described,^17^ as we also found AETX derivatives lacking one bromine atom (**4**) or carrying one additional bromine atom in the western indole (**C**). In addition, both monobromoindole-3-carbonitriles, brominated at C5 or C7, were detected. Interestingly, the ion intensity of one compound was markedly higher than that of the other, indicating that bromination at a particular position may occur earlier or with a higher probability than that at the other position, ultimately leading to the formation of dibromotryptophan. Finally, the flexibility of the halogenases is clearly demonstrated by the occurrence of a variety of different substitution patterns and the acceptance of iodide as a substrate in the case of both AetF and AetA. The capacity of AetF to halogenate using bromine or iodine as substrates has already been demonstrated, but it has not yet been reported for AetA.^28^ Chloride is barely accepted by the Aetokthonos halogenases, as only minute amounts of a chlorinated AETX derivative could be detected at *m*/*z* 601.6914 ([M–H]^−^, C_17_H_5_N_2_Br_4_Cl, calc. 601.6914, Δ 0.5 ppm). In mixed KBr/KI supplementation, iodination at C5 is highly preferred over the other positions, resulting in **2** and **A**. In the absence of bromide, this preference does not exist, as indicated by the polyiodinated derivatives. The differing ion intensities detected for the AETX derivatives lacking one bromine atom, **4** being the main isomer, suggest that the halogenase AetF frequently skips its second halogenation step at C7, or that AetD can convert monobrominated tryptophane to monobromoindolenitrile before AetF can perform the second halogenation (Scheme 1). The flexibility of cyanobacterial FAD-dependent halogenases to use different halogens as substrates was also shown by the replacement of chlorine with bromine in carbamidocyclophanes^29^ and with bromine and iodine in cryptophycin-1 ^30^ when the producer strains were supplemented with the respective halide ions.

**Scheme 1:**
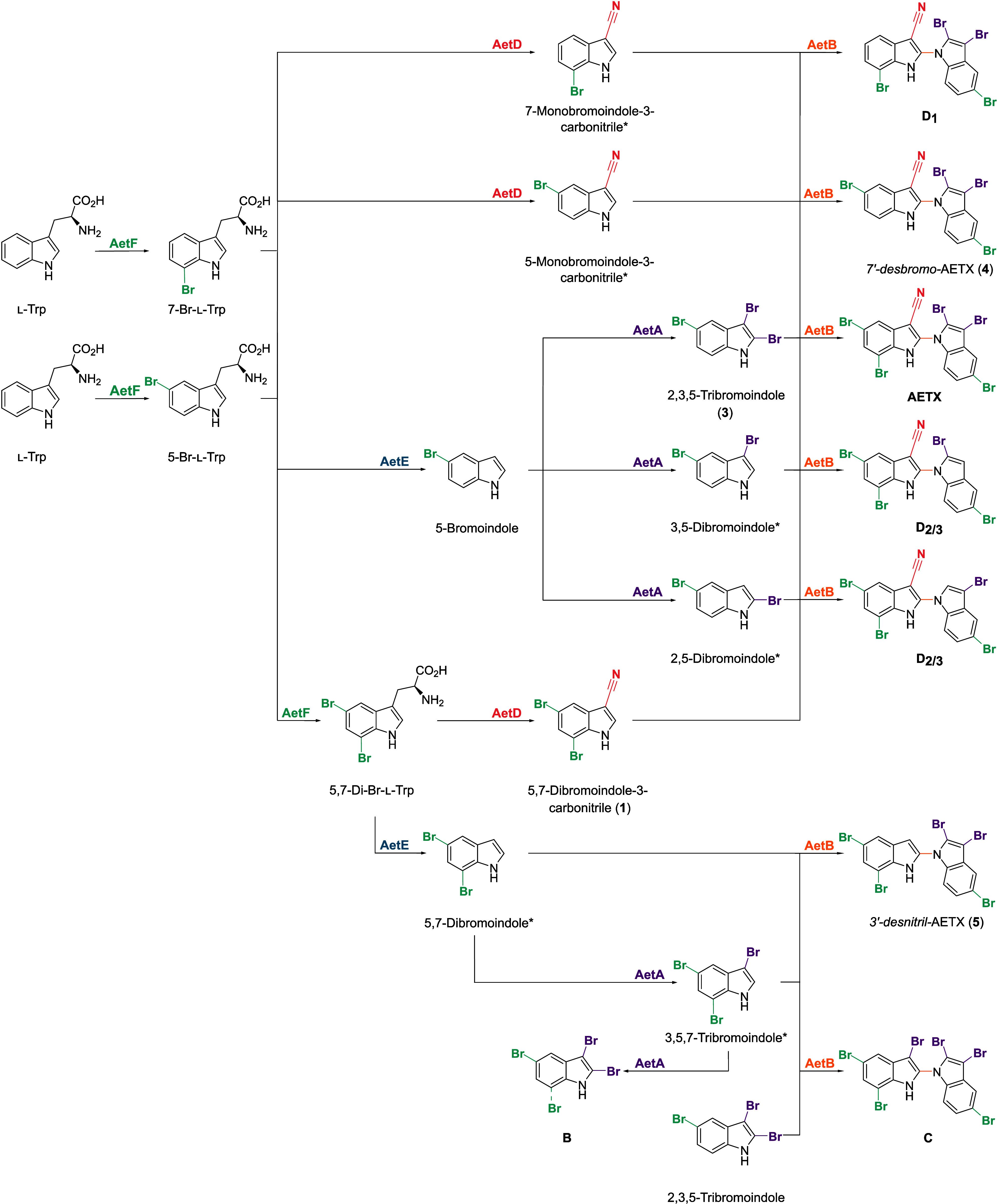
Postulated Biosynthesis of **3**, **4**, **B**, **C**, and **D**_**1**_, and **D**_**2/3**_, Based on the Work of Adak et al.^17^ and This Study, Demonstrating the Flexibility of the Biosynthesis Enzymes in Vivo

a * indicates compounds that were detected by HPLC-MS/MS but not isolated.

Finally, the MassQL query and the HRMS analysis also revealed two [M–H]^−^ ions at *m*/*z* 314.8779 (C_9_H_5_ON_2_Br_2_, calc. 314.8774, Δ 1.6 ppm) as well as at *m*/*z* 410.8502 (C_9_H_5_ON_2_I_2_, calc. 410.8502, Δ 1.2 ppm), indicating a biosynthetic intermediate of the nitrile formation, as postulated by Li et al. and Adak et al.^16,18^

### Isolation and Structure Elucidation

To unequivocally prove the structure of at least some of the AETX derivatives and biosynthesis precursors discussed above by NMR spectroscopy, A. hydrillicola was cultured on a larger scale supplemented with 0.42 mM KBr and KI for compound isolation.

Compounds **1** and **2** differ in the exchange of a bromine substituent at one position for iodine. Due to their similar retention behavior, separating these two compounds was challenging. Even under near-isocratic conditions and by assessing different stationary phase modifications, no baseline separation could be achieved. While **1** could be isolated in good purity, **2** still contained a residual amount of **1**. Nevertheless, the NMR data obtained for **2** allowed the assignment of which bromine was substituted for an iodine. Compound **1** was isolated as a white, amorphous powder. HRMS analysis resulted in a [M–H]^−^ ion at *m*/*z* 296.8670 (C_9_H_3_N_2_Br_2_, calc. 296.8668, Δ 0.7 ppm). The isotope pattern confirmed the presence of two bromine substituents in the molecule. Based on the molecular formula, we hypothesized that **1** was the western indole nitrile moiety of AETX. The ^1^H NMR spectrum of **1** (Table 1) showed, as expected, two doublets at δ_H_ 7.71 and δ_H_ 7.83 ppm (*J* = 1.7 Hz, respectively), consistent with meta-coupled aromatic protons. A third signal appeared at δ_H_ 8.40 ppm (*J* = 3.1 Hz) coupled to the broad singlet at δ_H_ 12.73 ppm assigned to the strongly deshielded NH proton in the COSY spectrum. The ^13^C NMR spectrum corroborated the indole nitrile framework, as previously described for AETX (Table 2). The characteristic signal at δ_C_ 89.3 ppm (C3′) indicated the quaternary carbon bearing the electron-withdrawing nitrile group. Additionally, the signals at δ_C_ 117.7 ppm (C5′) and δ_C_ 109.8 ppm (C7′) were consistent with the presence of bromine at these positions. Key ^1^H–^13^C-HMBC correlations further supported the structure, with the C4′ proton exhibiting a cross-peak with C3′ (δ_C_ 89.3 ppm), and an additional correlation between the C4’ proton and C5′ (δ_C_ 117.7 ppm), confirming the bromine substitution at C5′. Furthermore, the C6′ proton showed HMBC correlations with both C5′ and C7′ (δ_C_ 109.8 ppm), providing strong evidence for the second bromination at C7′. The full assignment was unambiguously supported by HSQC, and HMBC experiments (Figure 3, Table 1, and Figures S32–S34), which also allowed assignment of the remaining signals.

**Figure 3:**
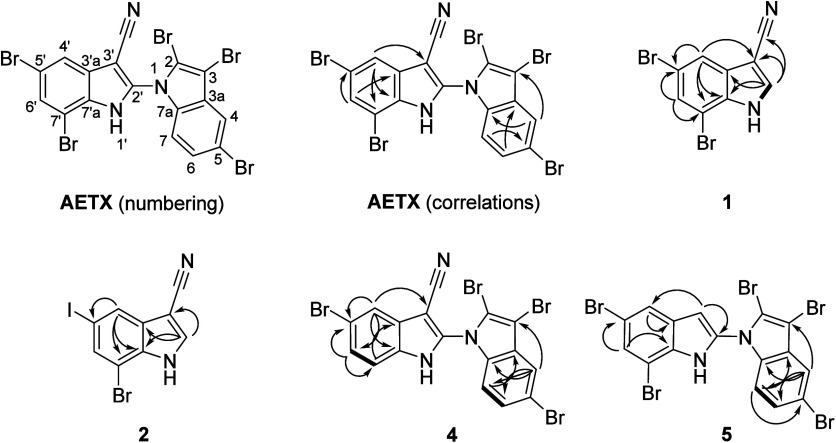
Systematic numbering of AETX and main 2D NMR correlations of **1**, **2**, **4**, and **5**. COSY correlations are shown with bold bonds and HMBC correlations with arrows.

**Table 1:** ^1^H (600 MHz) and ^13^C (150 MHz) NMR Spectroscopic Data and HMBC Correlations of **1** and **2** in DMSO-*d*_6_

	**1**			**2**		
pos.	δ_C_, type	δ_H_ (J in Hz)	HMBC	δ_C_, type	δ_H_ (J in Hz)	HMBC
1′		12.73, s			12.73, s	
2′	140.5, CH	8.4, d (3.1)	**3′**, **3a′**, **4′**, **7a′**, **8′**	139.9, CH	8.35, d (3.1)	**3′**, **3a′**, **7a′**
3′	89.3, C			88.3, C		
3a′	131.2, C			133.3, C		
4′	123.8, CH	7.83, d (1.7)	**3′**, **3a′**, **5′**, **7′**, **7a′**	136.3, CH	7.82, d (1.8)	**5′**, **6′**, **7′**, **7a′**
5′	117.7, C			89.1, C		
6′	131.2, CH	7.71, d (1.7)	**4′**, **5′**, **7′**, **7a′**	129.7, CH	7.98, d (1.8)	**7a′**
7′	109.8, C			109.8, C		
7a′	136.4, C			136.5, C		
8′	118.3, C					

**Table 2:** ^1^H (600 MHz) and ^13^C (150 MHz) NMR Spectroscopic Data and HMBC Correlations of AETX,^1^ **4**, and **5** in DMSO-*d*_6_

	AETX		**4**			**5**		
position	δ_C_, type	δ_H_ (J in Hz)	δ_C_, type	δ_H_ (J in Hz)	HMBC	δ_C_, type	δ_H_ (J in Hz)	HMBC
2	117.2, C		117.2, C			117.8, C		
3	98.4, C		92.9, C			94.4, C		
3a	128.4, C		127.7, C			128.1, C		
4	120.9, CH	7.76, d (1.91)	120.0, CH	7.63, d (1.9)	3, 6, 7, 7a	120.6, CH	7.71, d (2.0)	3, 6, 7, 7a
5	115.6, C		115.3, C			114.9, C		
6	127.7, CH	7.50, dd (8.77, 1.91)	126.1, CH	7.34, dd (8.8, 1.9)	4, 7, 7a	127.1, CH	7.41, dd (8.7, 2.0)	4, 7, 7a
7	113.5, CH	7.36, d (8.77)	114.0, CH	7.13, d (8.8)	3a, 4, 7a	113.4, CH	7.07, d (8.7)	3a, 4, 5, 7a
7a	136.3, C		136.0, C			136.1, C		
1′		13.76, br s		8.33, br s			8.39, br s	
2′	137.7, C		146.5, C			140.6, C		
3′	85.2, C					129,2, CH	7.59, d (7.0)	2′, 4′, 7a′
3a′	131.5, C		133.3, C			132.3, C		
4′	121.1 CH	8.04, s	118.7, CH	7.52, d (2.0)	5′, 6′, 7a′	126.4, CH	7.78, d (7.0)	3a′
5′	115.3, C		111.4, C			112.8, C		
6′	129.7, CH	7.90, s	121.4, CH	7.04, dd (8.5, 2.0)	4′, 5′, 7a′	115.4, CH	7.48, s	5′, 7a′
7′	107.0, C		120.5, CH	7.39, d (8.5)	3a’, 4’, 5′			
7a′	127.9, C		142.8, C			127.6, C		
8′	112.7, C		106.5, C					

Compound **2** was isolated as a white amorphous powder. HRMS analysis resulted in a [M–H]^−^ ion at *m*/*z* 344.8533 (C_9_H_3_N_2_BrI, calc. 344.8530, Δ 0.9 ppm). The presence of one bromine substituent in the molecule was evident from the isotope pattern, while the presence of one iodine substituent was obvious from the fragment ion corresponding to iodine in the HRMS^2^ spectrum. Comparing the ^1^H–^13^C-HMBC spectra of **2** and **1** (Table 1) showed that **1** contained bromine at C5′, whereas **2** featured iodine at the same position. This substitution was supported by the following observations: In the ^1^H–^13^C-HMBC spectrum of **2**, a different HMBC correlation was observed between the C4′ proton and a carbon with a chemical shift of δ_C_ 89.1 ppm. This correlation differed from that in **1**, where the proton at C4′ correlated with the carbon at C3′ with the slightly different chemical shift of δ_C_ 89.3 ppm. Additionally, **2** lacked the HMBC correlation observed in **1** between the proton at C4′ and the carbon at δ_C_ 117.7 ppm (corresponding to C5′ in **1**, which featured the bromine). The distinct signal at δ_C_ 89.1 ppm, along with the absence of the HMBC correlation between the proton at C4′ and the carbon at δ_C_ 117.7 ppm, strongly supported the iodination of C5′ in **2**. The signals of C3′ in **1** and C5′ in **2** exhibited very similar chemical shifts, with δ_C_ 89.3 ppm in **1** and δ_C_ 89.1 ppm in **2**, which might suggest a potential wrong assignment. However, the signal at δ_C_ 88.3 ppm in **2** is unmistakably assigned to C3′ in **2**, as confirmed by the HMBC correlation from the proton at C2′ to this carbon. This HMBC correlation helps to resolve the potential ambiguity and distinguishes C3′ in **2** from C5′ in **2**, despite the close chemical shift of C5′ (δ_C_ 89.1 ppm) in 2 and C3′ (δ_C_ 89.3 ppm) in **1**. The other HMBC correlations involving the C4′ proton, such as those to C7′ and C7a′, remained unchanged between **2** and **1**, providing additional confirmation of the iodine substitution at C5′ in **2**.

Compound **3** was isolated as a white amorphous powder. HRMS analysis resulted in a [M–H]^−^ ion at *m*/*z* 349.7821 (C_8_H_3_NBr_3_, calc. 349.7821), which also appears as a key ion in the HRMS^2^ data of AETX^1^ and further AETX derivatives studied in this work. The presence of three bromine substituents in the molecule is evident from the isotope pattern. The molecular formula indicates that **3** is the eastern indole moiety of AETX. The ^1^H NMR spectrum indeed showed the expected three proton signals in the aromatic region. An additional broad singlet at δ_H_ 8.39 ppm, which was assigned to the NH group, was also detected. The presence of two doublets at δ_H_ 7.43 ppm (*J* = 2.0 Hz) and δ_H_ 7.33 ppm (*J* = 8.7 Hz), along with a doublet of doublets at δ_H_ 7.27 ppm (*J* = 8.7 and *J* = 2.0 Hz), indicated the presence of two directly adjacent protons and a third proton in the meta or para position. This observations confirmed our assumption that **3** is the eastern indole of AETX.

Compound **4** was isolated as a white, amorphous powder. HRMS analysis resulted in a [M–H]^−^ ion at *m*/*z* 567.7299 (C_17_H_7_N_3_Br_4_, calc. 567.7301, Δ 0.4 ppm). The presence of four bromine atoms, one less than AETX, was also evident from the isotope pattern. The ^1^H NMR spectrum showed six proton signals in the aromatic region that were part of two spin systems (Table 2). The ^1^H and ^13^C chemical shifts of one of the indoles were virtually identical to those of the eastern part of AETX,^1^ including the characteristic ortho and meta couplings (*J* = 8.8 and 1.9 Hz), indicating that one bromine atom was missing at the western part of AETX. In contrast to AETX, an additional proton directly adjacent to H-6′ but not to H-4′ was observed in the western part of this AETX derivative. Two doublets at δ_H_ 7.52 ppm (*J* = 2.0 Hz) and 7.39 ppm (*J* = 8.5 Hz), and a doublet of doublets at δ_H_ 7.27 ppm (*J* = 8.5 and 2.0 Hz), clearly indicate two directly neighboring protons and another proton in meta position—identical to the eastern indole of **1**. The substitution pattern of the western indole was also evident from the HMBC data (Figure 3, Figure S50), confirming **4** to be the tetrabrominated 7′-desbromo-AETX.

Compound **5** was isolated as a white, amorphous powder. HRMS analysis resulted in a [M–H]^−^ ion at *m*/*z* 620.6454 (C_16_H_6_N_2_Br_5_, calc. 620.6453, Δ 0.2 ppm). The isotopic pattern clearly indicated the presence of five bromine substituents. Based on the determined molecular formula, we hypothesized that **5** is the desnitrile derivative of AETX. The ^1^H NMR spectrum exhibited six aromatic proton signals (Table 2), three of which again were readily assigned to the eastern indole (see discussion for **4**). Two doublets appeared at δ_H_ 7.59 and δ_H_ 7.78 ppm (*J* = 7 Hz), alongside a singlet at δ_H_ 7.48 ppm. The doublet at δ_H_ 7.59 ppm was assigned to the proton at position C3′, while the doublet at δ_H_ 7.78 ppm corresponded to the proton at position C4′. This assignment was unequivocally confirmed by HMBC cross-peaks, as the proton signal at δ_H_ 7.59 ppm showed clear correlations with the carbons at C2′ and C4′. The remaining singlet at δ_H_ 7.48 ppm, corresponding to the proton at C6′, exhibited an HMBC cross-peak with the carbon at C5′, further supporting its assignment and demonstrating good agreement with the corresponding proton signal in AETX. The NMR data thus support our assumption that **5** is desnitrile-AETX. The presence of **5** in the extract support the assumption that AetE is also capable of converting 5,7-dibromotryptophan to a 5,7-dibromoindole, as discussed above (Scheme 1).

In addition, a sixth compound (**C**) was isolated. Attempts to acquire 1D and 2D NMR spectra were unsuccessful due to insufficient sample quantity. However, structural characterization was performed using high-resolution tandem mass spectrometry experiments. HRMS analysis resulted in a [M–H]^−^ ion at *m*/*z* 698.5561 (C_16_H_5_N_2_Br_6_, calc. 698.5558, Δ 1.9 ppm). HRMS^2^ data and a biosynthesis proposal suggest the presence of three bromine atoms in both the western and eastern moieties (see Scheme 1, as well as Figures S11, S12, and Table S6).

### In-Depth Feature Based Molecular Networking Analysis of AETX Derivatives

Based on the key compounds **1**–**5** confirmed by NMR data, the postulated biosynthesis of AETX,^17^ and the available HRMS^2^ data, we carried out an in-depth FBMN analysis to propose the structures of as many as possible of the AETX derivatives detectable in the data (Figure S25). First, we found three isomers of **4**. Only in two of them, **4** and **D**_**1**_(*m*/*z* 567.7302, C_16_H_7_N_3_Br_4_ [M–H]^−^, calc. 567.7301, Δ 0.2 ppm, Table S1), a fragment corresponding to the eastern subunit (*m*/*z* 351.7801, Table S4) could be observed, revealing that **K** has to be 5′-desbromo-AETX. For the other two isomers, **D**_**2**_ (*m*/*z* 567.7302, C_16_H_7_N_3_Br_4_ [M–H]^−^, calc. 567.7301, Δ 0.2 ppm, Table S1), and **D**_**3**_ (*m*/*z* 567.7308, C_16_H_7_N_3_Br_4_ [M–H]^−^, calc. 567.7301, Δ 1.2 ppm, Table S1), this characteristic fragment was not observed, suggesting that they are 2-desbromo-AETX and 3-desbromo-AETX. In addition to **5**, an additional desnitrile-AETX isomer, **E** (*m*/*z* 620.6456, C_16_H_7_N_2_Br_5_ [M–H]^−^, calc. 620.6453, Δ 0.5 ppm, Table S1), was found in the FBMN close to hexabromo-AETX. Again, a fragment corresponding to the eastern subunit (*m*/*z* 351.7801; see Table S5, Figure S10) was observed, suggesting that the fifth bromine atom is located at the 3′ position. The HRMS^2^ data do not allow us to determine definitively whether the second bromine atom of the eastern moiety is in the C5′ or C7′ position. However, the pentabrominated derivatives suggest that both variants are plausible.

AETX derivatives containing more than two iodine atoms were detected only in the KI supplementation. Beside a pentaiodo-AETX (**F**, *m*/*z* 885.5718, C_17_H_5_N_3_I_5_ [M–H]^−^, calc. 885.5712, Δ 0.7 ppm; see Table S7, Figure S13), two tetraiodo-AETX isomers could be observed. For the pentaiodo-AETX, the tetraiodo iomers, and the two triiodo derivatives, the HRMS^2^ data do not provide any information on the distribution of the substituents on the western or eastern indoles. Almost exclusively, fragments that have lost up to five iodo substituents (Table S7) can be observed. The proposed structures for the tetraiodinated compounds are based on the data for the tetrabrominated structural variants, in which the two structures with a substituent at the 5′ or 7’ position were detected with a higher abundance in the extracts. It was not possible to propose structures for the triiodinated derivatives based on tandem-MS data. All in all, the flexibility of FAD-dependent halogenases to utilize diverse halogens as substrates, coupled with the flexibility afforded by their position within the scaffold, was demonstrated. This finding becomes even more evident upon examination of the mixed halogenated AETX derivatives (Table S3). However, the strongly differing ion intensities of the mixed halogenated AETX derivatives and their respective isomers indicate that only one isomer with a particular substitution pattern is preferentially formed. Moreover, only one specific isomer is produced for all conceivable substitution patterns if the derivative contains only one iodine atom. Our data suggest that (i) the halogenase AetF is more prone to do an iodination in one position, preferring the C5′ position, and (ii) both halogenases seem to prefer bromine over iodine when both anions are available.

### Bioactivity Characterization

As AETX was found to be moderately cytotoxic,^31^ AETX and its derivatives **1**, **3**–**5** were tested on HCT116 colon carcinoma cells in vitro using the sulforhodamin B (SRB) assay (Table 3).^32^ Due to the low amounts that have been isolated, we decided to test 0.1, 1, and 10 μM each, based on previous bioactivity assays that found the EC_50_ of AETX to be 5 μM.^31^ This three-point assay allows for at least a qualitative comparison of the cytotoxicity of the compounds. At the lowest concentration tested (0.1 μM), no cytotoxicity was observed for any of the derivatives. At 1 μM, cells treated with AETX, **1**, and **4** showed reduced cell viability in terms of reduced SRB absorbance when compared to the solvent control. At 10 μM, **5** showed slightly reduced cell viability, as well, while the effect of AETX, **1** and **4** was increased. Interestingly, at this concentration, **1** had a significantly stronger impact on HCT116 cell viability than AETX, while the effect of **4** was comparable to that of AETX. **3** showed no effect on cell viability at any concentration tested. In summary, these results indicate that the presence of the nitrile group is important for toxicity and that interestingly the presence of the eastern indole in AETX might decrease cytotoxicity in the used assay.

**Table 3:** Sulforhodamine B Cytotoxicity Assay^a^

	0.1 μM		1 μM		10 μM	
treatment	mean [%]	±SD [%]	mean [%]	±SD [%]	mean [%]	±SD [%]
AETX	100	11	70	11	53	16
**1**	103	13	73	14	30	6
**3**	102	7	102	7	84	11
**4**	98	6	66	7	49	10
**5**	102	13	102	10	97	12

a Assay performed in three independent biological replicates. Mean absorbance values were normalized to the solvent control (0.1% DMSO). SD = standard deviation.

## Experimental Section

### General Experimental Procedures

NMR spectra were recorded in DMSO-*d*_6_ on a Bruker Model Avance III (Bruker BioSpin GmbH) equipped with a QCI cryoprobe with one axis self-shielding gradient, operating at 600 MHz (^1^H) or 150 MHz (^13^C) at 300 K. Chemical shifts are reported in ppm, spectra were calibrated related to solvent’s residual proton chemical shift (δ_H_ 2.50, δ_C_ 39.5). NMR data were analyzed with MestReNova (version 14.3.0–30573, Mestrelab Research S.L.) after processing using Auto Phase Correction and Auto Baseline Correction. HPLC separations were performed with an Agilent Model 1260 Infinity II instrument with a diode array detector (Model 1260 DAD). LC-MS/MS data were acquired as described below.

### Cyanobacterium, Media, Growth Conditions, and Environmental Samples

For the supplementation studies, 200 mL small-scale cultures of A. hydrillicola were cultivated in BG-11 medium without supplementation, or supplemented with either 0.42 mM KBr (≥99%, VWR International, Belgium), 0.42 mM KI (≥99%, Carl Roth, Germany) or 0.42 mM KBr and KI, at 21 °C, under continuous illumination with Osram LUMILUX fluorescent lamps (50 μmol photons m^–2^ s^–1^), aerated with 5% CO_2_ in sterile filtered air in 250 mL Schott Duran bottles. The large-scale culture of A. hydrillicola was cultivated in BG-11 medium, supplemented with 0.42 mM KBr and KI, at 25 °C, under continuous illumination with Sylvania GROLUX fluorescent lamps (50–200 μmol photons m^–2^ s^–1^), and aerated with 0.5–5% CO_2_ in sterile filtrated air in 20 L polycarbonate carboys. To minimize cell death and lysis, cultures were harvested biweekly using a 100 μm mesh plankton net and diluted with fresh medium (semicontinuous cultivation to avoid entering stationary phase). For both the small-scale and large-scale cultures, the biomass was subsequently lyophilized and stored at room temperature until further processing. The environmental samples were collected from geographically distinct Aetokthonos-positive reservoirs (Long Branch Reservoir, Tussahaw Reservoir, Covington Reservoir, Covington, GA, USA) in October and November of 2021.^22^

### Extraction and Isolation of Aetokthonotoxin Derivatives

For HPLC-MS analysis, freeze-dried biomass and the environmental samples were suspended in 50% MeOH (v/v) at a solvent-to-biomass ratio of 1 mL/12.5 mg dry biomass, homogenized by vortexing, treated with an ultrasonication rod (Bandelin, 30 s, amplitude 100%, no pulsation), and again vortexed for 90 s. After centrifugation (4700 rpm, 10 min), the supernatants were collected, and the biomass pellets were extracted again with 50% MeOH (v/v) and twice with 80% MeOH (v/v) in the same manner. The combined supernatants were dried in a vacuum centrifuge and reconstituted with MeOH 80% (v/v, 10 mg/mL) for HPLC-MS analysis. For compound isolation, a total of 52.0 g of dry biomass from large-scale cultures was suspended in 50% MeOH (v/v) at a solvent-to-biomass ratio of 16 mL/g, homogenized by vortexing, treated with an ultrasonication rod (Bandelin, UW 2070, 2 × 60 s, amplitude 100%, no pulsation), and extracted on an overhead shaker for 20 min. After centrifugation (20 min, RT, 10.000 rpm), further extraction was performed in the manner described above using 50%/80% MeOH (v/v). The supernatants were combined and filtered (Rotilabo Faltenfilter Type 600P) and dried in vacuo using a vacuum centrifuge, resulting in 8.5 g of extract. Divided into portions of 1.5 g each, the extract was prepared as a dry load (support material: Celite) and fractionated using flash chromatography on a C_18_ cartridge (CHROMABOND Flash RS 80 C_18_ec, 15–40 μm, 30.9 × 249 mm, Macherey Nagel) on a preparative HPLC system. A binary gradient from 30% to 60% MeOH in water in 6 min, 60%–100% for further 18 min and 100% MeOH for 10 min was used. In total, 18 fractions were collected. The fractions **F6**–**F15** containing putative AETX derivatives and biosynthetic derivatives were dried in vacuo. Fractions **F7** and **F8** were combined, dissolved in 2 mL of MeCN 80% (v/v), and subjected to semipreparative HPLC using a Luna PFP2 column (250 × 10 mm, 5 μm, 100 Å, Phenomenex) and a gradient from 55 to 80% MeCN in water (0.1% formic acid each) for 16 min at 5 mL/min, resulting in **1** (0.7 mg, t_R_ 7.3 min), **2** (0.45 mg, *t*_R_ 7.5 min). The following chromatographic parameters were used to isolate **3** (1.1 mg, *t*_R_ 9.5 min), **4** (0.56 mg, *t*_R_ 12.8 min), and **5** (0.9 mg, *t*_R_ 12.3 min) from **F10**, **F14** and **F15**: Luna PFP2 column (250 × 10 mm, 5 μm, 100 Å, Phenomenex), binary gradient from 64% to 98% MeCN in water (0.1% formic acid each) at 5 mL/min in 17 min.

#### 5,7-Dibromo-1H-indole-3-carbonitril (**1**)

White amorphous powder, UV (MeCN) λ_max_ 223 nm, 280 nm; ^1^H and ^13^C NMR (see Table 1); HRESIMS *m*/*z* 296.8670 [M–H]^−^ (C_9_H_3_Br_2_N_2_ calc. 296.8668, Δ 0.7 ppm).

#### 5-Iodo-7-bromo-1H-indole-3-carbonitril (**2**)

White amorphous powder, UV (MeCN) λ_max_ 230 nm, 280 nm; ^1^H and ^13^C NMR (see Table 1); HRESIMS *m*/*z* 344.8533 [M–H]^−^ (C_9_H_3_BrIN_2_ calc. 344.8530, Δ 0.9 ppm).

#### 2,3,5-Tribromo-1H-indole (**3**)

White amorphous powder, UV (MeCN) λ_max_ 227 nm, 291 nm; ^1^H NMR see Figure S53; HRESIMS *m*/*z* 349.7821 [M–H]^−^ (C_8_H_3_Br_3_N calc. 349.7821, Δ 0 ppm).

#### 7′-Desbromo-AETX (**4**)

White amorphous powder, UV (MeCN) λ_max_ 223 nm, 291 nm; ^1^H and ^13^C NMR (see Table 1); HRESIMS *m*/*z* 567.7299 [M–H]^−^ (C_17_H_6_Br_4_N_3_ calc. 567.7301, Δ 0.4 ppm).

#### 3′-Desnitril-AETX (**5**)

White amorphous powder, UV (MeCN) λ_max_ 229 nm, 291 nm; ^1^H and ^13^C NMR (see Table 1); HRESIMS *m*/*z* 620.6454 [M–H]^−^ (C_16_H_6_Br_5_N_2_ calc. 620.6453, Δ 0.2 ppm).

### LC-MS/MS Data Acquisition

The HRMS data acquisition was performed on (A) a Q Exactive Plus mass spectrometer (large-scale cultures, purification) equipped with a heated ESI interface coupled to an UltiMate 3000 HPLC system or on (B) an Orbitrap Exploris 240 mass spectrometer (small scale cultures, FBMN based structure proposal) that was equipped with a heated ESI interface coupled to a Vanquish Flex HPLC system (all Thermo Fisher Scientific). The following chromatographic parameters were used: Kinetex C18 column (50 × 2.1 mm, 2.6 μm, 100 Å, Phenomenex), binary gradient from 5 to 100% MeCN in H_2_O (0.1% formic acid each) at 0.4 mL/min in 16 min, 100% MeCN for 4 min. HRMS data acquisition: pos. and neg. ionization mode, ESI spray voltage 3.5 and −2.5 kV, capillary temperature 350 °C (A) or 350 °C (B), sheath gas flow rate 50 L/min (A) or 40 L/min (B), auxiliary gas flow rate 12.5 L/min (A) or 5 L/min (B). Full scan spectra were acquired from *m*/*z* 133.4 to 2000 with a resolution of 35 000 at *m*/*z* 200, automated gain control (AGC) 5 × 10^5^, maximal injection time = 120 ms. MS/MS spectra were acquired in data-dependent acquisition mode (dd-MS^2^), stepped collision energy of 30, 60, and 75 eV (resulting at 55 eV), a resolution of 17 500 at *m*/*z* 200, an AGC of 2 × 10^5^, and a maximal injection time of 75 ms. A TopN experiment (*N* = 5, loop count = 5) was implemented for triggering dd-MS2 acquisition.

### File Conversion

Raw mass spectrometry data files were converted from .RAW to .mzML format, using MSConvert from ProteoWizard (version 3.0).^33^ A scan polarity filter was used during data conversion to separate positive ion mode scans from negative ion mode scans, facilitating a more targeted analysis in subsequent data analysis steps.

### MassQL Data Analysis

To identify scans in the mass spectrometry dataset containing iodinated AETX derivatives, a query was written to find MS2 peaks at *m*/*z* 126.9044 with a 10-ppm tolerance and a minimum percent intensity to the base peak of 1.0%. For the bromine-containing derivatives, a query was used that finds MS2 peaks at *m*/*z* 78.9183 or *m*/*z* 80.9162 with a 10-ppm tolerance and a minimum percent intensity to the base peak of 1.0%. The query results were filtered for a minimum intensity of 1 × 10^6^ for further analysis.^12^

### Classical and Feature Based Molecular Networking

To facilitate FBMN analysis, the converted mass spectrometry data were processed using MZmine (versions 3 and 4.2) with a workflow designed and executed through the MZWizard tool to automate the feature extraction and alignment.^34^ For mass detection, noise level thresholds of 5.00 (MS1, factor of the lowest signal) and 0.00 (MS2, factor of the lowest signal) were applied to discard low-intensity signals. Chromatogram building was performed with the following parameters: minimum consecutive scans = 4, minimum absolute height = 1 × 10^5^, *m*/*z* tolerance = 10 ppm.^35^ Chromatographic peaks were smoothed using a Savitzky-Golay filter (window size of 5 points) to reduce noise and refine the peak shape. The Join Aligner module for peak alignment was used with the following parameters: retention time tolerance = 0.4 min, *m*/*z* tolerance = 5 ppm. The molecular networks for CMN and FBMN analysis were generated using GNPS (http://gnps.ucsd.edu).^13^ All HRMS^2^ fragment ions within a 17 Da window of precursor *m*/*z* were excluded from the dataset. Subsequently, the HRMS^2^ spectra underwent further filtration, with only the six most prominent fragment ions within a 50 Da window across the spectrum being selected. The precursor ion mass tolerance was set at 0.02 Da, and the same 0.02 Da tolerance was applied to the HRMS^2^ fragment ions. The network was constructed with edges retained if they exhibited a cosine score exceeding 0.6 and a minimum of four matched peaks. Furthermore, edges between two nodes were retained only if each node appeared in the other’s list of the top ten most similar nodes. The maximum size of any molecular family was limited to 100, and the lowest scoring edges were removed until each molecular family was below this threshold.^13,14^

### Molecular Network Visualization

Molecular networks generated by the GNPS workflow were visualized and analyzed using Cytoscape (version 3.10.2).^36,37^ GNPS output, including network data (in GraphML format), was imported into Cytoscape for interactive visualization.

### Quantification by Evaporative Light Scattering Detection (ELSD)

To avoid weighing inaccuracies, the concentrations of test compound solutions for bioactivity testing were quantified using HPLC coupled with an evaporative light scattering detector (1290 Infinity II, Agilent), as described previously.^38^ Synthetic AETX^39^ was used as standard substance to establish a calibration curve (triplicate injection of 1 to 10 μL of a 23,7 ng/μL solution in 90% MeCN) on a Kinetex C18 column (100 × 3 mm, 2.6 μm, 100 Å, Phenomenex), eluted with a gradient from 10 to 100% MeCN in H_2_O (0.1% FA each) over 10 min at 0.65 mL/min. Settings of the ELSD were as follows: evaporator temperature = 45 °C, nebulizer temperature = 45 °C, gas flow rate = 1.3 SLM, and N_2_ pressure = 3.5 bar. The calibration curve was generated as described by Young et al.^40^ In brief, the response areas were averaged, and log­(ELSD response area) was plotted against log­(amount in nanograms) to generate a linear calibration curve. Compounds **1**, **3**, **4**, and **5** were dissolved in 1 mL MeCN 90% (v/v), diluted 1:3 in the same solvent and injected in triplicate under the same conditions.

### Cell Culture and Cytotoxicity Testing

HCT116 cells were maintained in a humidified atmosphere at 37 °C with 5% CO_2_. HCT116 cells were kept in McCoy’s 5A medium (Thermo Fisher Scientific) supplied with 10% FBS (Sigma–Aldrich) and Penicillin (10000 U/L)/Streptomycin (100 mg/L) (Roth). The sulforhodamine B (SRB) colorimetric assay was conducted as previously described by Vichai et al.^32^ Briefly, 2 × 10^4^ cells per well were seeded in a clear, cell-culture-treated 96-well plate with flat bottom (Greiner). The following day, cells were incubated for 24 h with three concentrations (0.1, 1, and 10 μM) of compounds **1**, **3**, **4**, and **5** and the respective solvent (0.1% DMSO) as control. After the incubation time, cells were directly fixed with cold 10% (w/v) trichloroacetic acid (Roth) for 1 h. Then, cells were carefully rinsed four times with slow-running tap water and blow dried. When cells were completely dry, 0.057% (w/v) SRB solution (Sigma–Aldrich) in 1% (v/v) acetic acid (ITW Reagents) was added to the wells. After 30 min of incubation at room temperature, cells were quickly washed four times with 1% (v/v) acetic acid and blow dried. Finally, 10 mM Tris base solution (pH 10.5) was added to the completely dry wells. The plate was placed in a TECAN Infinite M Plex plate reader, orbitally shaken for 300 s, and absorbance was recorded at 510 nm. The experiment was performed in three independent biological replicates. Results are presented as treatment over control in percentage. Statistical analysis of the data was conducted with an Origin 2021b instrument (OriginLab Corporation). After assessing normal distribution with the Lillifors test, the Mann–Whitney test was used to evaluate the differences between treatment and control, as well as between treatment with AETX and its derivatives.

## Supplementary Material



## Data Availability

NMR raw data have been archived at nmrXiv (https://10.57992/nmrxiv.p91).

## Notes

Additional information about the MassQL queries, FBMN evaluation, structures, HRMS, MS/MS spectra of all described AETX derivatives and biosynthetic intermediates; ^1^H, ^13^C, HSQC, HMBC, COSY spectra of compounds **1**–**5** (PDF)

## ∇

Helmholtz-Institute for Metabolic, Obesity and Vascular Research (HI-MAG), Helmholtz Center Munich at the University of Leipzig and the University Hospital Leipzig AöR, 04103 Leipzig, Germany
