# Supplementary material for: Mining for Halogenated Metabolites of Aetokthonos hydrillicola, the “Eagle Killer” Cyanobacterium
Source: J Nat Prod. 2025 May 16;88(6):1298–308. doi: 10.1021/acs.jnatprod.5c00161 (PMC12210266; doi:10.1021/acs.jnatprod.5c00161)
Supplement: Supplementary file 1 [file np5c00161_si_001.pdf]

Supporting Information

Mining for Halogenated Metabolites of *Aetokthonos hydrillicola*, the "Eagle Killer" Cyanobacterium

Franziska Schanbacher[1,†], *Valerie I. C. Rebhahn*[1], *Markus Schwark*[2], *Steffen Breinlinger*[2], *Lenka Štenclová*[1], *Kristin Röhrborn*[2], *Peter Schmieder*[3], *Heike Enke*[4], *Susan B. Wilde*[5], *Timo H. J. Niedermeyer*[1,†,*]

[1]Department of Pharmaceutical Biology, Institute of Pharmacy, Freie Universität Berlin, 14195 Berlin, Germany
[2]Department of Pharmaceutical Biology/Pharmacognosy, Institute of Pharmacy, Martin-Luther-University Halle-Wittenberg, 06120 Halle (Saale), Germany
[3]Leibniz-Forschungsinstitut für Molekulare Pharmakologie, Department of NMR-Supported Structural Biology, Berlin, Germany
[4]Simris Biologics GmbH, 12489 Berlin, Germany
[5]Warnell School of Forestry and Natural Resources, Fisheries and Wildlife, University of Georgia, Athens, GA 30602, USA *Corresponding author
[†]Part of this work was conducted at the Department of Pharmaceutical Biology/Pharmacognosy, Institute of Pharmacy, Martin-Luther-University Halle-Wittenberg, 06120 Halle (Saale), Germany

*Corresponding author timo.niedermeyer@fu-berlin.de

List of Figures

Figure S1. Base Peak Chromatograms of biomass extracts of *A. hydrillicola* 6

Figure S2. Chromatograms of the biomass extract of *A. hydrillicola*... 7

Figure S3. Chromatograms of the biomass extract of *A. hydrillicola*... 8

Figure S4. MassQL queries.. 9

Figure S5. $HRMS^2$ spectrum of compound **4** 17

Figure S6. $HRMS^2$ spectrum of compound $\mathbf{D_1}$ 17

Figure S7. $HRMS^2$ spectrum of compound $\mathbf{D_2}$ 17

Figure S8. $HRMS^2$ spectrum of compound $\mathbf{D_3}$ 17

Figure S9. $HRMS^2$ spectrum of compound **5** 19

Figure S10. $HRMS^2$ spectrum of compound **E**.. 19

Figure S11. $HRMS^2$ spectrum of compound **C**.. 20

Figure S12. $HRMS^2$ spectrum of compound **C**.. 20

Figure S13. $HRMS^2$ spectrum of compound **i** 21

Figure S14. $HRMS^2$ spectrum of compound $\mathbf{j_2}$.. 21

Figure S15. $HRMS^2$ spectrum of compound **F** 21

Figure S16. $HRMS^2$ spectrum of compound **k** collision energy 55 eV 23

Figure S17. $HRMS^2$ spectrum of compound **k** collision energy 75 eV. 23

Figure S18. $HRMS^2$ spectrum of compound $\mathbf{l_1}$ 24

Figure S19. $HRMS^2$ spectrum of compound $\mathbf{l_3}$.. 24

Figure S20. $HRMS^2$ spectrum of compound $\mathbf{l_3}$.. 24

Figure S21. $HRMS^2$ spectrum of compound $\mathbf{m_3}$ collision energy 55 eV 26

Figure S22. $HRMS^2$ spectrum of compound $\mathbf{m_3}$ collision energy 65 eV 26

Figure S23. HRMS^2 spectrum of compound **n$_2$** 28

Figure S24. HRMS^2 spectrum of compound **n$_3$** 28

Figure S25. HRMS^2 spectrum of compound **n$_3$** collision energy 65 eV, two further key fragments visible in two consecutive scans provide a hint at the substitution pattern. 28

Figure S26. HRMS^2 spectrum of compound **o$_2$** 30

Figure S27. FBMN chemical space visualization of AETX derivatives. 31

Figure S28. Systematic numbering AETX and compounds **1-5**. 32

Figure S29. ^{1}H NMR spectrum **1** in DMSO-*d$_6$*. 33

Figure S30. ^{1}H NMR spectrum **1** in DMSO-*d$_6$*. Zoomed in region between 7.2-13.2 ppm. 34

Figure S31. ^{1}H NMR spectrum **1** in DMSO-*d$_6$*. Zoomed in region between 7.6-8.5 ppm. 35

Figure S32. COSY NMR spectrum **1** in DMSO-*d$_6$*. Zoomed in region between 7.5-13.2 ppm.. 36

Figure S33. ^{13}C-HMQC NMR spectrum **1** in DMSO-*d$_6$*. Zoomed in region between 7.2-13.2 ppm. 37

Figure S34. ^{13}C-HMBC NMR spectrum **1** in DMSO-*d$_6$*. Zoomed in region between 7.5-8.6 ppm. 38

Figure S35. ^{1}H NMR spectrum **2** mixed with **1** in DMSO-*d$_6$*. 39

Figure S36. ^{1}H NMR spectrum **2** in DMSO-*d$_6$*. Zoomed in region between 7.5-13 ppm. 40

Figure S37. ^{13}C-HMQC NMR spectrum **2** in DMSO-*d$_6$*. Zoomed in region between 7.6-8.5 ppm. 41

Figure S38. ^{13}C-HMBC NMR spectrum **2** in DMSO-*d$_6$*. The signals that determine the position of iodination are highlighted. 42

Figure S39. ^{1}H NMR spectrum **3** in DMSO-*d$_6$*. 43

Figure S40. ^{1}H NMR spectrum **3** in DMSO-*d$_6$*. Zoomed in region between 6.5-8.0 ppm. 44

Figure S41. ^{1}H NMR spectrum **4** in DMSO-d_6.. 45

Figure S42. ^{1}H NMR spectrum **4** in DMSO-d_6. Zoomed in region between 6.5-8.0 ppm. 46

Figure S43. ^{13}C NMR spectrum **4** in DMSO-d_6. Whole spectral range..................................... 47

Figure S44. ^{13}C NMR spectrum **4** in DMSO-d_6. Zoomed in region between 90-150 ppm 48

Figure S45. COSY NMR spectrum **4** in DMSO-d_6. 49

Figure S46. COSY NMR spectrum (600 MHz) of compound **4** in DMSO-d_6. Zoomed in region between 6.8-7.7 ppm.. 50

Figure S47. ^{13}C-HMQC NMR spectrum **4** in DMSO-d_6. 51

Figure S48. ^{13}C-HMQC NMR spectrum **4** in DMSO-d_6. Zoomed in region between 6.7-8.0 ppm. 52

Figure S49. ^{13}C-HMBC NMR spectrum **4** in DMSO-d_6. 53

Figure S50. ^{13}C-HMBC NMR spectrum **4** in DMSO-d_6. Zoomed in region between 6.8-7.8 ppm. 54

Figure S51. ^{1}H NMR spectrum **5** in DMSO-d_6.. 55

Figure S52. ^{1}H NMR spectrum 5 in DMSO-d_6, after additional purification............................ 56

Figure S53. ^{1}H NMR spectrum **5** in DMSO-d_6. Zoomed in region between 6.5-8.5 ppm. 57

Figure S54. ^{13}C NMR spectrum **5** in DMSO-d_6. Whole spectral range..................................... 58

Figure S55. ^{13}C NMR spectrum **5** in DMSO-d_6. Zoomed in the region between 110-140 ppm. 59

Figure S56. ^{13}C-HMQC NMR spectrum 5 in DMSO-d_6. Zoomed in region between 6.7-8.5 ppm. 60

Figure S57. COSY NMR spectrum **5** in DMSO-d_6. Zoomed in region between 7.0-8.6 ppm.... 61

Figure S58. ^{13}C-HMBC NMR spectrum **5** in DMSO-d_6. Zoomed in region between 6.7-8.7 ppm. 62

List of Tables

Table S1. MassQL query results for brominated AETX derivatives 10

Table S2. MassQL query results for iodinated AETX derivatives 13

Table S3. MassQL query results for both iodinated and brominated AETX derivatives 15

Table S4. Annotation of key ions observed in the HRMS^2 spectra of compounds **4**, **D$_1$**, **D$_2$**, **D$_3$**. 18

Table S5. Annotation of key ions observed in the HRMS^2 spectra **5**, and **E**. 19

Table S6. Annotation of key ions observed in the HRMS^2 spectrum of compound **C**. 20

Table S7. Annotation of key ions observed in the HRMS^2 spectra of compound **h**, **i**, **j$_1$**, **j$_2$** and **F**.
.... 22

Table S8. Annotation of key ions observed in the HRMS^2 spectrum of compound **k**................ 23

Table S9. Annotation of key ions observed in the HRMS^2 spectra of compound **l$_1$**, **l$_2$**, and **l$_3$**. ... 25

Table S10. Annotation of key ions observed in the HRMS^2 spectrum of compound **m$_3$**. 27

Table S11. Annotation of key ions observed in the HRMS^2 spectra of compound **n$_1$** and **n$_2$**. 29

Table S12. Annotation of key ions observed in the HRMS^2 spectrum of compound **o$_2$**. 30

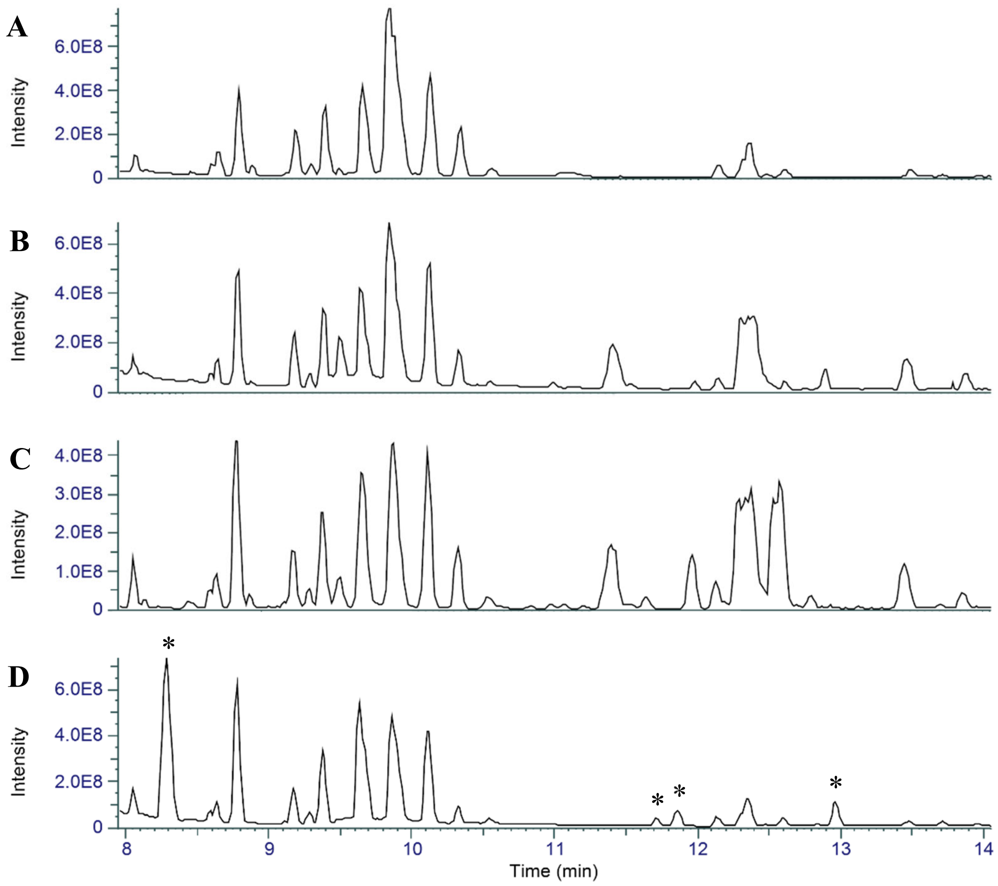

Figure S1. Base Peak Chromatograms of biomass extracts of *A. hydrillicola* (neg. ion mode, 8.0–14.0 min). **A** Unsupplemented control culture of *A. hydrillicola*. **B** *A. hydrillicola* grown in medium supplemented with KBr. **C** *A. hydrillicola* grown in medium supplemented with KBr and KI. **D** *A. hydrillicola* grown in medium supplemented with KI alone, * compounds not detected in the other samples.

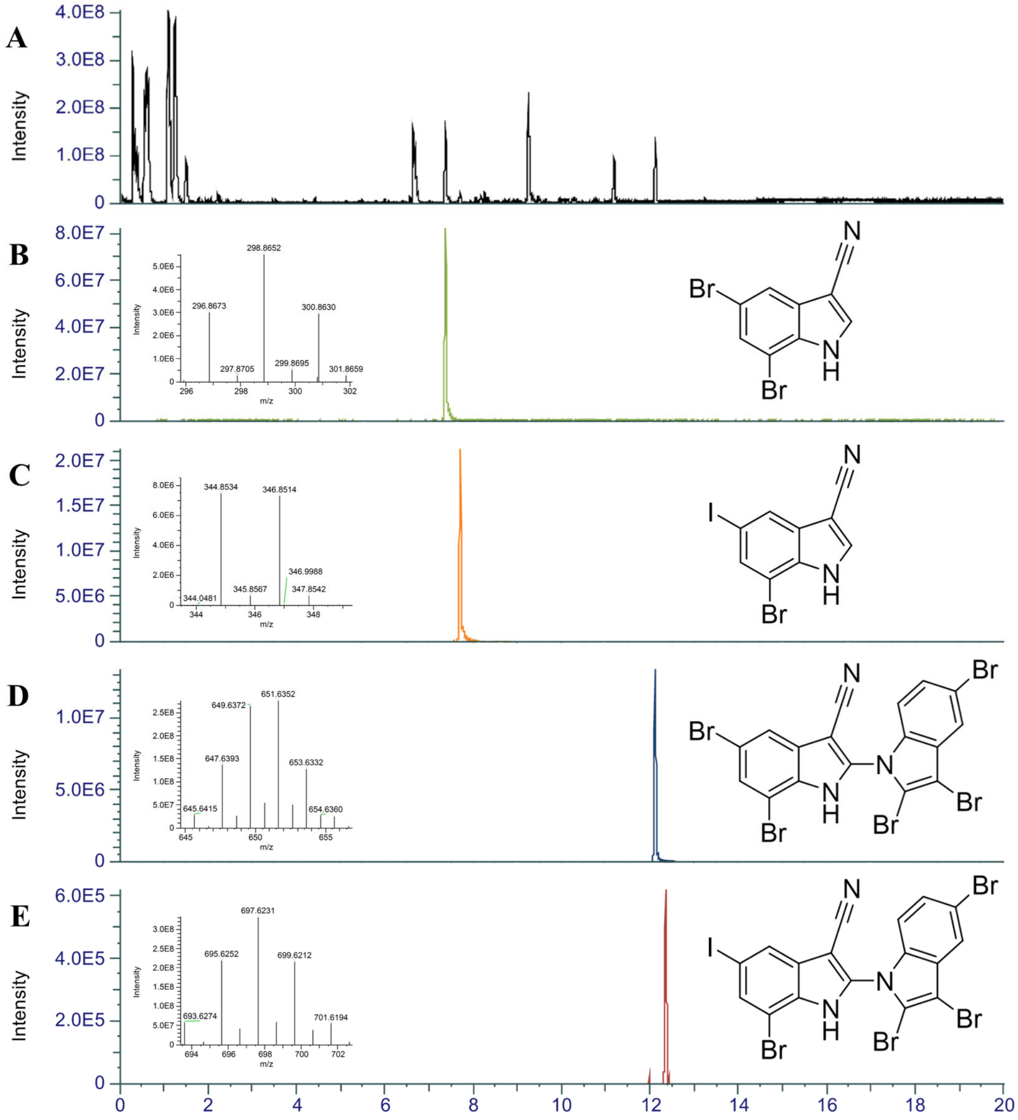

Figure S2. Chromatograms of the biomass extract of *A. hydrillicola* grown in a medium supplemented with both KBr and KI. **A** Base peak chromatogram (neg. ion mode). **B** Extracted ion chromatogram (EIC) of the western indole of AETX. **C** EIC of the monoiodinated western indole of AETX, localization of the iodine atom confirmed by NMR spectroscopy. **D** EIC of AETX. **E** EIC of monoiodinated AETX.

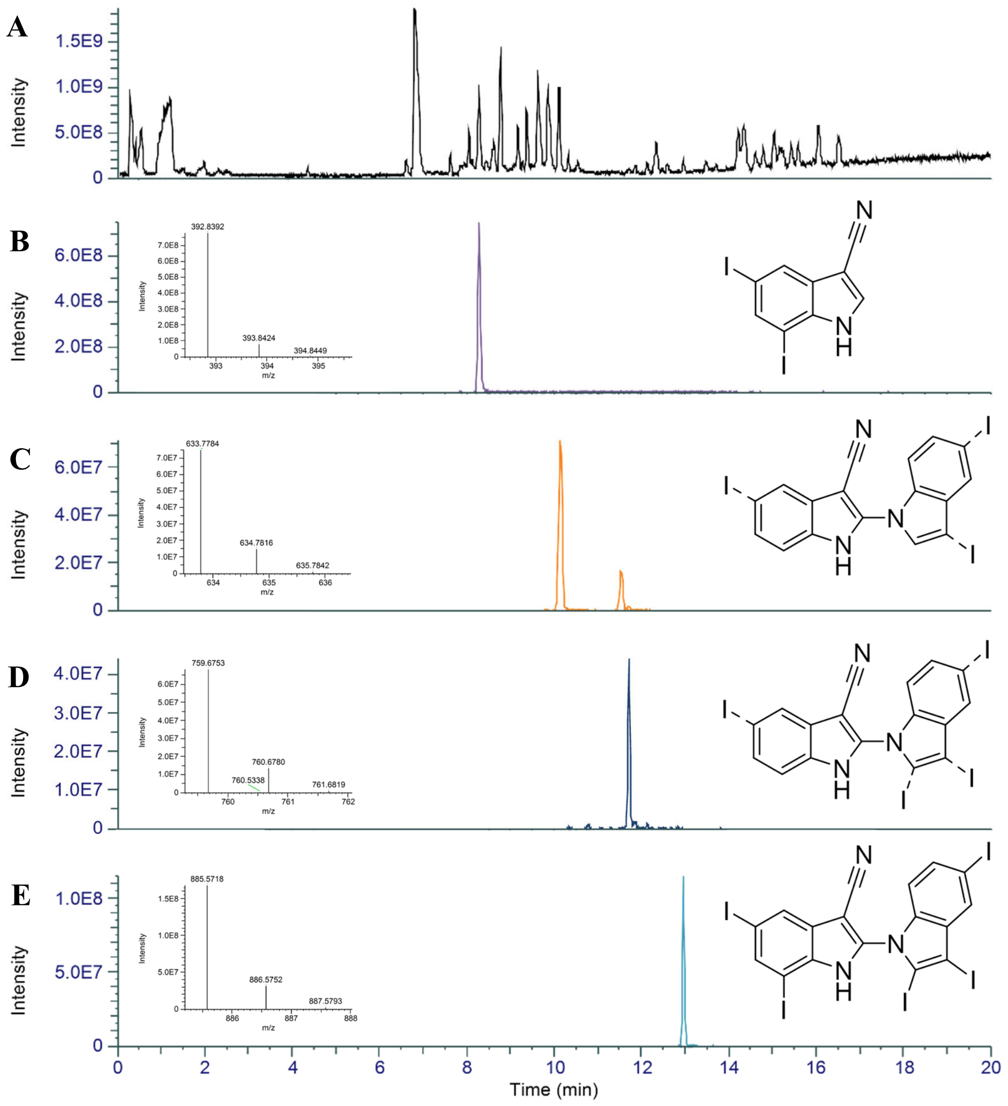

Figure S3. Chromatograms of the biomass extract of *A. hydrillicola* grown in a medium supplemented with KI alone. **A** Base peak chromatogram (neg. ion mode). **B** Extracted ion chromatogram (EIC) of the diiodinated western indole of AETX. **C** EIC of tetraiodinated AETX derivatives, **D** EIC of a triiodinated AETX derivative. **E** EIC of the pentaiodinated AETX derivative. The exact location of the iodine atoms or the substitution pattern for the tri- or tetraiodinated structural variants cannot be specified (indicated by dashed bonds).

**A**	**B**
QUERY scaninfo(MS2DATA) WHERE MS2PROD=126.9045:TOLERANCEPPM=10:INTENSITYPERCENT=1	QUERY scaninfo(MS2DATA) WHERE MS2PROD=78.9183:TOLERANCEPPM=10:INTENSITYPERCENT=1 and MS2PROD=80.9163:TOLERANCEPPM=10:INTENSITYPERCENT=1

Figure S4. MassQL queries to find iodinated (**A**) / brominated (**B**) analytes in the data set.

Table S1. MassQL query results for brominated AETX derivatives in all supplemented samples. Numbered compounds are supported by NMR data, proposed structures marked with an † are supported by HRMS^2 data. Compounds designated with numbers or capital letters are discussed in the manuscript.

	m/z [M-H] $^-$ (accurate mass)	*m/z* [M-H] $^-$ (exact mass)	t_R [min]	sum formula	proposed structure
**a**	314.8779	314.8774	5.34	$C_9H_6ON_2Br_2$ (Δ 1.6 ppm)	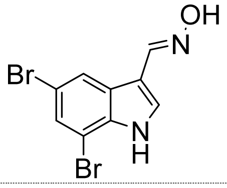
**b₁**	218.9554	218.9563	5.81	$C_9H_5N_2Br$ (Δ 4.1 ppm)	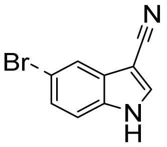
**b₂**	218.9554	218.9563	5.99	$C_9H_5N_2Br$ (Δ 4.1ppm)	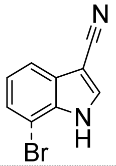
**1**	296.8670	296.8668	7.61	$C_9H_4N_2Br_2$ (Δ 0.7 ppm)	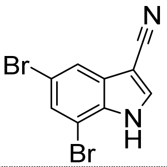
**c₁**	271.8719	271.8716	8.10	$C_8H_5NBr_2$ (Δ 1.1 ppm)	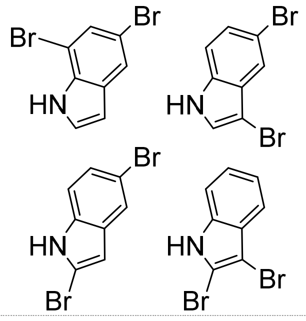
**c₂**	271.8726	271.8716	8.30	$C_8H_5NBr_2$ (Δ 2.2 ppm)	
**c₃**	271.8691	271.8716	8.40	$C_8H_5NBr_2$ (Δ 5.9 ppm)	
**3**	349.7821	349.7821	9.49	$C_8H_4NBr_3$ (Δ 0 ppm)	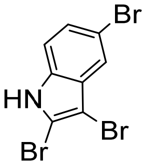

**B**	427.6928	427.6926	10.68	$C_8H_3NBr_4$ (Δ 0.5 ppm)	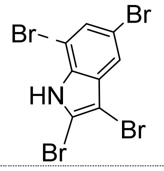
**D2**	567.7302	567.7301	10.98	$C_{17}H_7N_3Br_4$ (Δ 0.2 ppm)	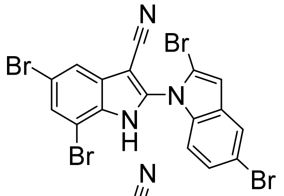
**D3**	567.7308	567.7301	11.52	$C_{17}H_7N_3Br_4$ (Δ 1.2 ppm)	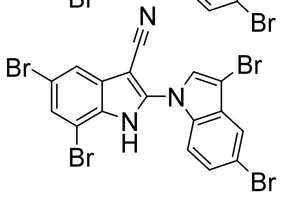
**4**	567.7299	567.7301	11.40	$C_{17}H_7N_3Br_4$ (Δ 0.4 ppm)	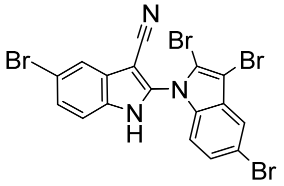
**D1**†	567.7302	567.7301	11.95	$C_{17}H_7N_3Br_4$ (Δ 0.2 ppm)	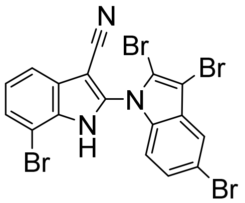
**AETX**	645.6409	645.6406	12.30	$C_{17}H_6N_3Br_5$ (Δ 0.5 ppm)	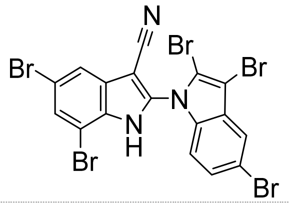
**E**†	620.6456	620.6453	13.12	$C_{16}H_7N_2Br_5$ (Δ 0.5 ppm)	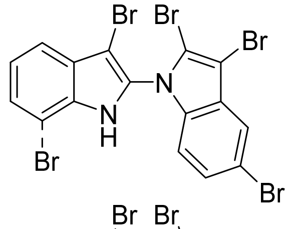 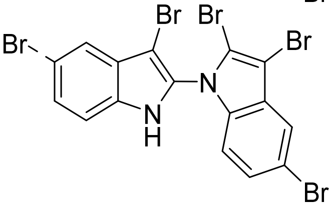

**5**	620.6454	620.6453	13.45	$C_{16}H_7N_2Br_5$ (Δ 0.2 ppm)	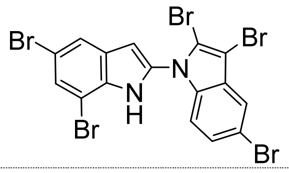
**C**†	698.5561	698.5558	13.86	$C_{16}H_6N_2Br_6$ (Δ 1.9 ppm)	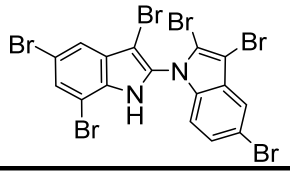

Table S2. MassQL query results for iodinated AETX derivatives in all supplemented samples. Numbered compounds are supported by NMR data, proposed structures marked with an † are supported by $HRMS^2$ data. Compounds designated with numbers or capital letters are discussed in the manuscript.

	m/z $[M-H]^-$ (accurate mass)	*m/z* $[M-H]^-$ (exact mass)	t_R [min]	sum formula	proposed structure
**d**	410.8502	410.8497	5.97	$C_9H_5N_2OI_2$ (Δ 1.2 ppm)	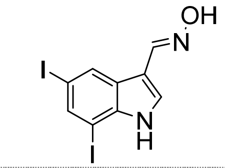
**e1**	266.9427	266.9425	6.46	$C_9H_5N_2I$ (Δ 0.7 ppm)	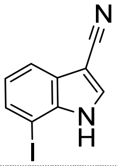
**e2**	266.9427	266.9425	6.63	$C_9H_5N_2I$ (Δ 0.7 ppm)	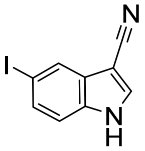
**G**	392.8392	392.8391	8.28	$C_9H_4N_2I_2$ (Δ 0.3 ppm)	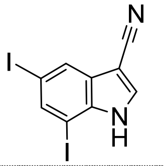
**f**	367.8440	367.8439	9.08	$C_8H_5NI_2$ (Δ 0.3 ppm)	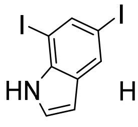 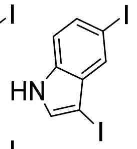 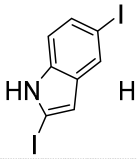 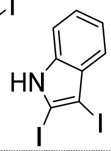
**g**	493.7400	493.7405	10.18	$C_8H_4NI_3$ (Δ 1.0 ppm)	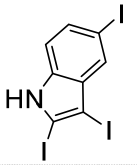
**h**	633.7784	633.7780	10.77	$C_{17}H_8N_3I_3$ (Δ 0.6 ppm)	-
**i**	633.7785	633.7780	11.72	$C_{17}H_8N_3I_3$ (Δ 0.8 ppm)	-
**j1†**	759.6753	759.6746	11.85	$C_{17}H_7N_3I_4$ (Δ 0.9 ppm)	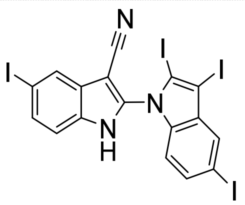

**j2**†	759.6756	759.6746	12.84	$C_{17}H_7N_3I_4$ (Δ 1.3 ppm)	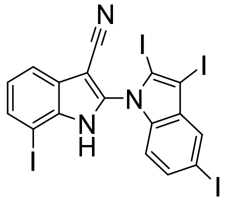
**F**	885.5718	885.5712	12.96	$C_{17}H_6N_3I_5$ (Δ 0.7 ppm)	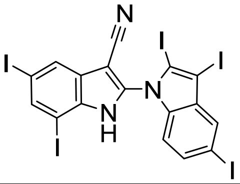

Table S3. MassQL query results for both iodinated and brominated AETX derivatives in all supplemented samples. Numbered compounds are supported by NMR data, proposed structures marked with an † are supported by HRMS^2 data. Compounds designated with numbers or capital letters are discussed in the manuscript.

	m/z [M-H] $^-$ (query results)	*m/z* [M-H] $^-$ (calc.)	t_R [min]	sum formula	proposed structure
**2**	344.8533	344.8530	7.95	$C_9H_4N_2BrI$ (Δ 0.9 ppm)	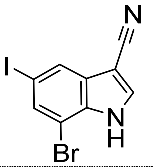
**k**†	585.7926	585.7918	11.56	$C_{17}H_8N_3BrI_2$ (Δ 1.4 ppm)	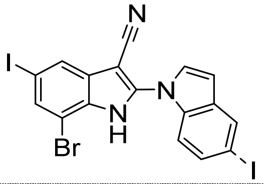
**l₁**†	615.7164	615.7162	11.23	$C_{17}H_7N_3Br_3I$ (Δ 0.3 ppm)	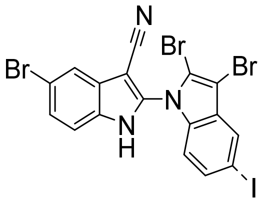
**l₂**†	615.7165	615.7162	11.64	$C_{17}H_7N_3Br_3I$ (Δ 0.5 ppm)	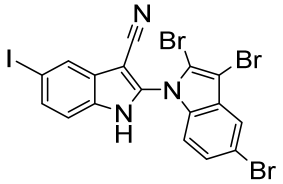
**l₃**†	615.7165	615.7162	12.19	$C_{17}H_7N_3Br_3I$ (Δ 0.5 ppm)	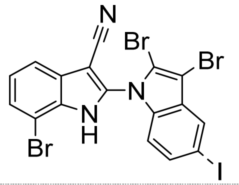
**m₁**	711.6889	711.6885	11.63	$C_{17}H_7N_3BrI_3$ (Δ 0.6 ppm)	-
**m₂**	711.6893	711.6885	11.79	$C_{17}H_7N_3BrI_3$ (Δ 1.1 ppm)	-
**m₃**†	711.6888	711.6885	11.93	$C_{17}H_7N_3BrI_3$ (Δ 0.4 ppm)	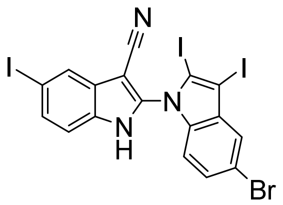

**m_4**	711.6892	711.6885	12.66	$C_{17}H_7N_3BrI_3$ (Δ 1.0 ppm)	-
**A**	693.6270	693.6267	12.57	$C_{17}H_6N_3Br_4I$ (Δ 0.4 ppm)	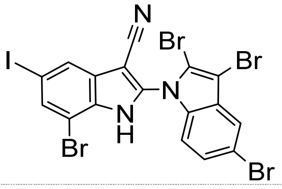
**n_1**	741.6127	741.6128	12.43	$C_{17}H_6N_3Br_3I_2$ (Δ 0.1 ppm)	-
**n_2**	741.6130	741.6128	12.66	$C_{17}H_6N_3Br_3I_2$ (Δ 0.3 ppm)	-
**n_3**†	741.6132	741.6128	12.80	$C_{17}H_6N_3Br_3I_2$ (Δ 0.5 ppm)	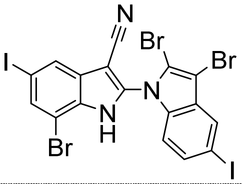
**o_1**	837.5864	837.5851	12.74	$C_{17}H_6N_3BrI_4$ (Δ 1.6 ppm)	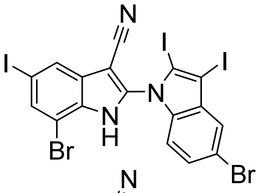
**o_2**	837.5864	837.5851	12.89	$C_{17}H_6N_3BrI_4$ (Δ 1.6 ppm)	
**o_3**	837.5862	837.5851	13.10	$C_{17}H_6N_3BrI_4$ (Δ 1.3 ppm)	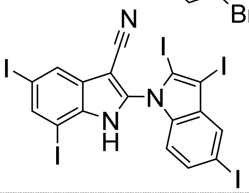
**p**	668.6307	668.6315	13.69	$C_{16}H_6N_2Br_4I$ (Δ 1.2 ppm)	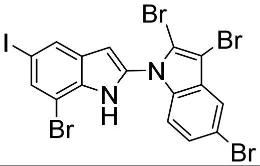

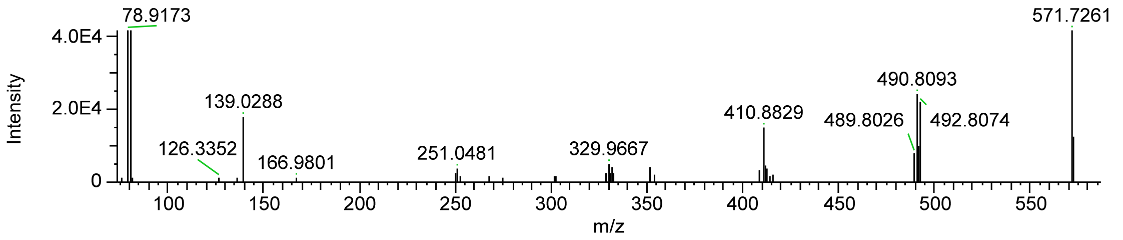

Figure S5. HRMS^2 spectrum of compound **4** (precursor ion *m/z* 571.7261). ESI neg. mode, collision energy 55 eV.

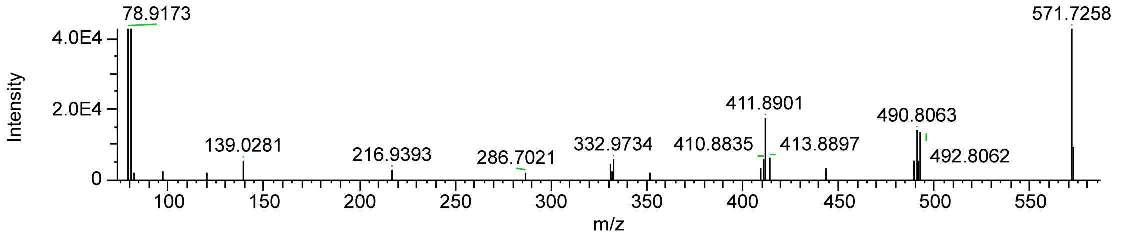

Figure S6. HRMS^2 spectrum of compound **D$_1$** (precursor ion *m/z* 571.7258). ESI neg. mode, collision energy 55 eV.

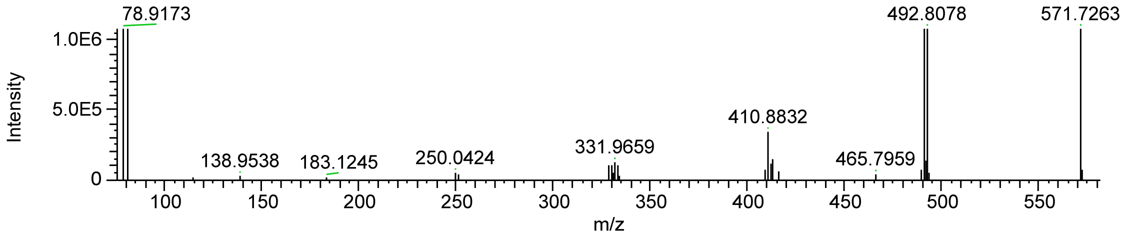

Figure S7. HRMS^2 spectrum of compound **D$_2$** (precursor ion *m/z* 571.7263). ESI neg. mode, collision energy 55 eV.

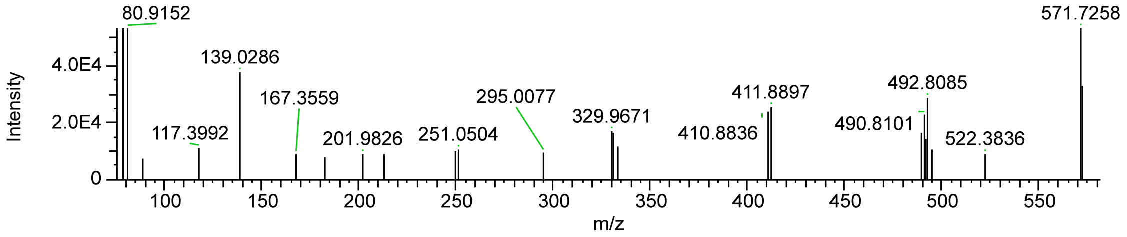

Figure S8. HRMS^2 spectrum of compound **D$_3$** (precursor ion *m/z* 571.7258). ESI neg. mode, collision energy 55 eV.

Table S4. Annotation of key ions observed in the HRMS[2] spectra of compounds **4**, **D₁**, **D₂**, **D₃**. Mass deviation from exact mass in parentheses.

m/z **4**	*m/z* **D1**	*m/z* **D2**	*m/z* **D3**	ion	proposed structure
571.7261 (Δ 1.0 ppm)	571.7263 (Δ 0.5 ppm)	571.7258 (Δ 0.4 ppm)	571.7258 (Δ 1.0 ppm)	**[M-H]⁻**	
490.8097 (Δ 0.9 ppm)	490.8096 (Δ 0.3 ppm)	490.8101 (Δ 0.8 ppm)	490.8063 (Δ 7.0 ppm)	**[M-H-Br]⁻**	
411.8905 (Δ 2.2 ppm)	411.8915 (Δ 0.2 ppm)	411.8897 (Δ 4.1 ppm)	411.8901 (Δ 3.2 ppm)	**[M-H-2Br]⁻**	
351.7795 (Δ 1.6 ppm)	-	-	351.7825 (Δ 6.9 ppm)	**$C_8H_3NBr_3^-$**	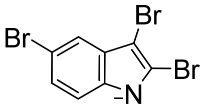
332,9733 (Δ 0.6 ppm)	332.9722 (Δ 2.7 ppm)	332.9703 (Δ 8.4 ppm)	332.9734 (Δ 0.9 ppm)	**[M-H-3Br]⁻**	
251.0481 (Δ 3.2 ppm)	251.0491 (Δ 0.8 ppm)	251.0504 (Δ 6.0 ppm)	-	**[M-2H-4Br]⁻**	
139.0288	139.1005	139.0286	139.0288	**Indole nitrile residue**	

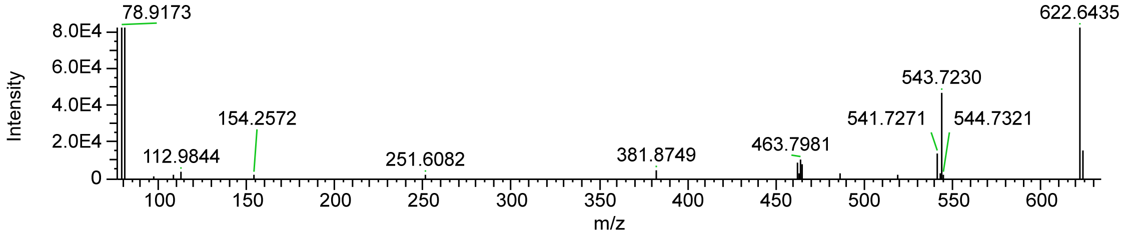

Figure S9. HRMS^2 spectrum of compound **5** (precursor ion *m/z* 622.6435). ESI neg. mode, collision energy 55 eV.

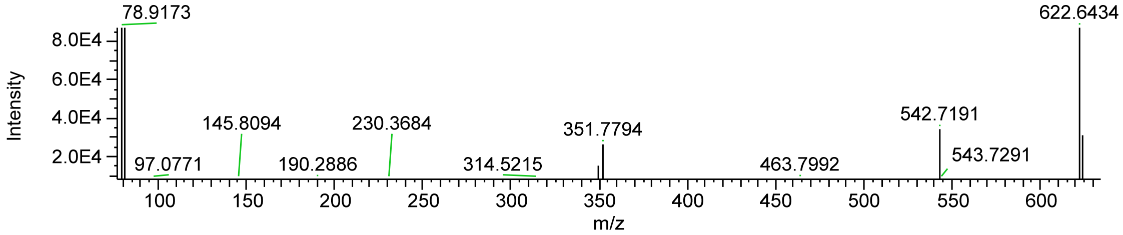

Figure S10. HRMS^2 spectrum of compound **E** (precursor ion *m/z* 622.6434). ESI neg. mode, collision energy 55 eV.

Table S5. Annotation of key ions observed in the HRMS^2 spectra of compound **5**, and compound **E**. Mass deviation from exact mass in parentheses.

m/z **5**	*m/z* **E**	ion	proposed structure
622.6435 (Δ 0.4 ppm)	622.6434 (Δ 0.2 ppm)	**$[M-H]^-$**	
543,7230 (Δ 3.5 ppm)	-	**$[M-H-Br]^-$**	
-	542.7191 (Δ 3.6 ppm)	**$[M-2H-Br]^-$**	
463.7981 (Δ 0.9 ppm)	463.7992 (Δ 0.9 ppm)	**$[M-2H-2Br]^-$**	
381.8749 (Δ 0.5 ppm)	-	**$[M-2H-3Br]^-$**	
-	351.7794 (Δ 1.9 ppm)	**$C_8H_3NBr_3^-$**	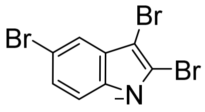

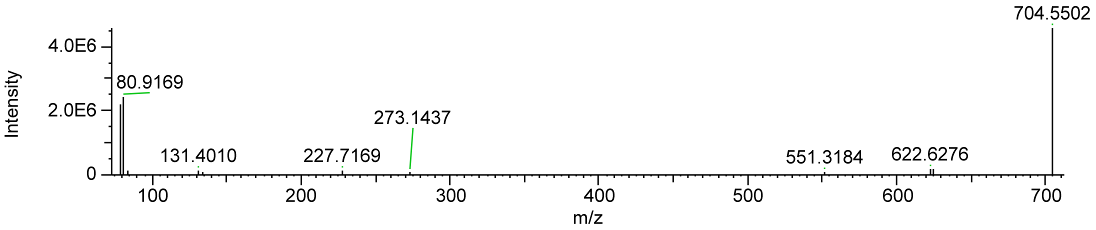

Figure S11. HRMS^2 spectrum of compound **C** (precursor ion *m/z* 704.5502). ESI neg. mode, collision energy 55 eV.

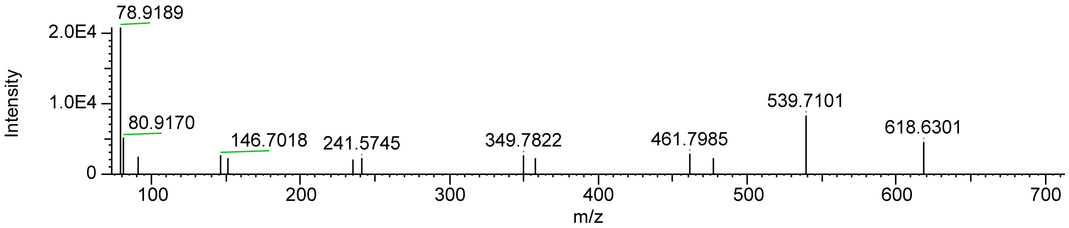

Figure S12. HRMS^2 spectrum of compound **C** (precursor ion *m/z* 704.5502). ESI neg. mode, collision energy 65 eV.

Table S6. Annotation of key ions observed in the HRMS^2 spectrum of compound **C**. Mass deviation from exact mass in parentheses.

m/z **C**	ion	proposed structure
704.5502 (Δ 0.7 ppm)	**[M-H]$^-$**	
618.6301 (Δ 0.7 ppm)	**[M-2H-Br]$^-$**	
539.7101 (Δ 2.3 ppm)	**[M-2H-2Br]$^-$**	
461.7985 (Δ 6.3 ppm)	**[M-H-3Br]$^-$**	
349.7822 (Δ 0.3 ppm)	**$C_8H_3NBr_3^-$**	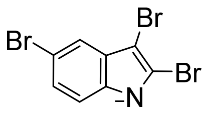

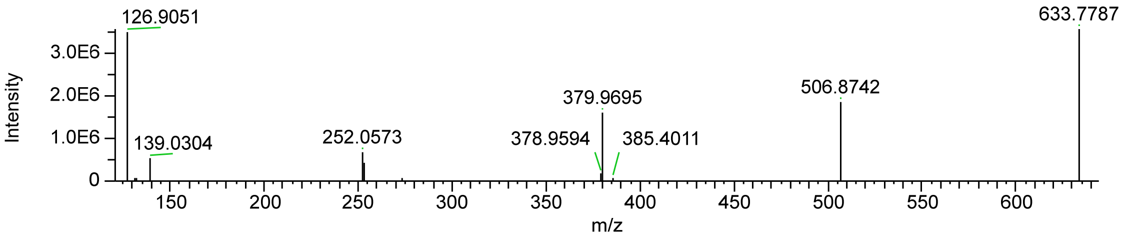

Figure S13. HRMS^2 spectrum of compound **i** (precursor ion *m/z* 633.7784). ESI neg. mode, collision energy 55 eV.

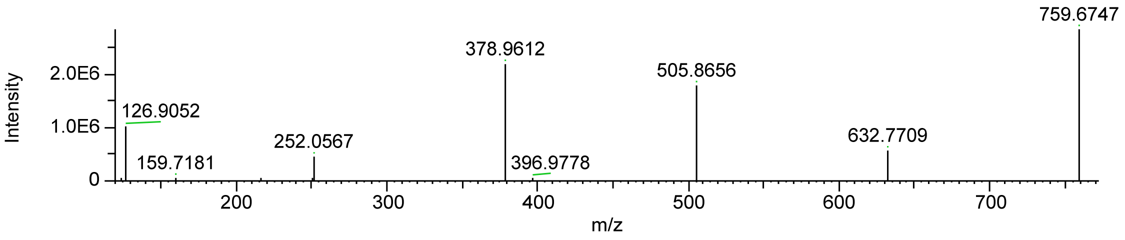

Figure S14. HRMS^2 spectrum of compound $\mathbf{j_2}$ (precursor ion *m/z* 759.6747). ESI neg. mode, collision energy 55 eV.

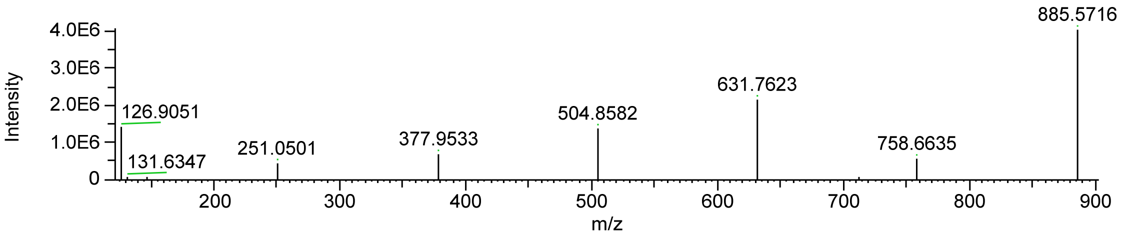

Figure S15. HRMS^2 spectrum of compound **F** (precursor ion *m/z* 885.5716). ESI neg. mode, collision energy 55 eV.

Table S7. Annotation of key ions observed in the HRMS^2 spectra of compound **h**, **i**, **j$_1$**, **j$_2$** and **F**. Mass deviation from exact mass in parentheses.

m/z **h**	*m/z* **i**	*m/z* **j$_1$**	*m/z* **j$_2$**	*m/z* **F**	ion
633.7784 (Δ 0.6 ppm)	633.7787 (Δ 1.1 ppm)	759.6741 (Δ 0.8 ppm)	759.6747 (Δ 0.1 ppm)	885.5716 (Δ 0.5 ppm)	**$[M-H]^-$**
506.8717 (Δ 3.6 ppm)	506.8742 (Δ 1.4 ppm)	632.7715 (Δ 3.8 ppm)	632.7709 (Δ 1.3 ppm)	758,6635 (Δ 4.2 ppm)	**$[M-H-I]^-$**
379.9694 (Δ 1.1 ppm)	379.9695 (Δ 1.3 ppm)	505.8663 (Δ 1.4 ppm)	505.8656 (Δ 0 ppm)	631,7623 (Δ 0.2 ppm)	**$[M-H-2I]^-$**
253.0653 (Δ 3.2 ppm)	253.0658 (Δ 5.1 ppm)	378.9617 (Δ 1.6 ppm)	378.9612 (Δ 0.3 ppm)	504,8582 (Δ 1.0 ppm)	**$[M-H-3I]^-$**
-	-	252.0571 (Δ 2.0 ppm)	252.0567 (Δ 0.4 ppm)	377,9533 (Δ 0.3 ppm)	**$[M-H-4I]^-$**
-	-	-	-	251.0501 (Δ 5.6 ppm)	**$[M-H-5I]^-$**

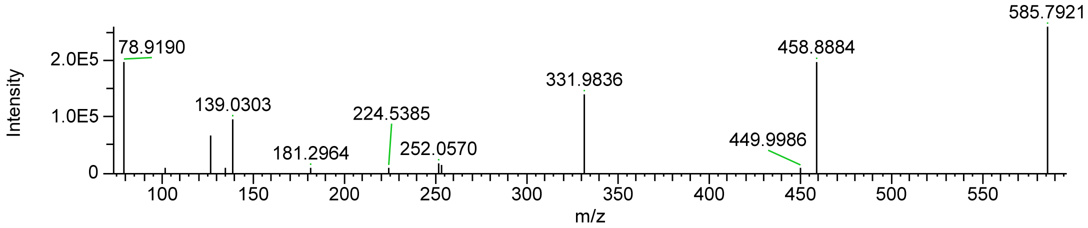

Figure S16. HRMS^2 spectrum of compound **k** (precursor ion *m/z* 585.7921). ESI neg. mode, collision energy 55 eV.

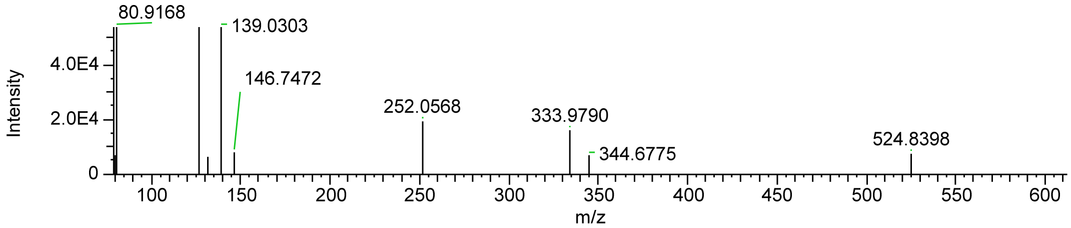

Figure S17. HRMS^2 spectrum of compound **k** (precursor ion *m/z* 587.7905). ESI neg. mode, collision energy 75 eV.

Table S8. Annotation of key ions observed in the HRMS^2 spectrum of compound **k**. Mass deviation from exact mass in parentheses.

m/z **k**	ion	proposed structure
585.7921 (Δ 0.5 ppm)	**[M-H]$^-$**	
458.8884 (Δ 2.4 ppm)	**[M-H-I]$^-$**	
331.9836 (Δ 1.0 ppm)	**[M-H-2I]$^-$**	
252.0570 (Δ 1.3 ppm)	**[M-2H-Br-2I]$^-$**	
344.6775 (Δ >5 ppm)	**$C_9H_3N_2BrI^-$**	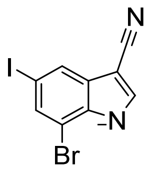
139.0303 (Δ 0.7 ppm)	**Indole nitrile residue**	

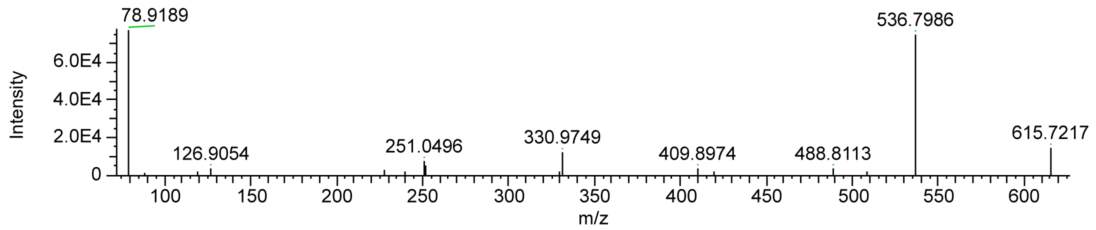

Figure S18. $HRMS^2$ spectrum of compound $\mathbf{l_1}$ (precursor ion *m/z* 615.7217). ESI neg. mode, collision energy 55 eV.

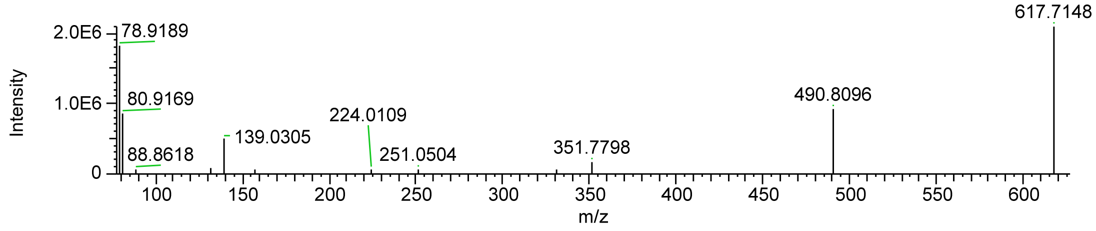

Figure S19. $HRMS^2$ spectrum of compound $\mathbf{l_3}$ (precursor ion *m/z* 615.7148). ESI neg. mode, collision energy 55 eV.

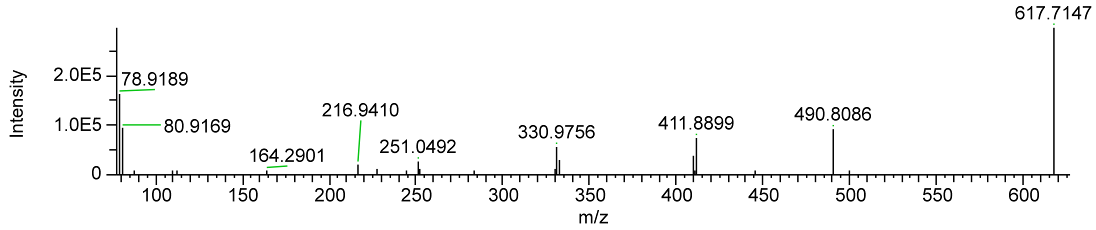

Figure S20. $HRMS^2$ spectrum of compound $\mathbf{l_3}$ (precursor ion *m/z* 615.7147). ESI neg. mode, collision energy 55 eV.

Table S9. Annotation of key ions observed in the HRMS^2 spectra of compound **l$_1$**, **l$_2$**, and **l$_3$**. Mass deviation from exact mass in parentheses.

m/z **l$_1$**	*m/z* **l$_2$**	*m/z* **l$_3$**	ion	proposed structure
615.7217 (Δ 8.9 ppm)	617.7148 (Δ 1.0 ppm)	617.7147 (Δ 0.9 ppm)	**[M-H]$^-$**	
536.7986 (Δ 1.4 ppm)	-	-	**[M-H-Br]$^-$**	
488.8113 (Δ 0.8 ppm)	490.8096 (Δ 0.1 ppm)	490.8086 (Δ 2.2 ppm)	**[M-H-I]$^-$**	
409.8974 (Δ 9.8 ppm)	-	409.8930 (Δ 0.9 ppm)	**[M-H-Br-I]$^-$**	
-	351.7798 (Δ 0.9 ppm)	-	**$C_8H_3NBr_3^-$**	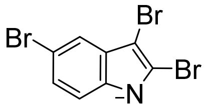
330.9749 (Δ 0.4 ppm)	330.9737 (Δ 4.0 ppm)	330.9756 (Δ 1.7 ppm)	**[M-H-Br$_2$-I]$^-$**	
251.0496 (Δ 2.8 ppm)	251.0504 (Δ 6.0 ppm)	251.9410 (Δ 1.2 ppm)	**[M-2H-Br$_3$-I]$^-$**	
-	-	216.9410 (Δ 0.9 ppm)	**$C_9H_2N_2Br^-$**	

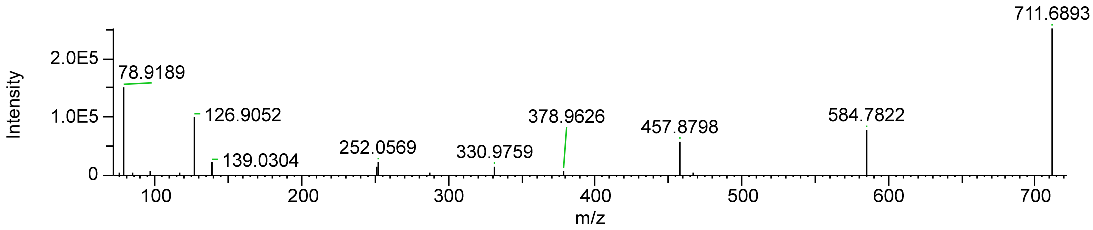

Figure S21. HRMS^2 spectrum of compound $\mathbf{m_3}$ (precursor ion *m/z* 711.6893). ESI neg. mode, collision energy 55 eV, the spectrum is given as an example; the spectra of all isomers (compounds $\mathbf{m_1}$-$\mathbf{m_4}$) show the same key fragments.

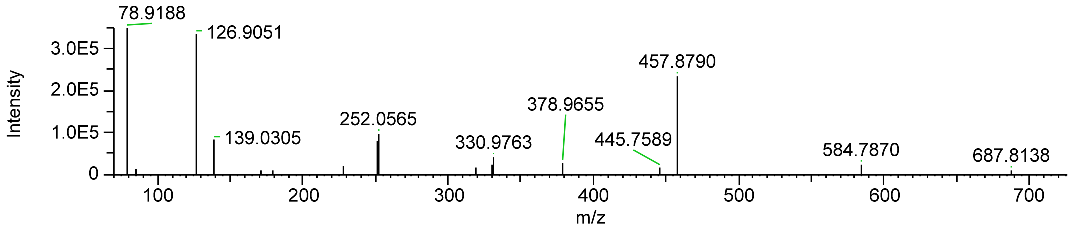

Figure S22. HRMS^2 spectrum of compound $\mathbf{m_3}$ (precursor ion *m/z* 711.6893). ESI neg. mode, collision energy 65 eV.

Table S10. Annotation of key ions observed in the HRMS^2 spectrum of compound $\mathbf{m_3}$. Mass deviation from exact mass in parentheses.

m/z $\mathbf{m_3}$ at 55 eV	ion	proposed structure
711.6893 (Δ 1.1 ppm)	**$[M\text{-}H]^-$**	
584.7822 (Δ 3.1 ppm)	**$[M\text{-}H\text{-}I]^-$**	
457,8798 (Δ 0.7 ppm)	**$[M\text{-}H\text{-}2I]^-$**	
378.9626 (Δ 2.7 ppm)	**$[M\text{-}H\text{-}Br\text{-}2I]^-$**	
330.9759 (Δ 3.8 ppm)	**$[M\text{-}H\text{-}3I]^-$**	
251.0484 (Δ 2.0 ppm)	**$[M\text{-}H\text{-}Br\text{-}3I]^-$**	
m/z **VII** at 65 eV	mol. formula	proposed structure
445.7589 (Δ 10.1 ppm)	**$C_8H_3NBrI_2^-$**	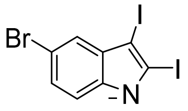

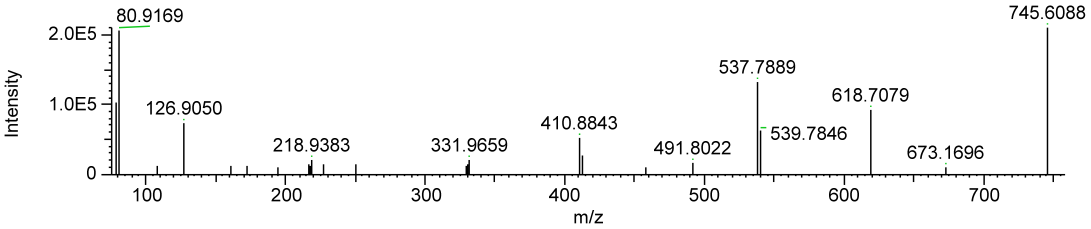

Figure S23. HRMS^2 spectrum of compound $\mathbf{n_2}$ (precursor ion *m/z* 745.6088). ESI neg. mode, collision energy 55 eV.

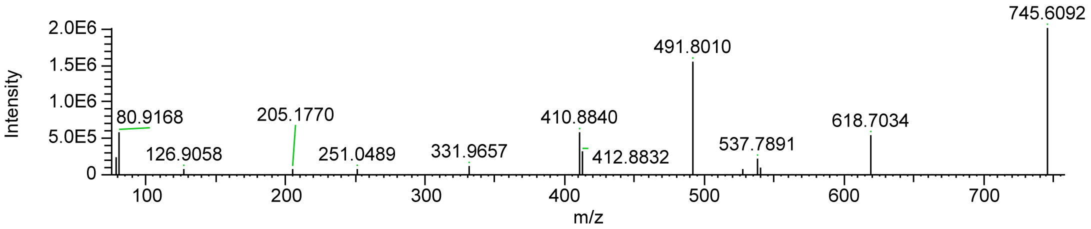

Figure S24. HRMS^2 spectrum of compound $\mathbf{n_3}$ (precursor ion *m/z* 745.6092). ESI neg. mode, collision energy 55 eV.

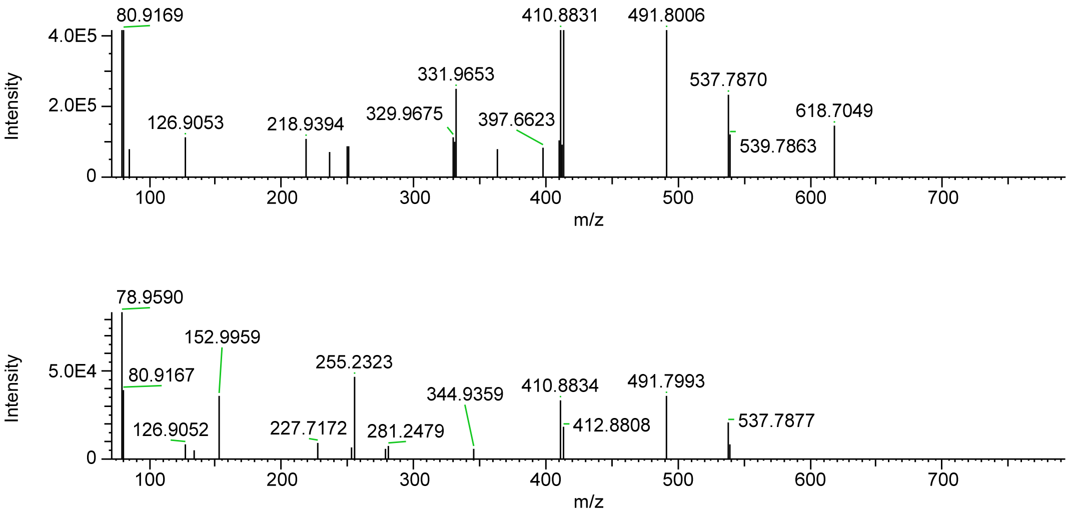

Figure S25. HRMS^2 spectrum of compound $\mathbf{n_3}$ (precursor ion *m/z* 745.6089). ESI neg. mode, collision energy 65 eV, two further key fragments visible in two consecutive scans provide a hint at the substitution pattern.

Table S11. Annotation of key ions observed in the HRMS² spectra of compound **n₁** and **n₂**. Mass deviation from exact mass in parentheses.

m/z **n₁**	*m/z* **n₂**	ion	proposed structure
745.6088 (Δ 0.1 ppm)	745.6088 (Δ 0.1 ppm)	**[M-H]⁻**	
618.7079 (Δ 5.9 ppm)	618.7034 (Δ 1.3 ppm)	**[M-H-I]⁻**	
491.8022 (Δ 5.0 ppm)	491.7997 (Δ 2.6 ppm)	**[M-H-2I]⁻**	
537.7889 (Δ 1.8 ppm)	537.7891 (Δ 2.7 ppm)	**[M-H-Br-I]⁻**	
410.8843 (Δ 2.1 ppm)	410.8840 (Δ 3.8 ppm)	**[M-H-Br-2I]⁻**	
331.9659 (Δ 2.4 ppm)	331.9651 (Δ 1.8 ppm)	**[M-H-2Br-2I]⁻**	
-	251.0489 (Δ 0 ppm)	**[M-H-3Br-2I]⁻**	
	m/z **XII** at 65 eV	mol. formula	proposed structure
-	344.9359 (Δ > 10.0 ppm)	**$C_9H_3N_2BrI^-$**	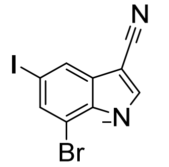
-	397.6623 (Δ > 10.0 ppm)	**$C_8H_3NBr_2I^-$**	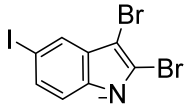

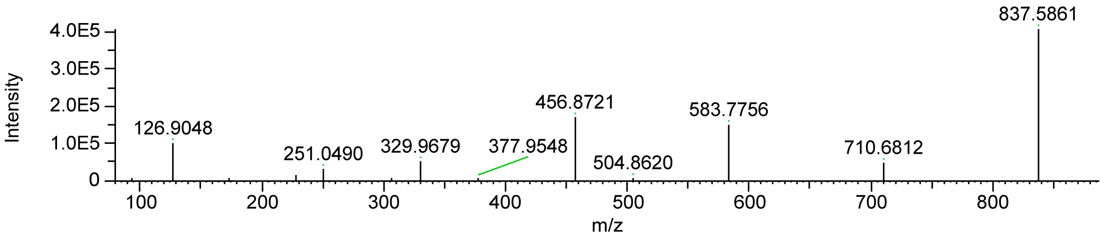

Figure S26. HRMS^2 spectrum of compound **o$_2$** (precursor ion *m/z* 837.5861). ESI neg. mode, collision energy 55 eV, the spectrum is given as an example; the spectra of all isomers (compounds **o$_1$-o$_3$**) show the same key fragments.

Table S12. Annotation of key ions observed in the HRMS^2 spectrum of compound **o$_2$**. Mass deviation from exact mass in parentheses.

m/z<br>**o$_2$**	ion	proposed structure
837.5861<br>(Δ 1.2 ppm)	**[M-H]$^-$**	
710.6812<br>(Δ 0.8 ppm)	**[M-H-I]$^-$**	
583.7756<br>(Δ 0.9 ppm)	**[M-H-2I]$^-$**	
456.8721<br>(Δ 1.1 ppm)	**[M-H-3I]$^-$**	
337.9548<br>(Δ 4.1 ppm)	**[M-H-Br-3I]$^-$**	
329.9679<br>(Δ 2.4 ppm)	**[M-H-4I]$^-$**	
251.0490<br>(Δ 0.4 ppm)	**[M-H-Br-4I]$^-$**	

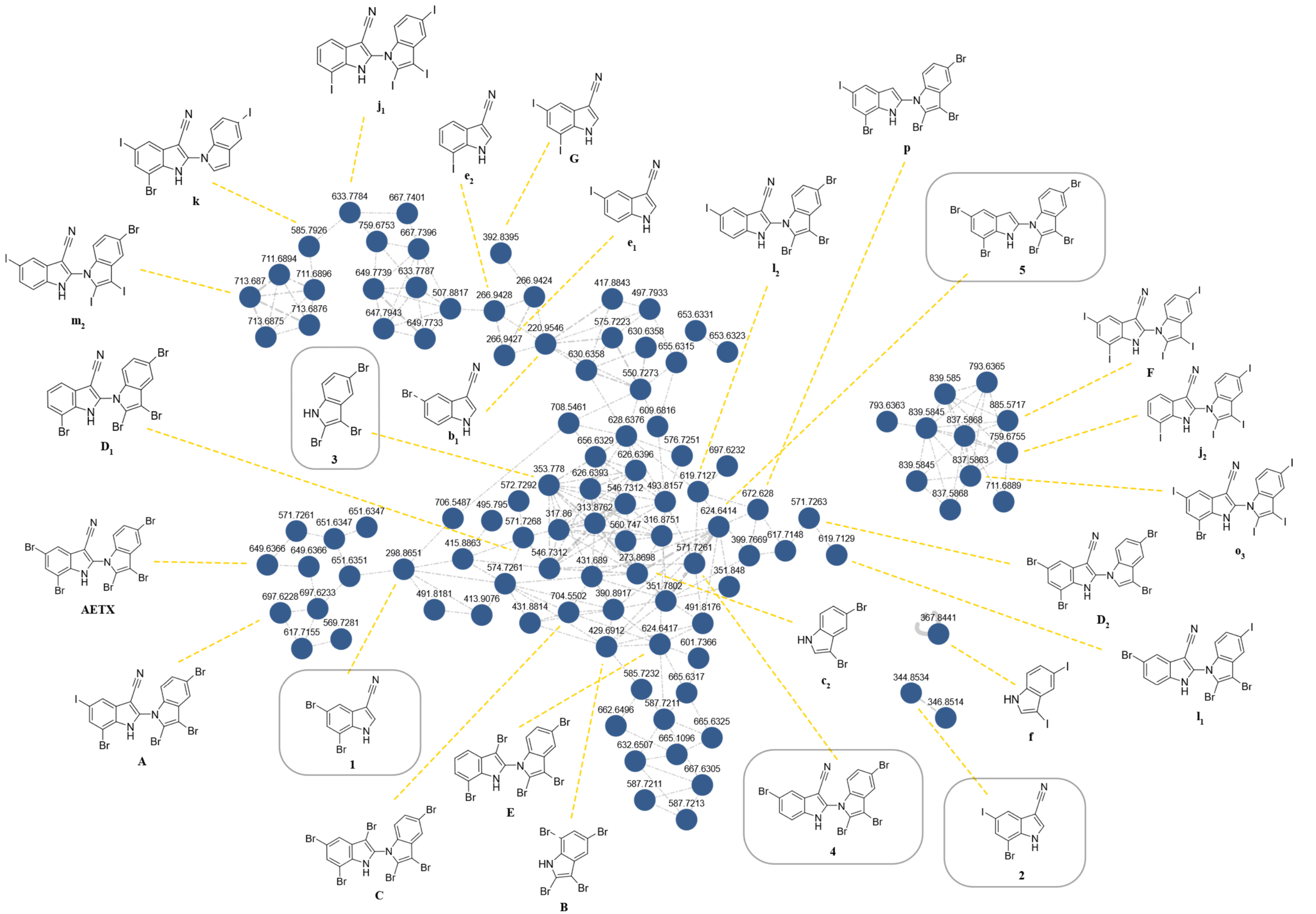

Figure S27. FBMN for structure prediction and chemical space visualization of AETX derivatives. Only one proposed structure is shown when the substitution pattern of isomers cannot be determined. Nodes connected for cosine scores ≥0.7, edge width correlating to the cosine score. Structures supported by NMR data are highlighted.

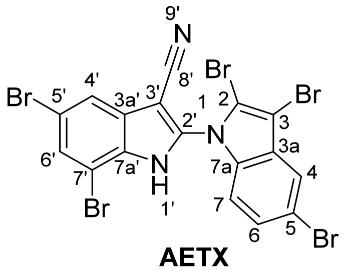

AETX

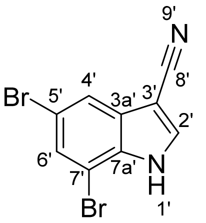

1

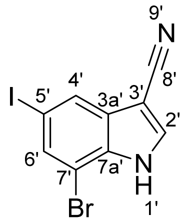

2

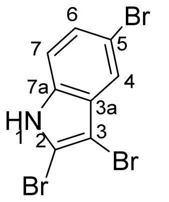

3

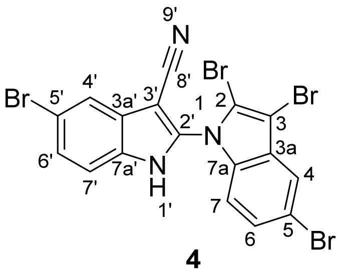

4

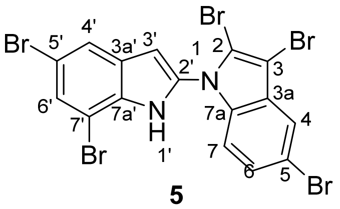

5

Figure S28. Systematic numbering of atoms in the scaffolds of AETX and compounds **1-5**. Numbering based on Breinlinger *et al.*

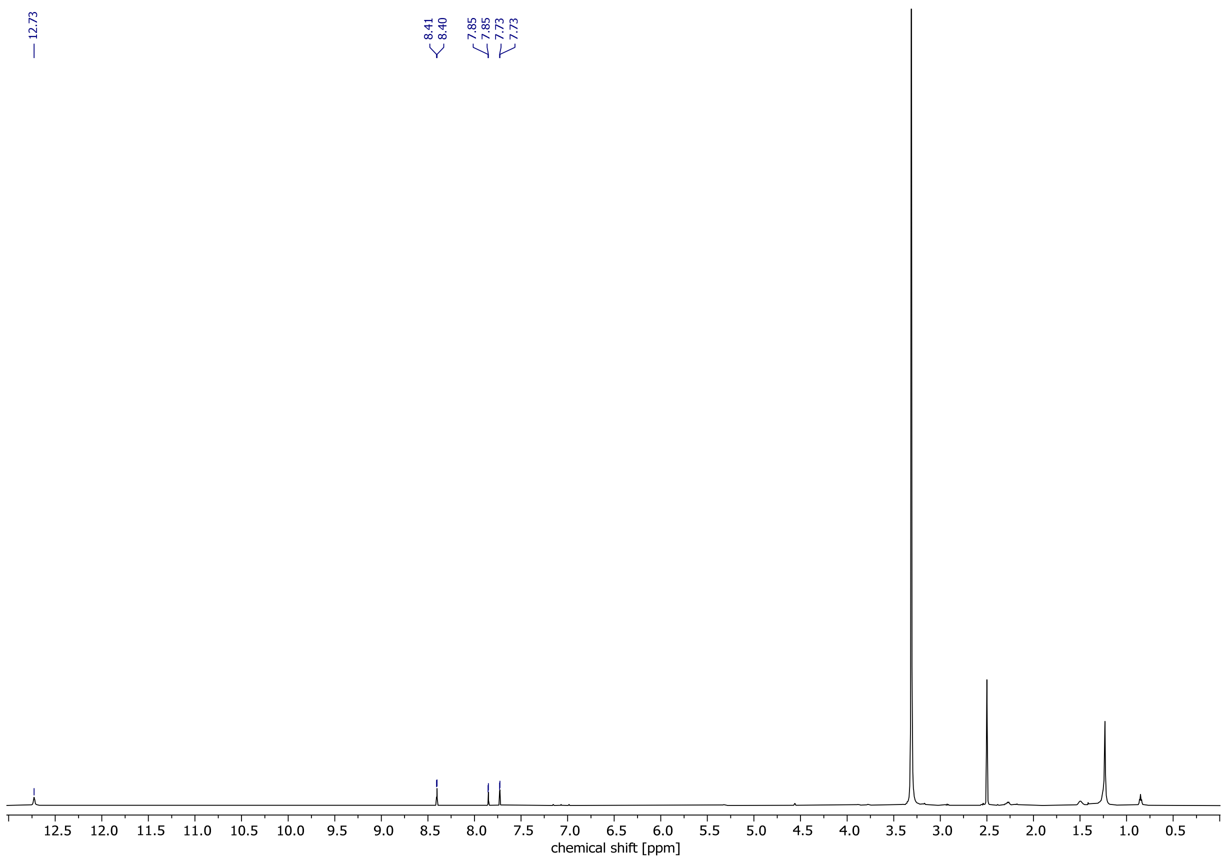

Figure S29. ^{1}H NMR spectrum (600 MHz) of compound **1** in DMSO-d_6.

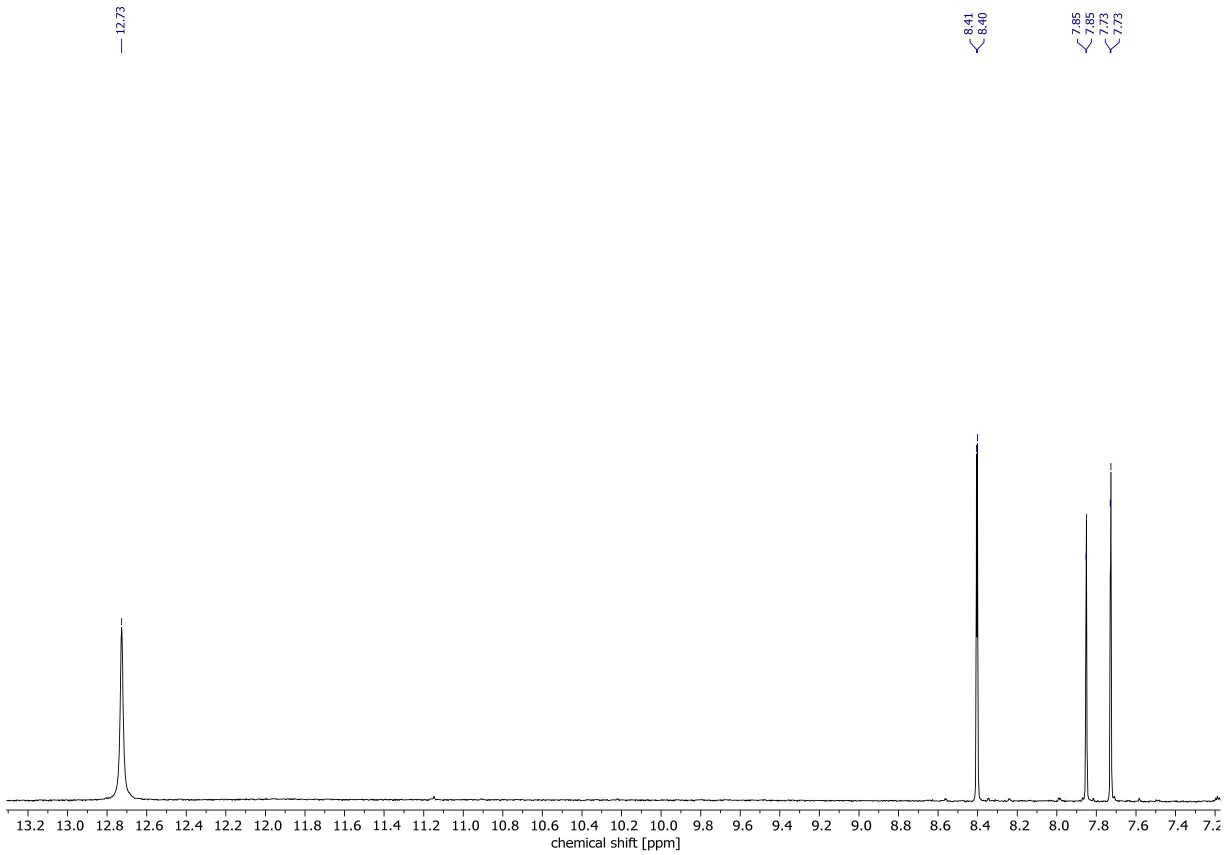

Figure S30. ^{1}H NMR spectrum (600 MHz) of compound **1** in DMSO-*d*$_6$. Zoomed in region between 7.2-13.2 ppm.

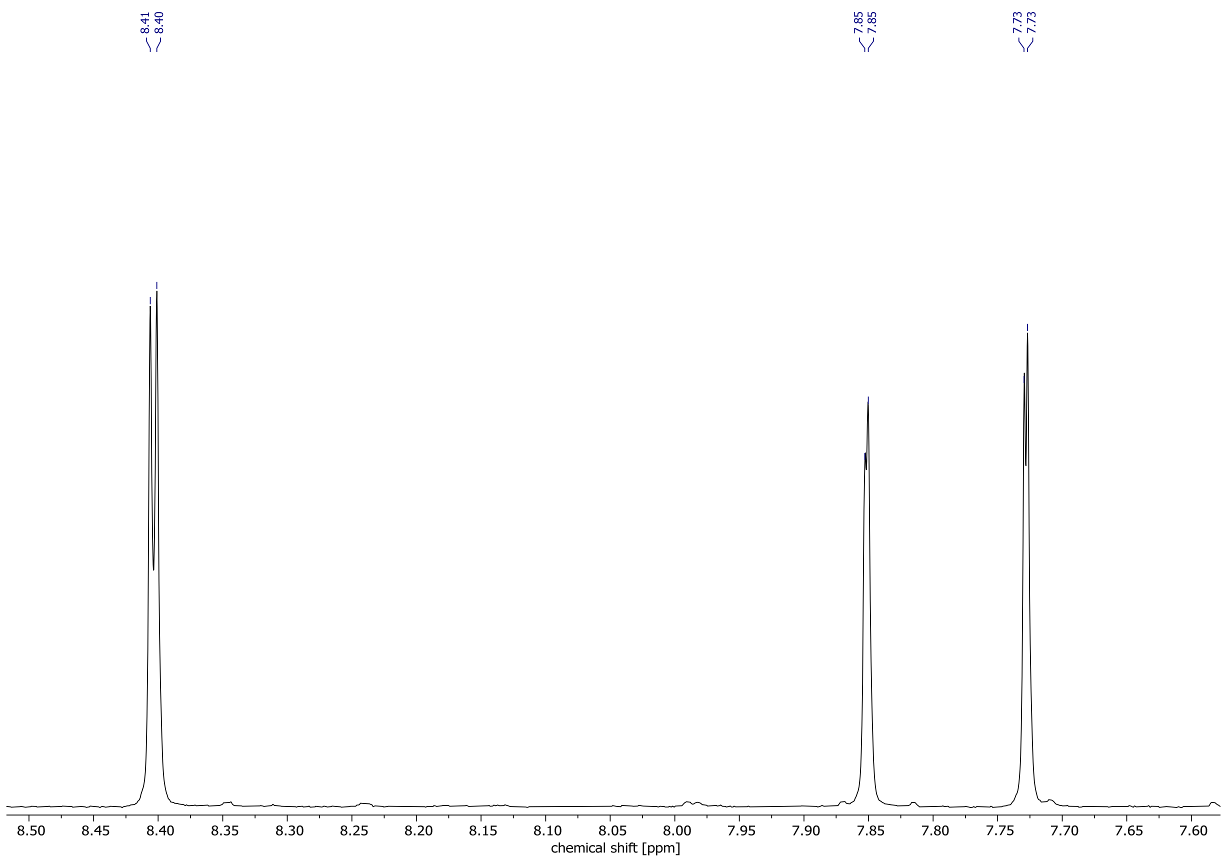

Figure S31. ^{1}H NMR spectrum (600 MHz) of compound **1** in DMSO-d_6. Zoomed in region between 7.6-8.5 ppm.

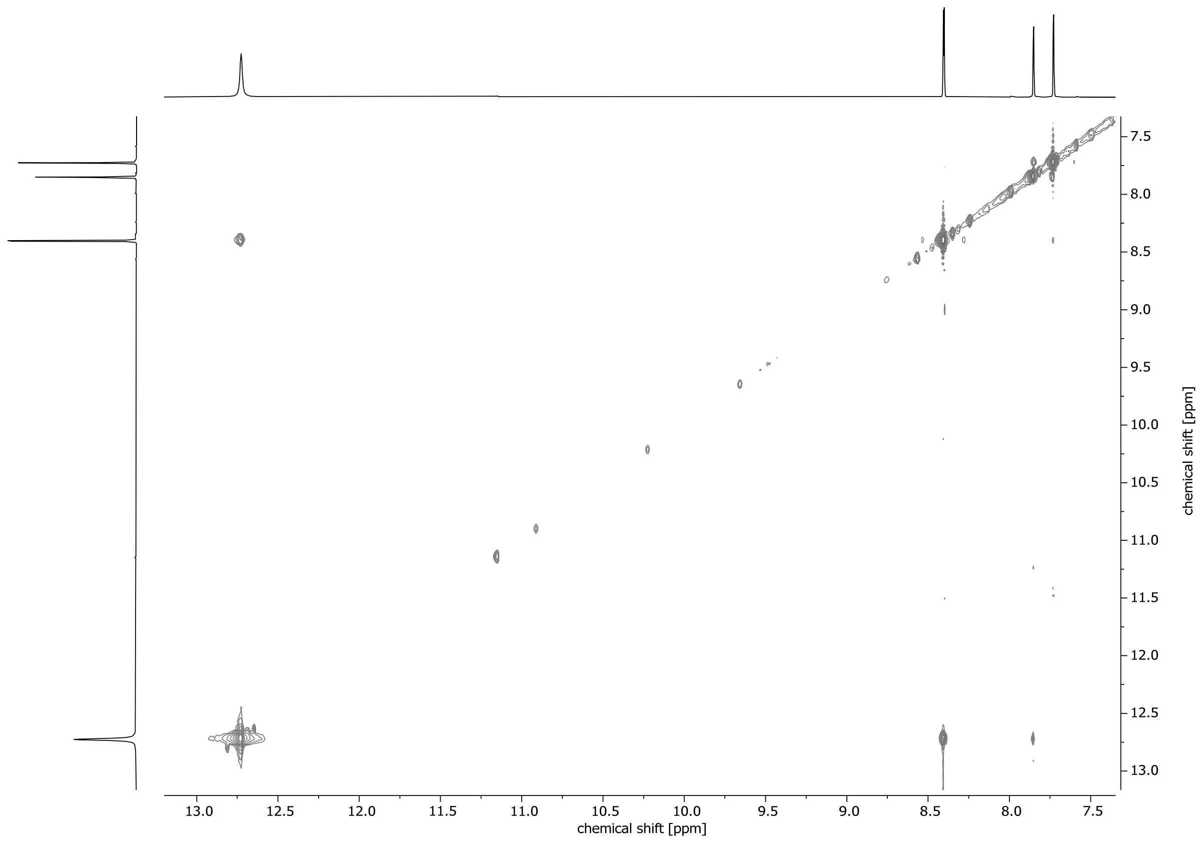

Figure S32. COSY NMR spectrum (600 MHz) of compound **1** in DMSO-d_6. Zoomed in region between 7.5-13.2 ppm.

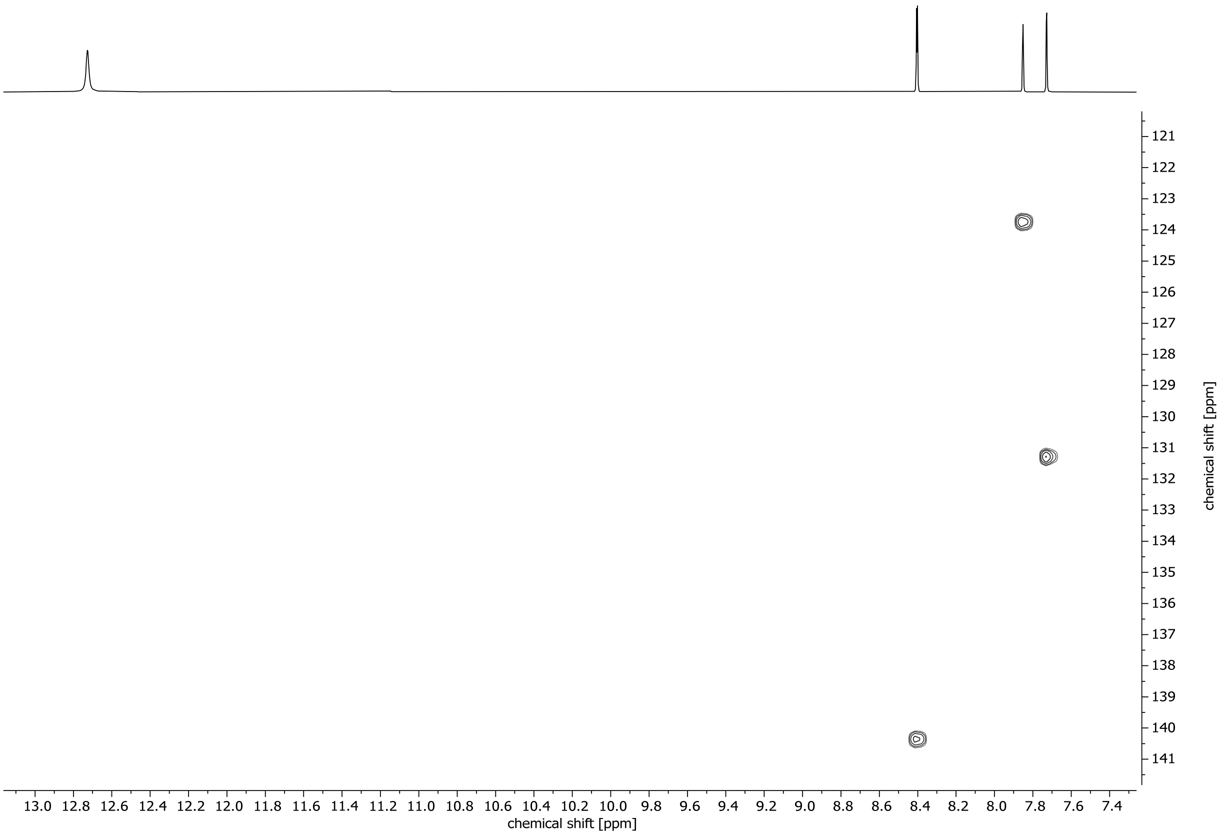

Figure S33. ^{13}C-HMQC NMR spectrum (600 MHz) of compound **1** in DMSO-*d*$_6$. Zoomed in region between 7.2-13.2 ppm.

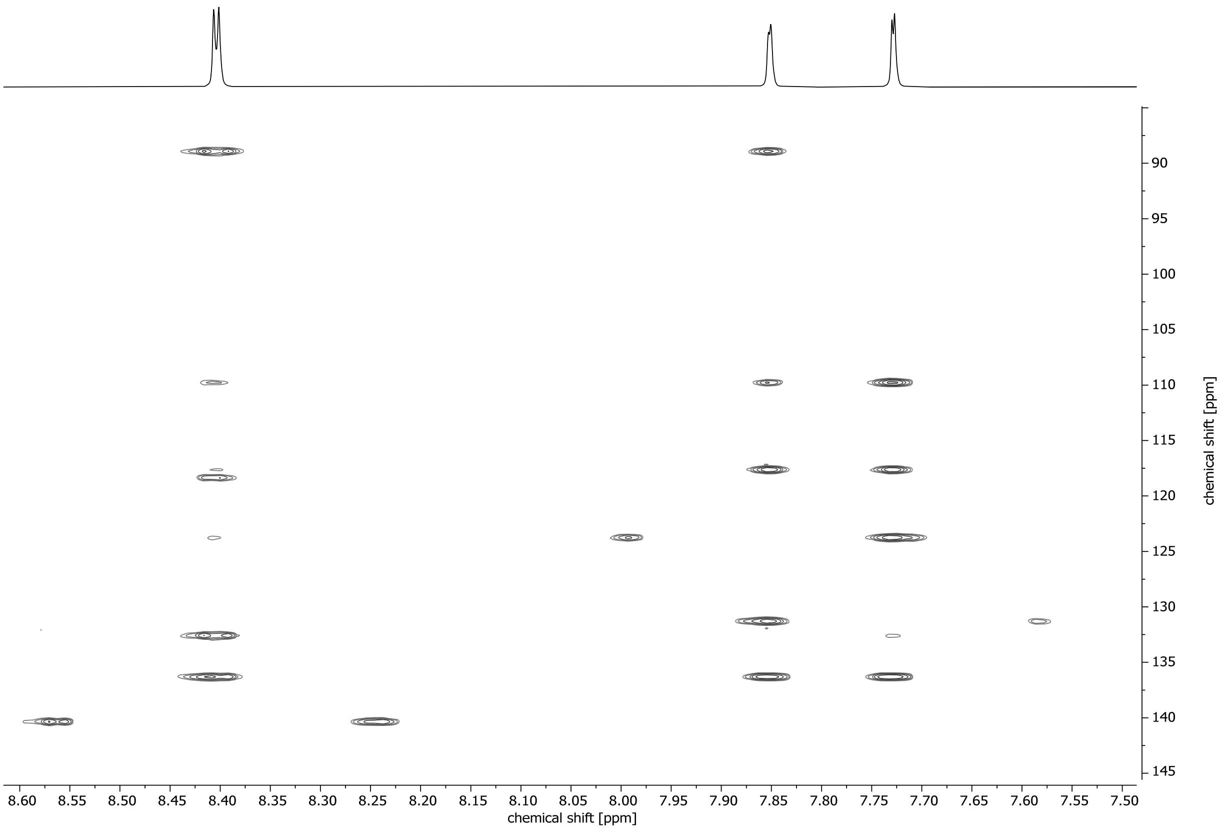

Figure S34. ^{13}C-HMBC NMR spectrum (600 MHz) of compound **1** in DMSO-d_6. Zoomed in region between 7.5-8.6 ppm.

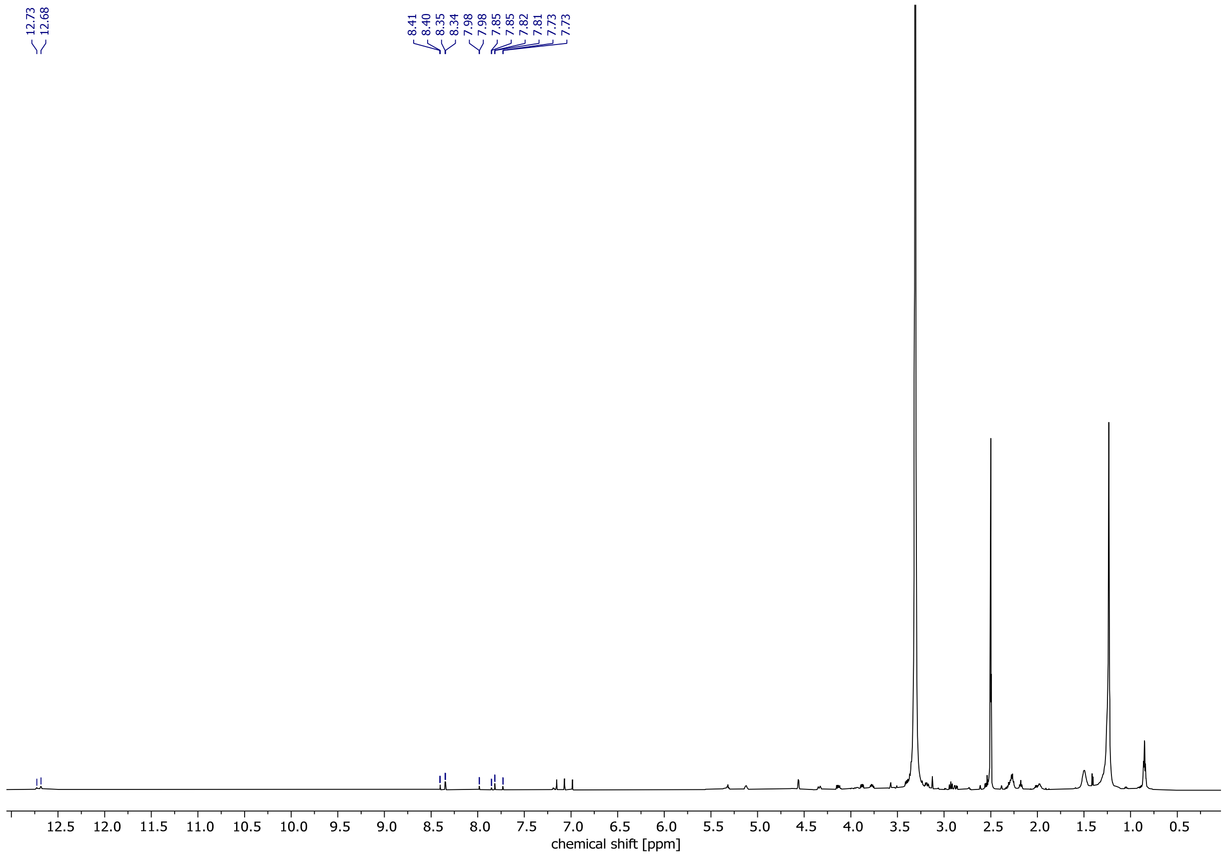

Figure S35. ^{1}H NMR spectrum (600 MHz) of compound **2** mixed with compound **1** in DMSO-d_6. By substracting the signals of compound **1** structure elucidation of compound **2** was achieved.

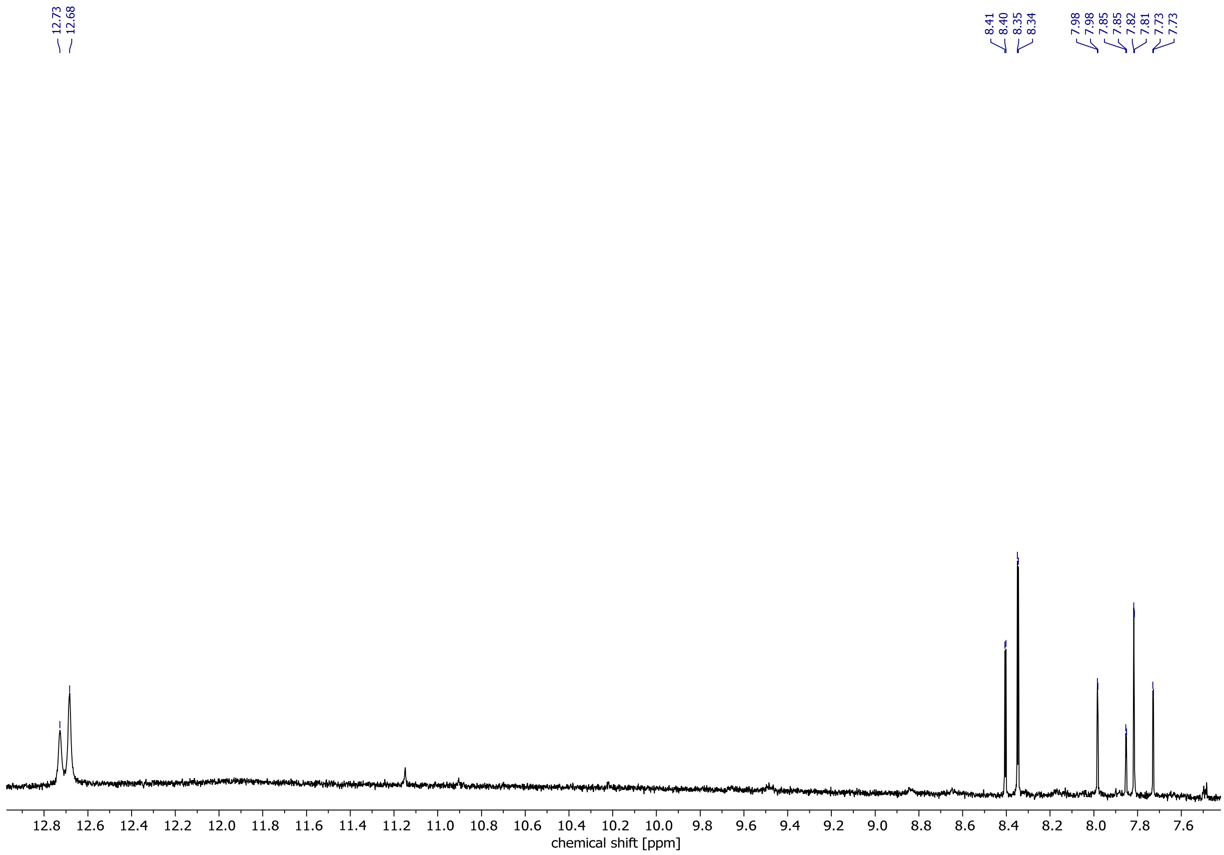

Figure S36. ^{1}H NMR spectrum (600 MHz) of compound **2** in DMSO-d_6. Zoomed in region between 7.5-13 ppm.

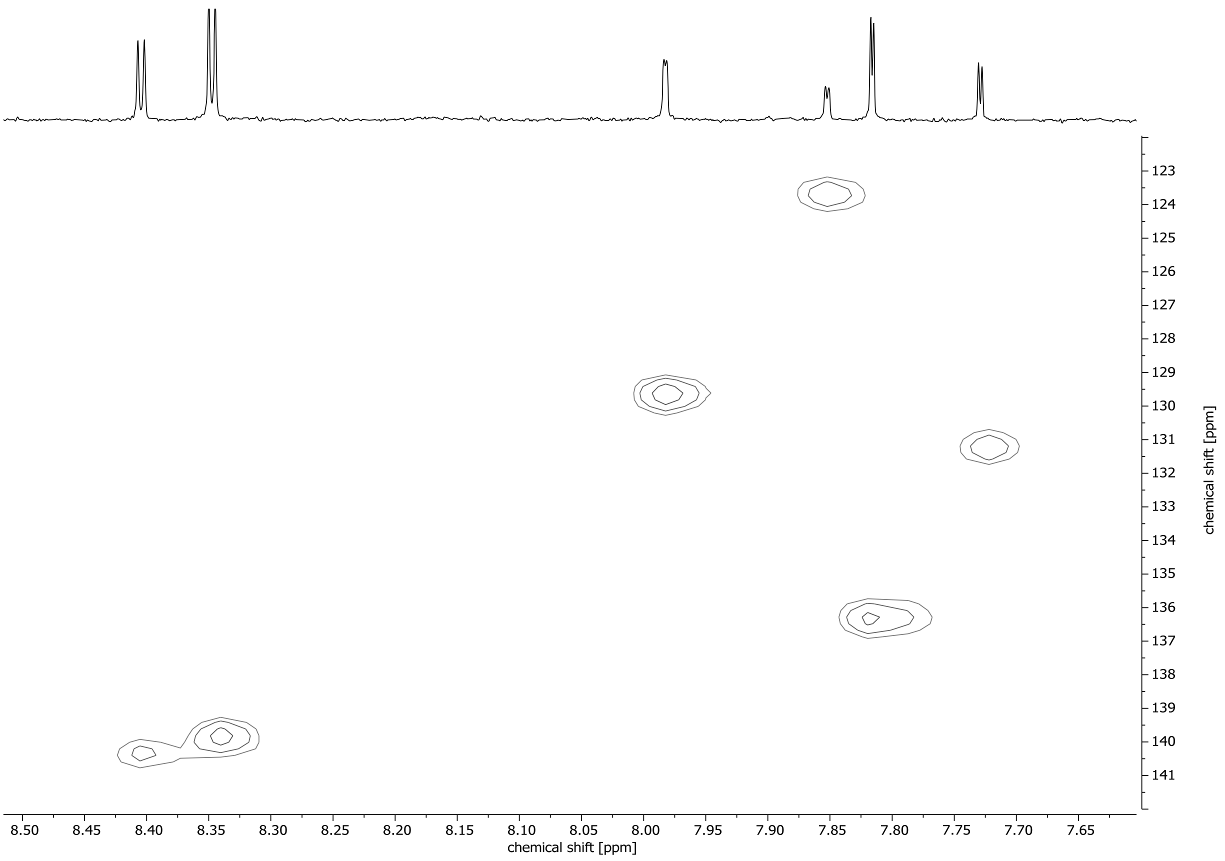

Figure S37. ^{13}C-HMQC NMR spectrum (600 MHz) of compound **2** in DMSO-d_6. Zoomed in region between 7.6-8.5 ppm.

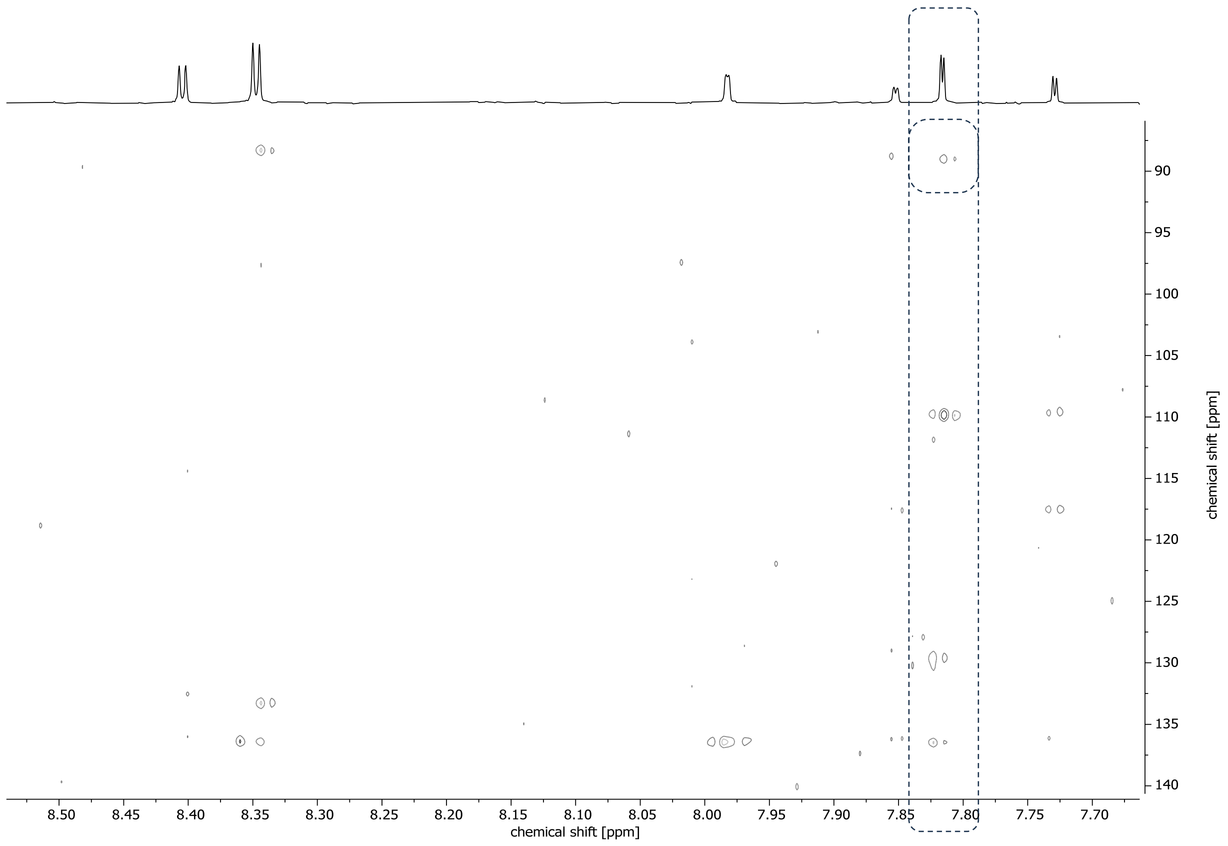

Figure S38. ^{13}C-HMBC NMR spectrum (600 MHz) of compound **2** in DMSO-*d*$_6$. Zoomed in region between 7.6-8.5 ppm. The signals that determine the position of iodination are highlighted.

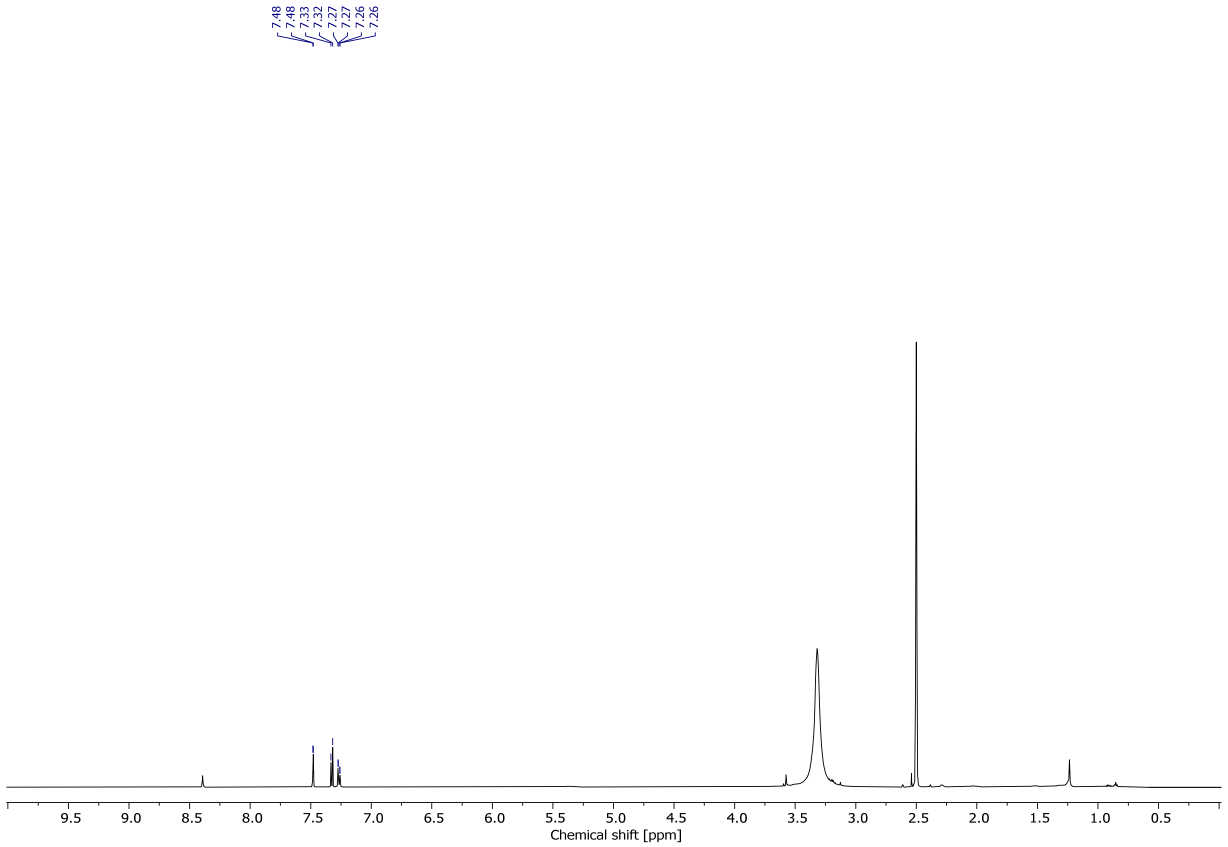

Figure S39. ^{1}H NMR spectrum (600 MHz) of compound **3** in DMSO-d_6.

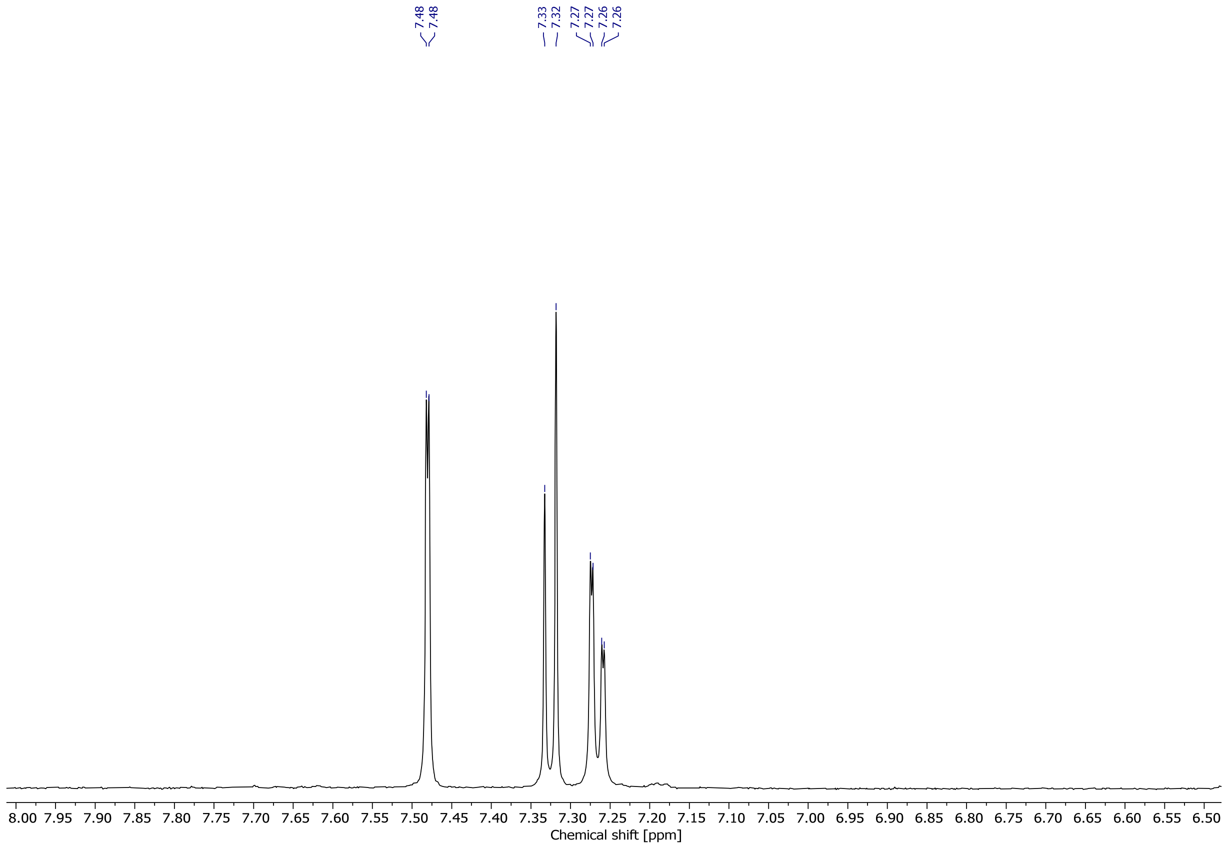

Figure S40. ^{1}H NMR spectrum (600 MHz) of compound **3** in DMSO-*d₆*. Zoomed in region between 6.5-8.0 ppm.

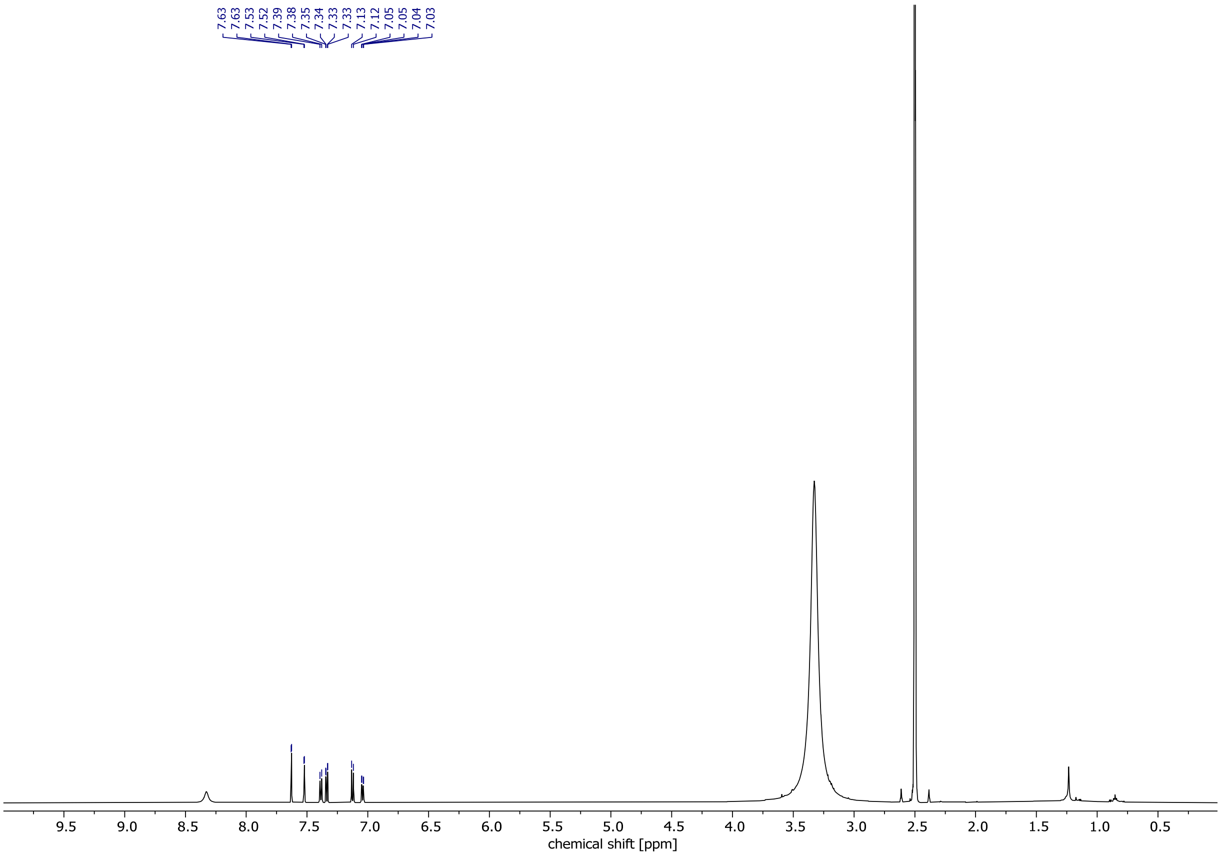

Figure S41. ^{1}H NMR spectrum (600 MHz) of compound **4** in DMSO-d_6.

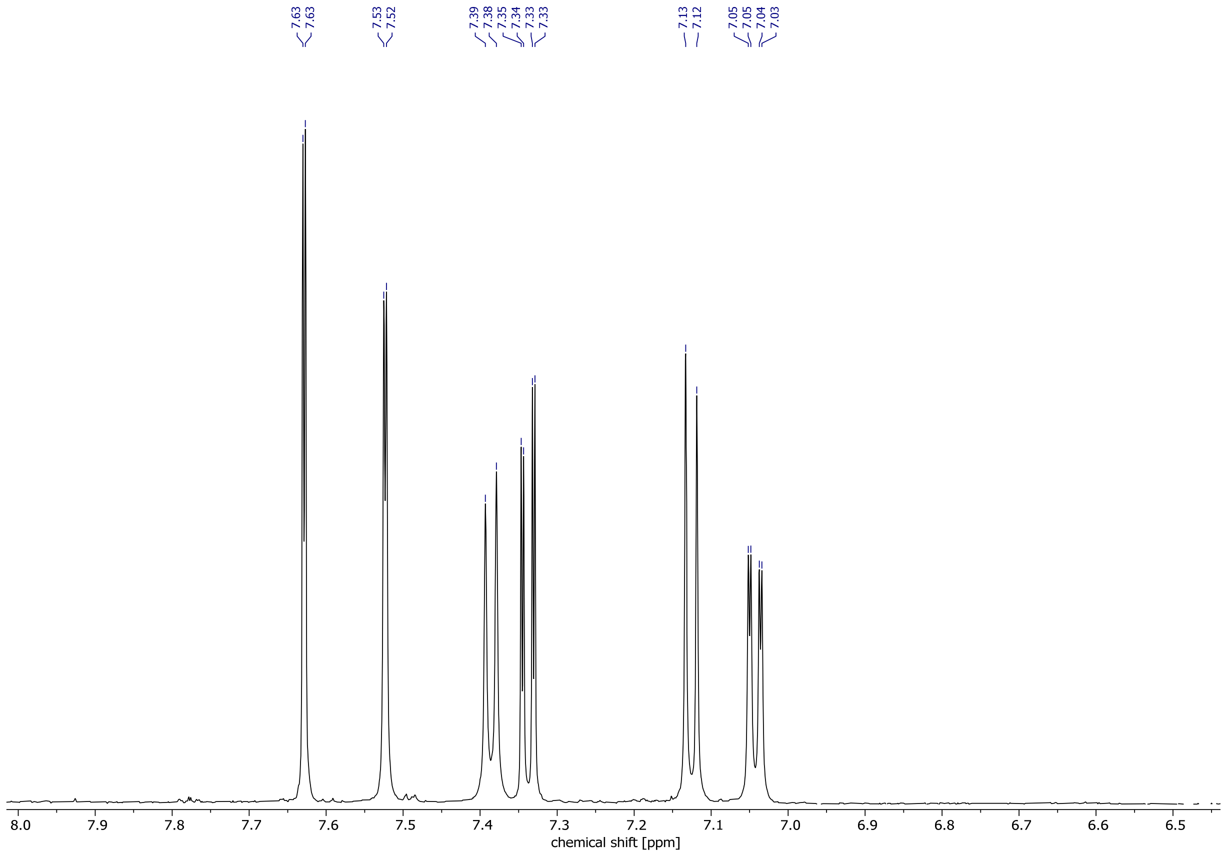

Figure S42. ^{1}H NMR spectrum (600 MHz) of compound **4** in DMSO-d6. Zoomed in region between 6.5-8.0 ppm.

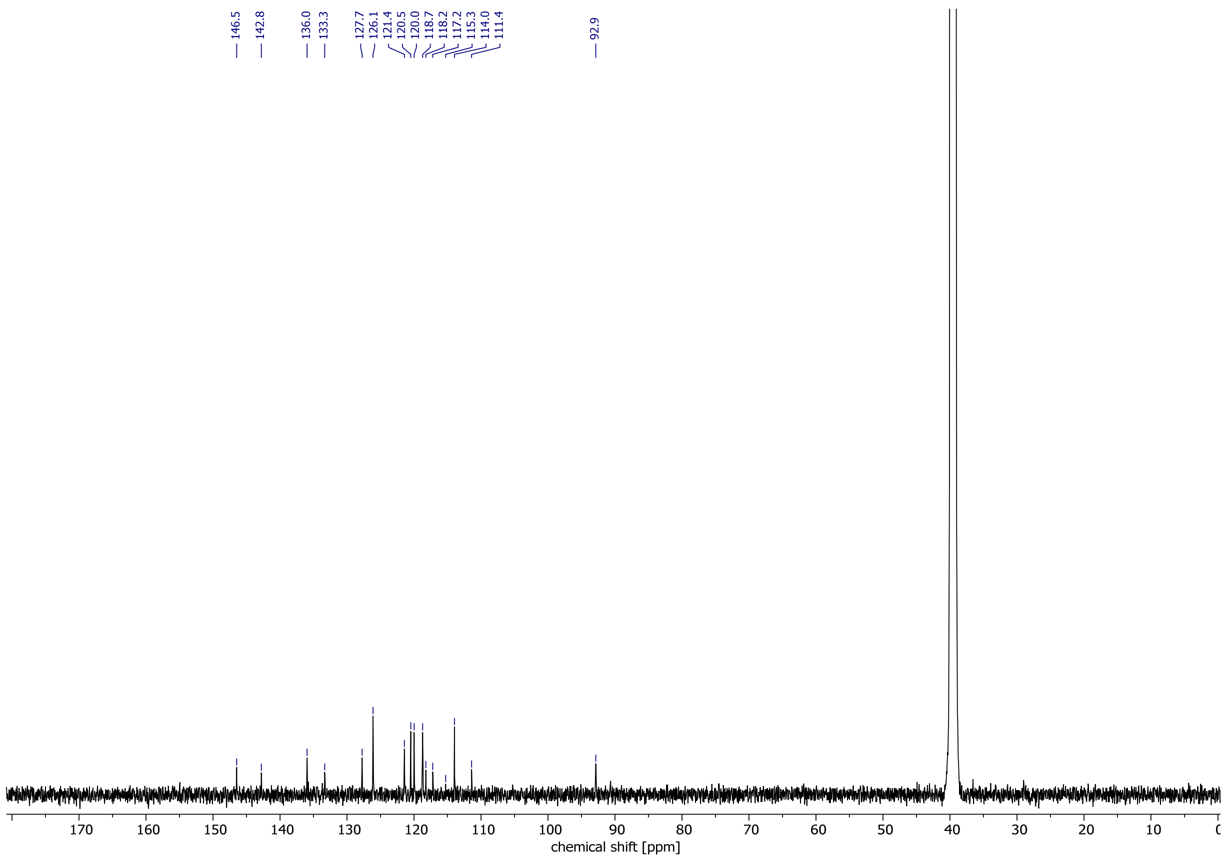

Figure S43. ^{13}C NMR spectrum (150 MHz) of compound **4** in DMSO-d_6. Whole spectral range.

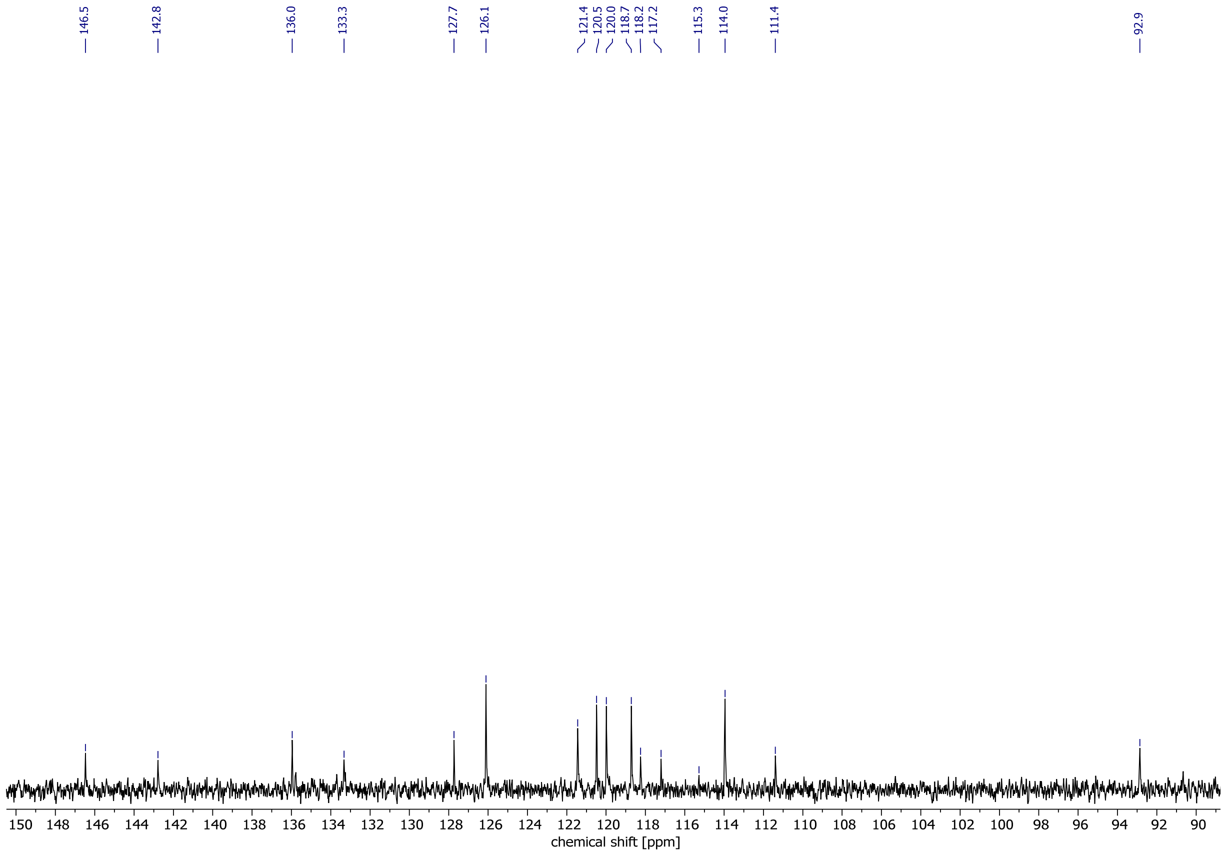

Figure S44. ^{13}C NMR spectrum (150 MHz) of compound **4** in DMSO-*d₆*. Zoomed in region between 90-150 ppm

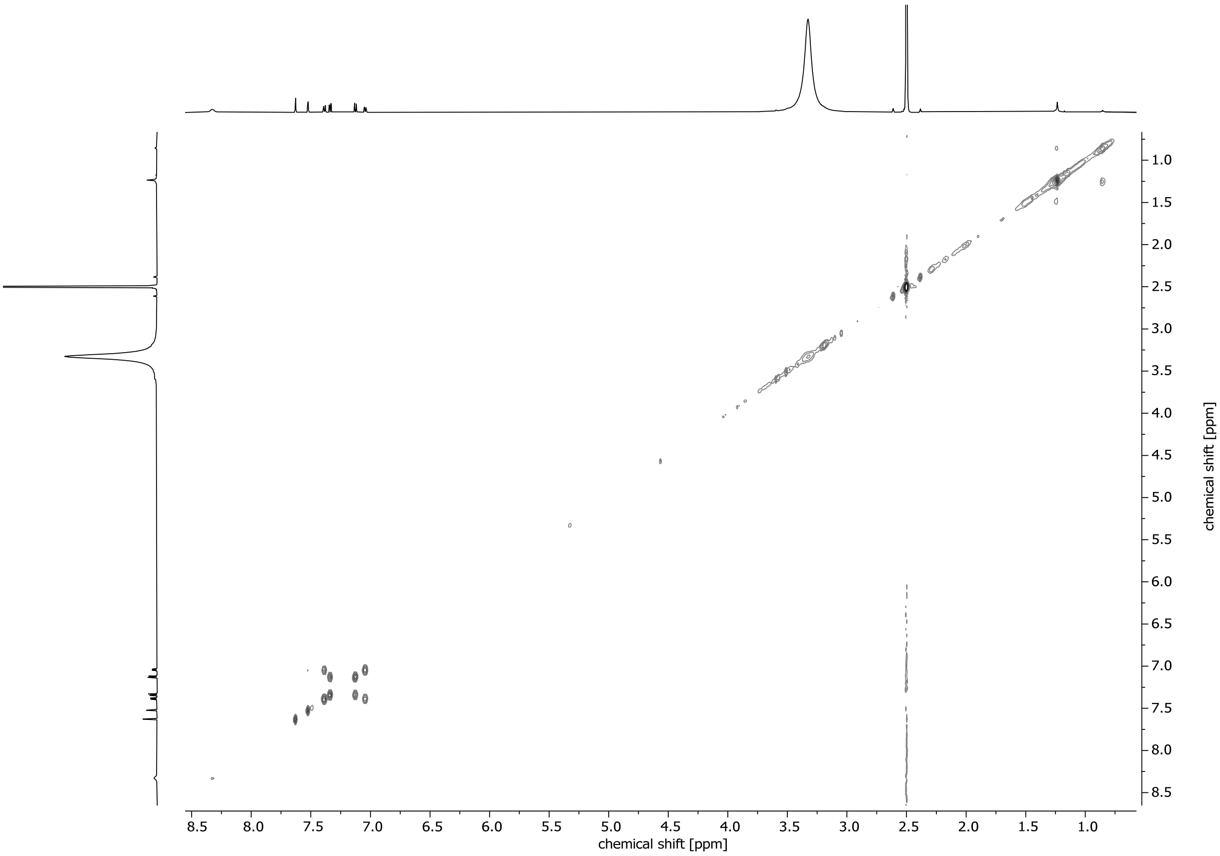

Figure S45. COSY NMR spectrum (600 MHz) of compound **4** in DMSO-d_6.

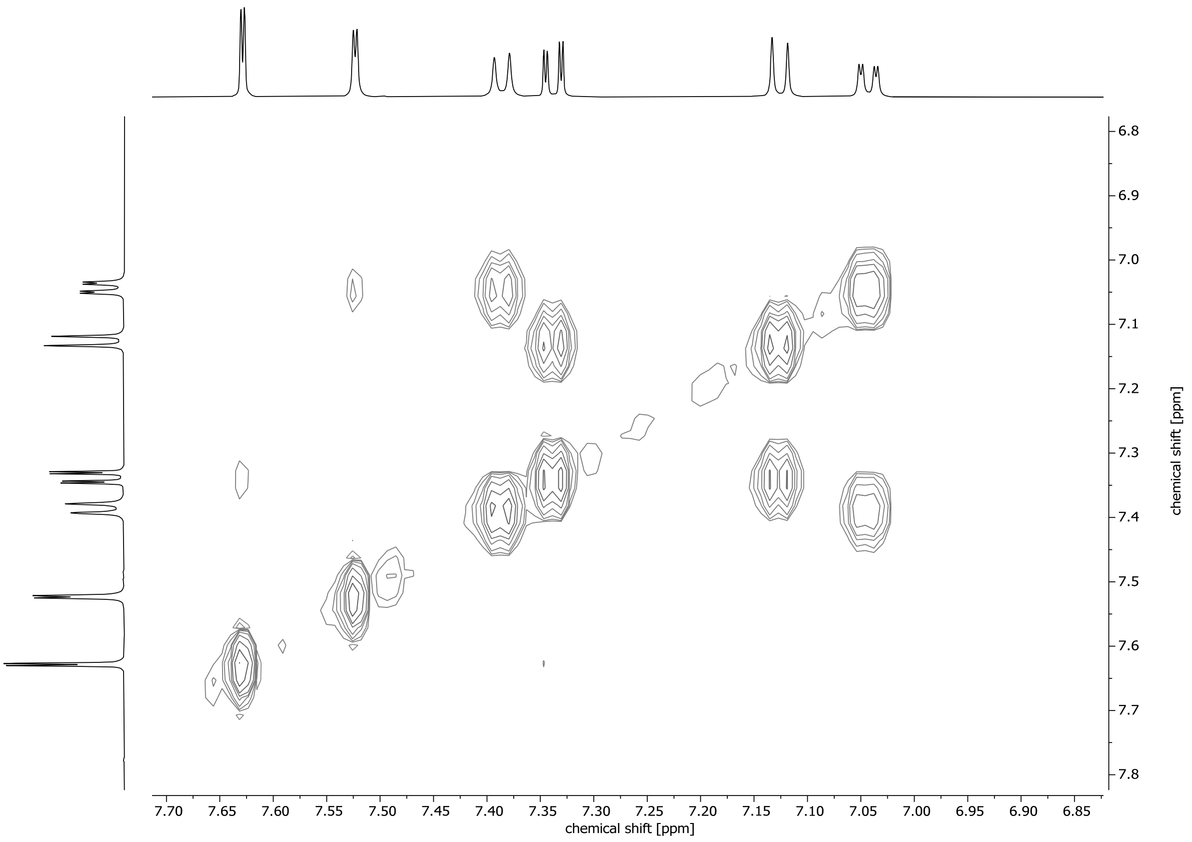

Figure S46. COSY NMR spectrum (600 MHz) of compound **4** in DMSO-d_6. Zoomed in region between 6.8-7.7 ppm.

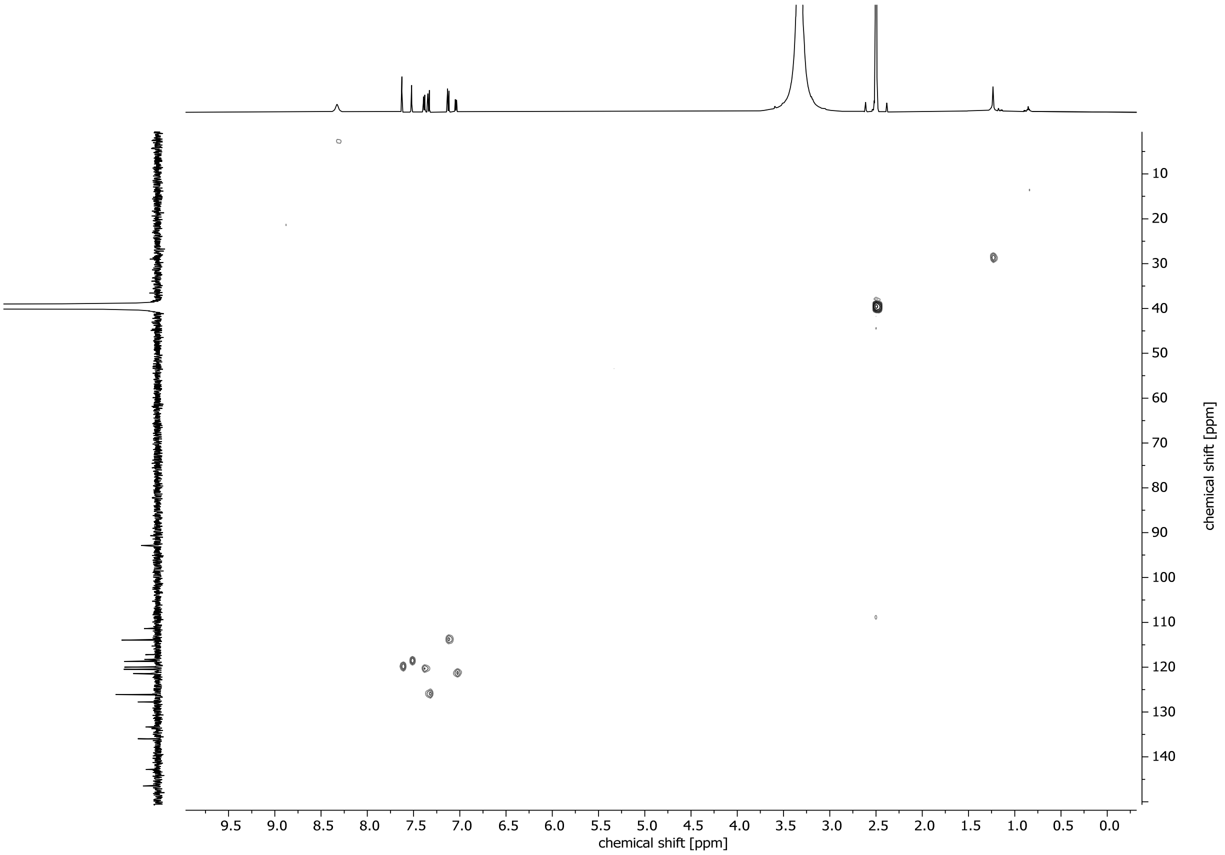

Figure S47. ^{13}C-HMQC NMR spectrum (600 MHz) of compound **4** in DMSO-d_6.

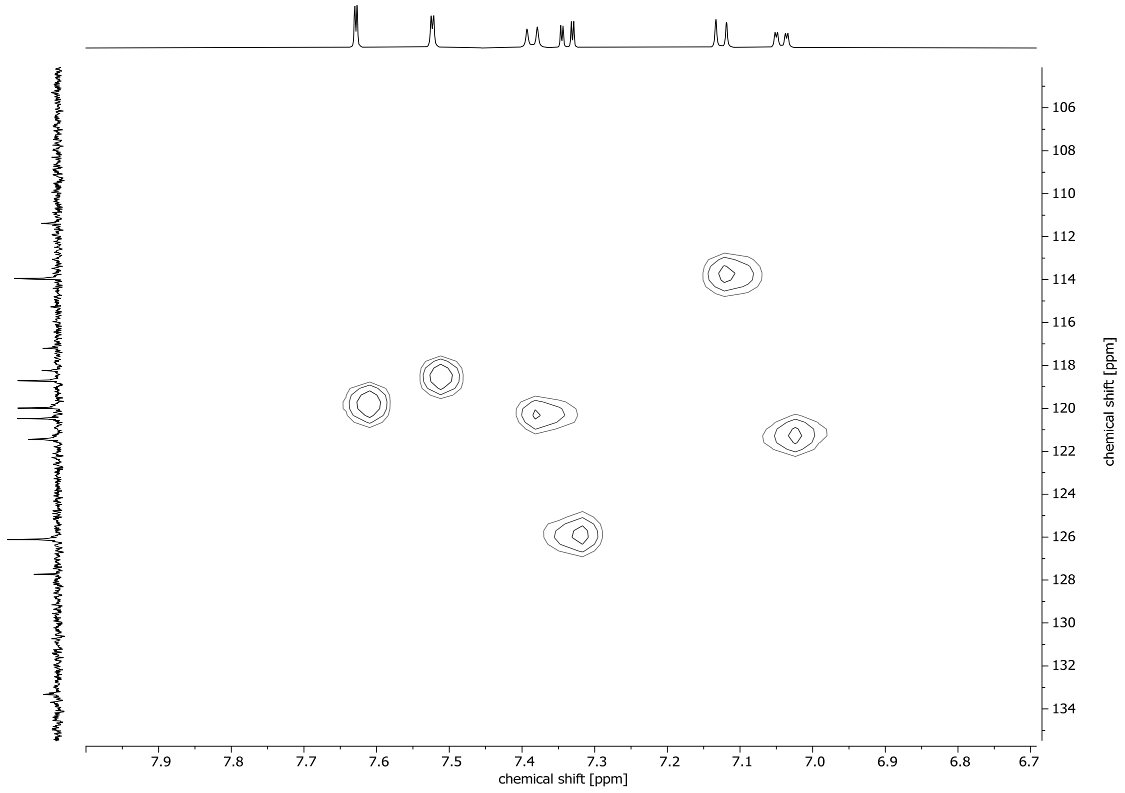

Figure S48. ^{13}C-HMQC NMR spectrum (600 MHz) of compound **4** in DMSO-d_6. Zoomed in region between 6.7-8.0 ppm.

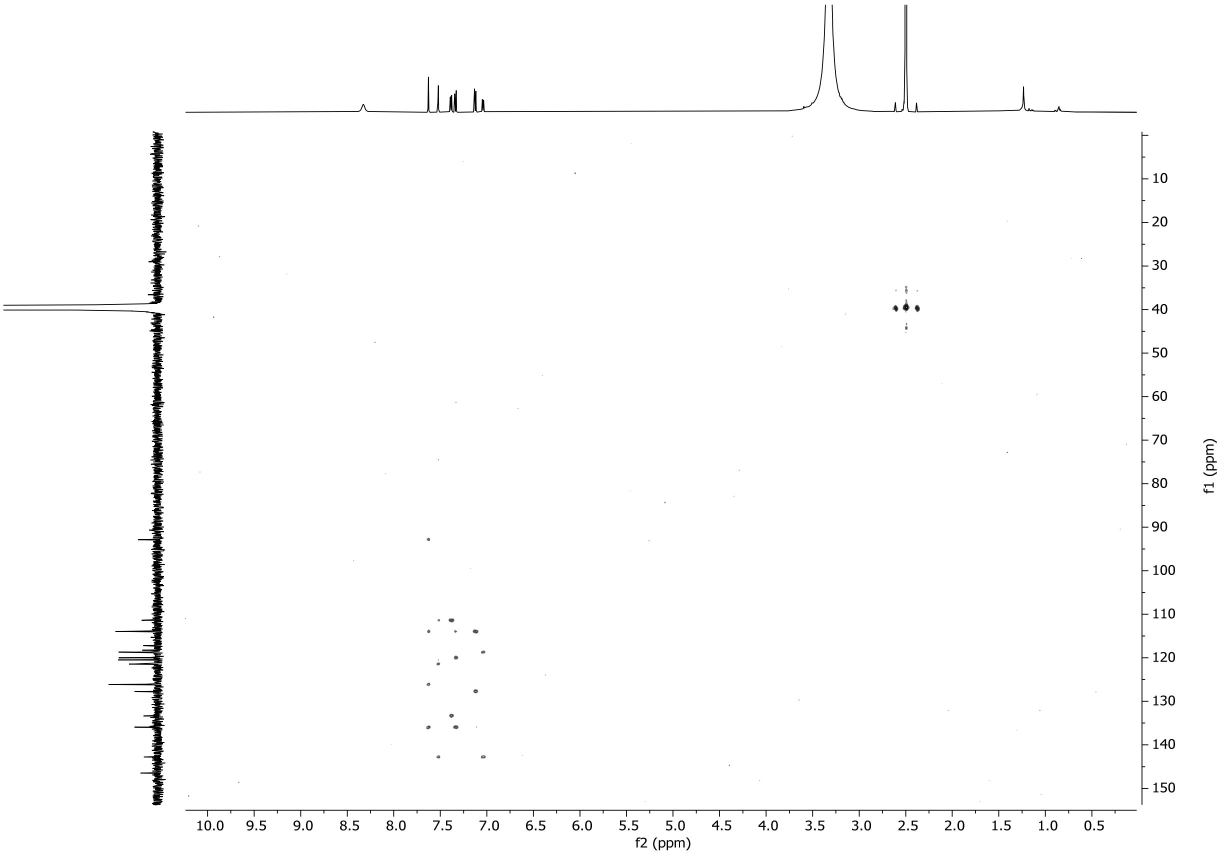

Figure S49. ^{13}C-HMBC NMR spectrum (600 MHz) of compound **4** in DMSO-d_6.

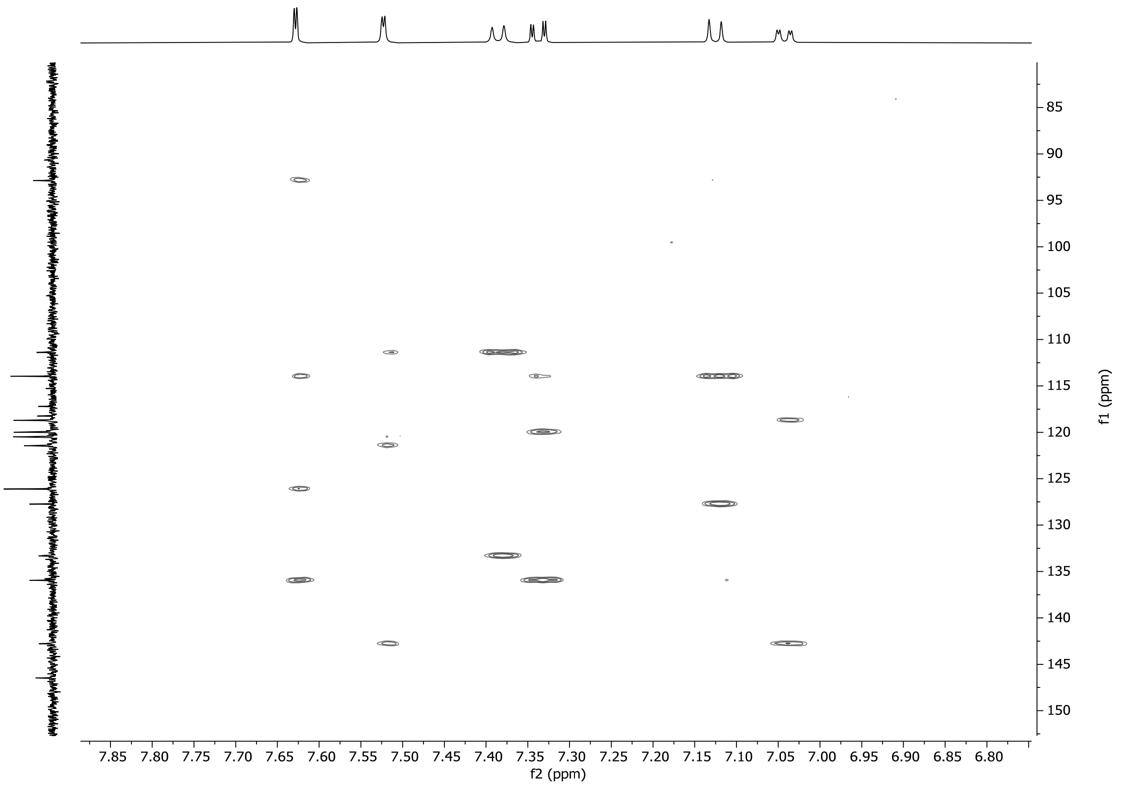

Figure S50. ^{13}C-HMBC NMR spectrum (600 MHz) of compound **4** in DMSO-d_6. Zoomed in region between 6.8-7.8 ppm.

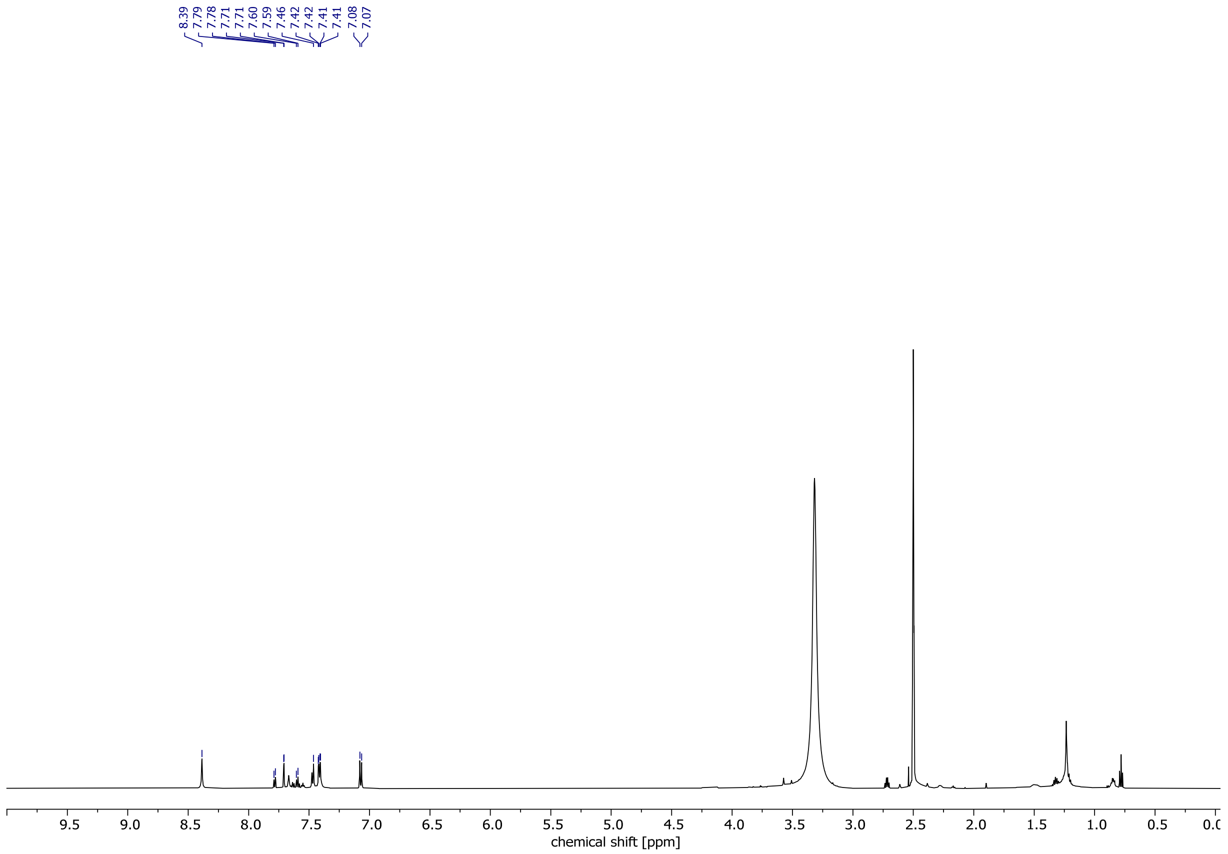

Figure S51. ^{1}H NMR spectrum (600 MHz) of compound **5** in DMSO-d_6.

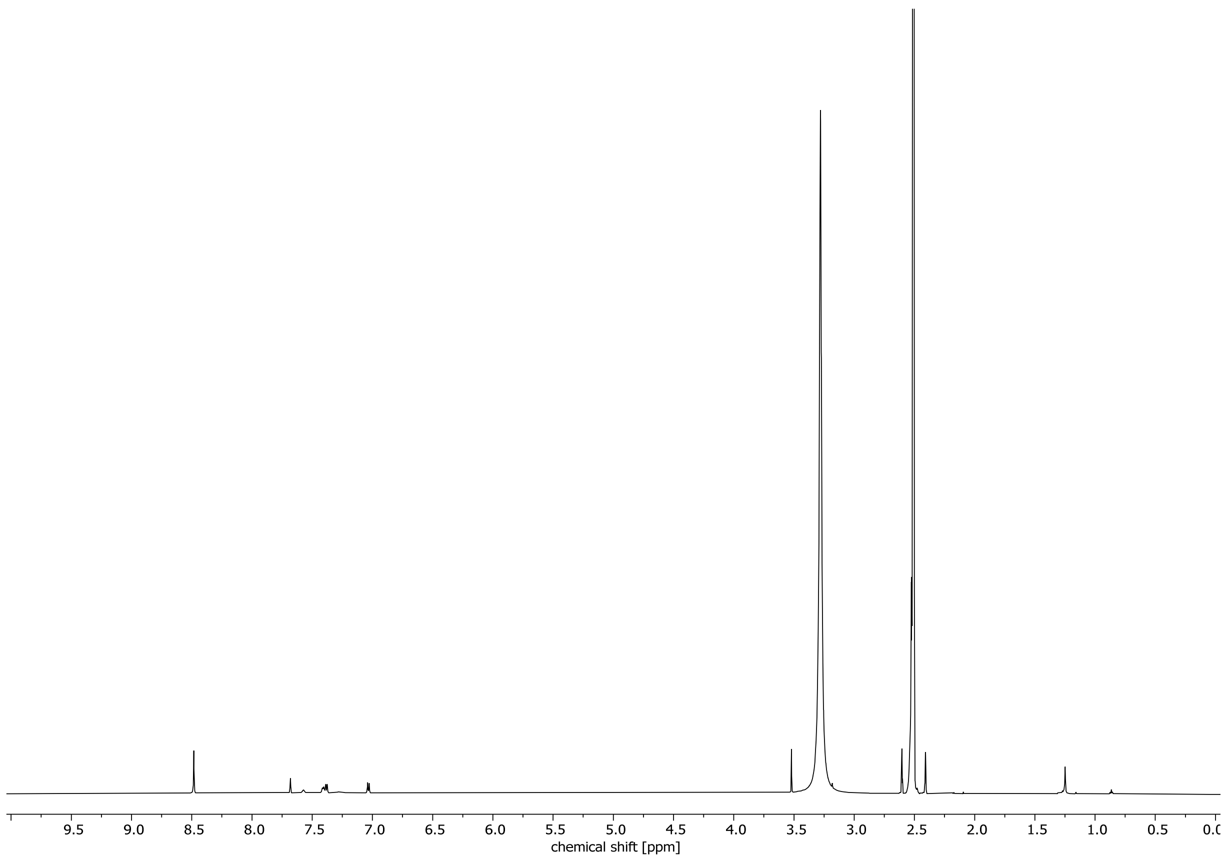

Figure S52. ^{1}H NMR spectrum (600 MHz) of compound **5** in DMSO-d_6, after additional purification.

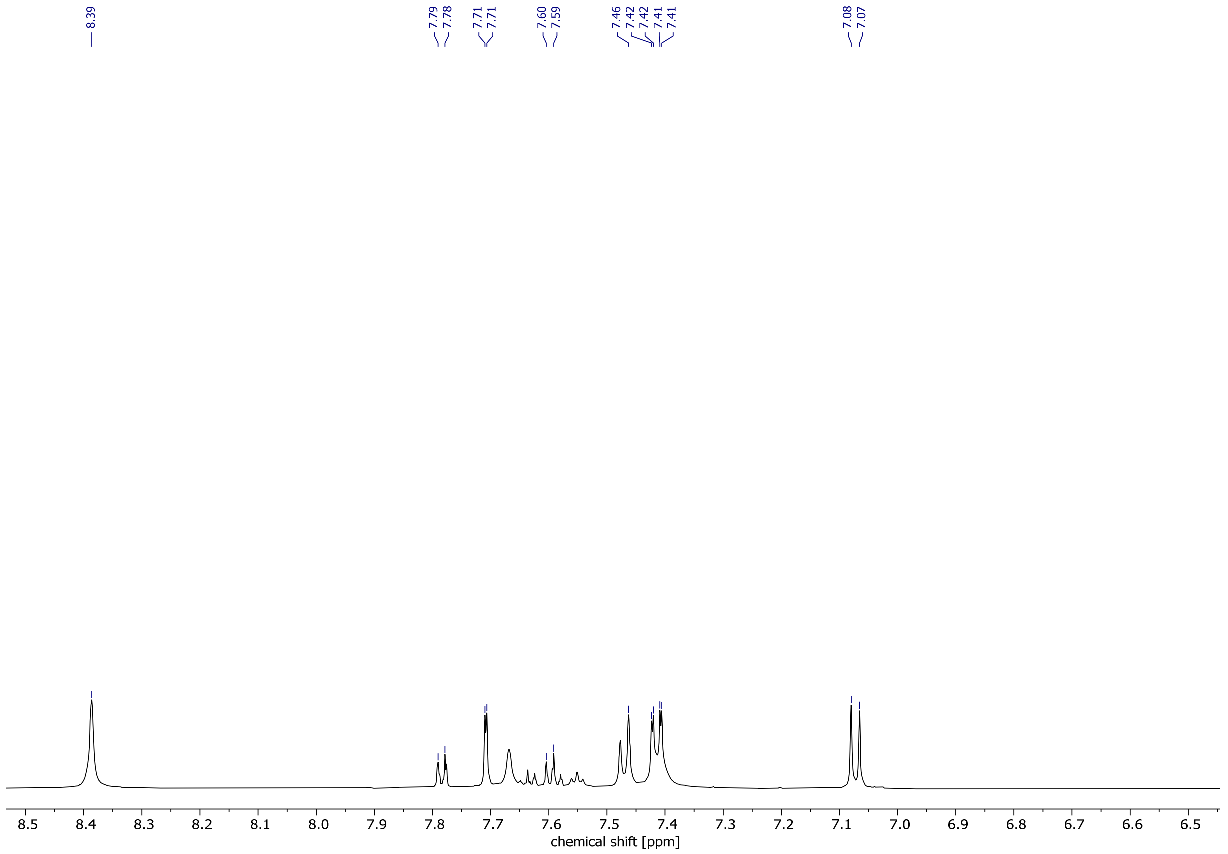

Figure S53. ^{1}H NMR spectrum (600 MHz) of compound **5** in DMSO-d_6. Zoomed in region between 6.5-8.5 ppm.

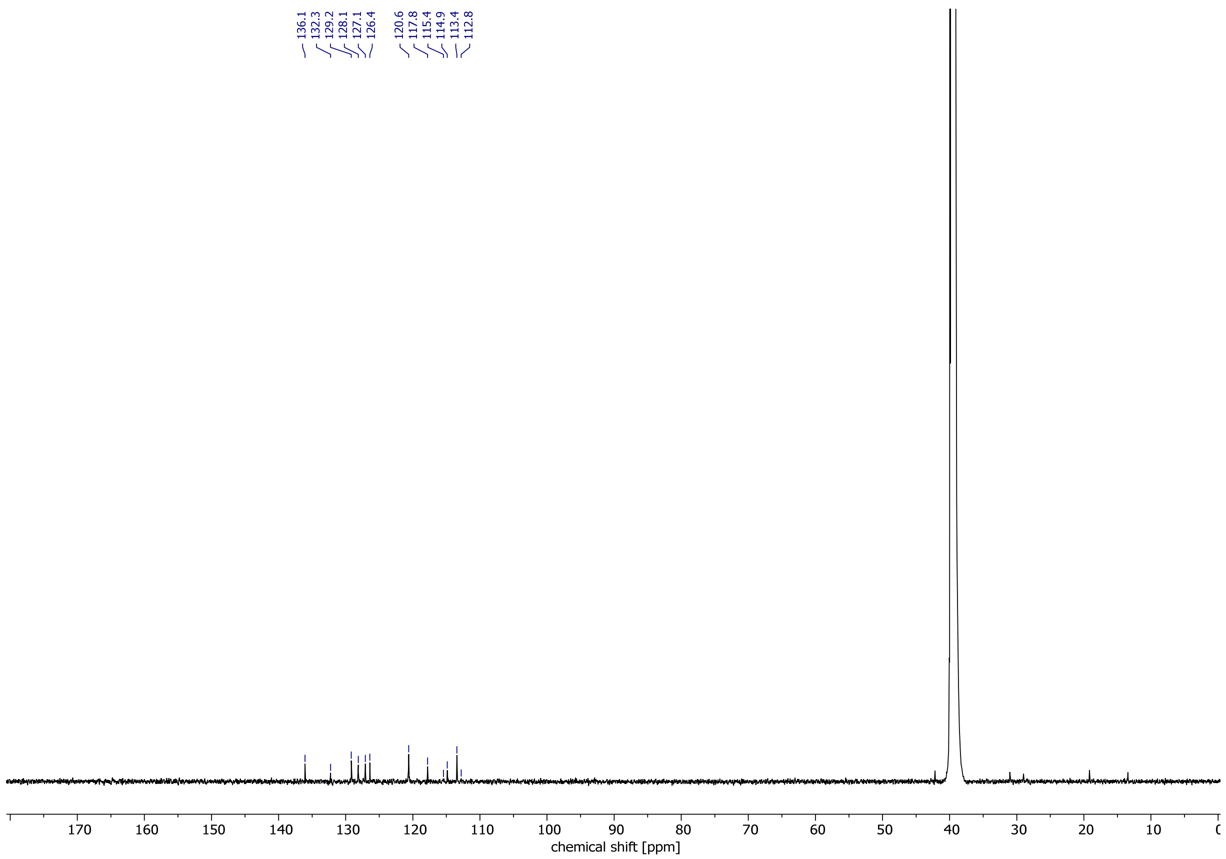

Figure S54. ^{13}C NMR spectrum (150 MHz) of compound **5** in DMSO-d_6. Whole spectral range

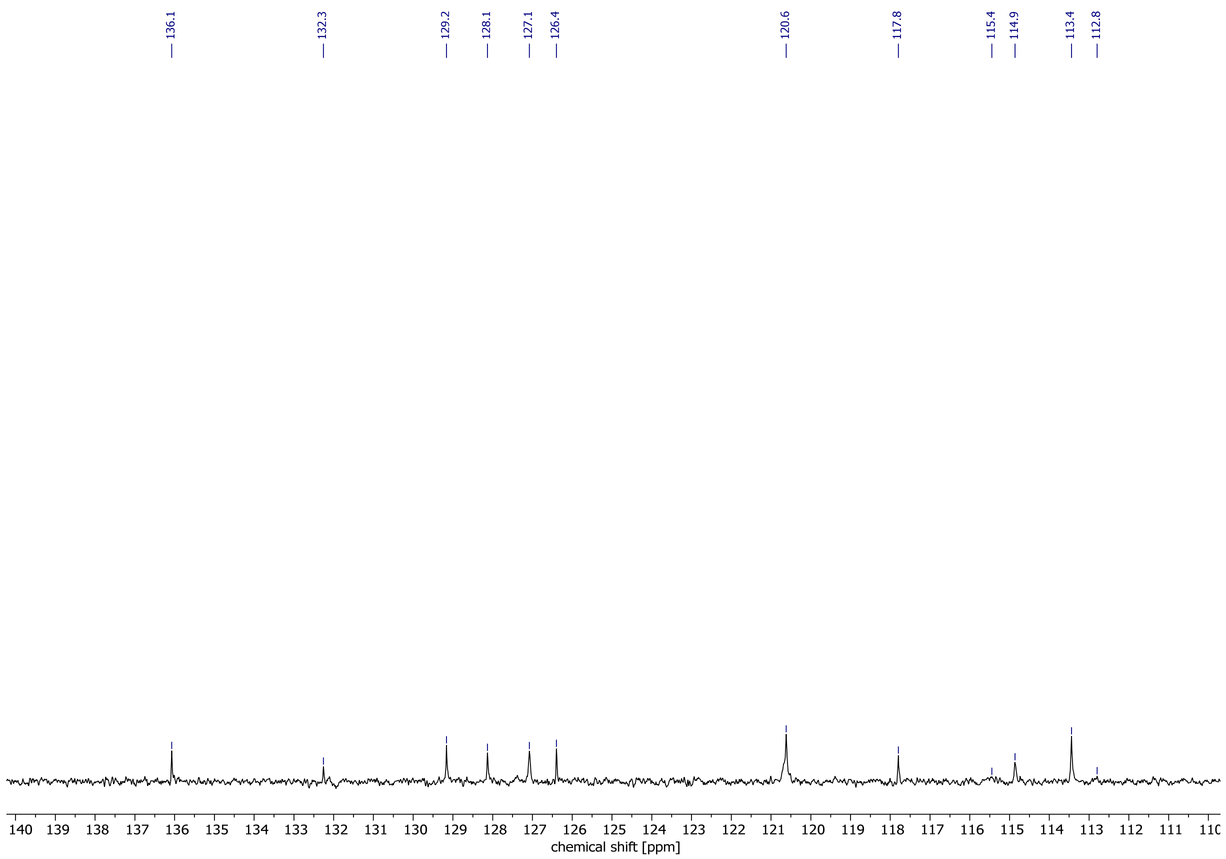

Figure S55. ^{13}C NMR spectrum (150 MHz) of compound **5** in DMSO-d_6. Zoomed in the region between 110-140 ppm.

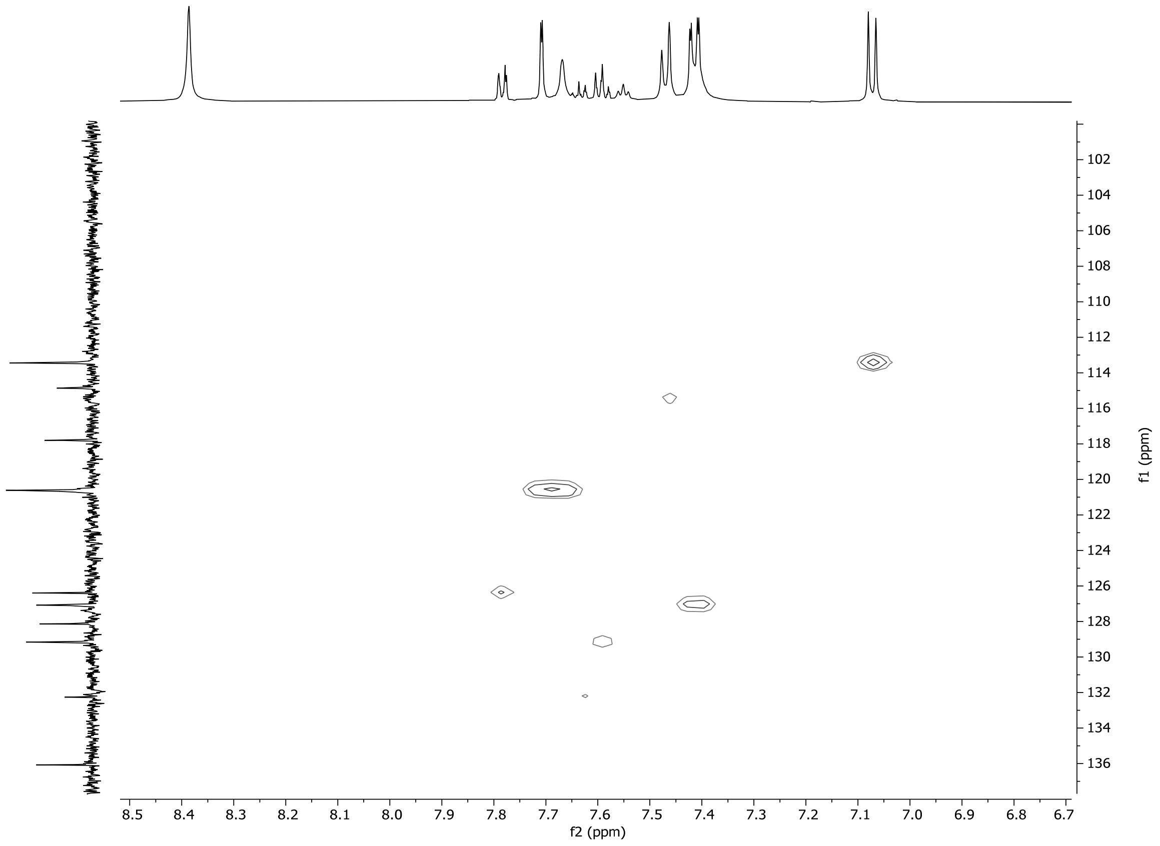

Figure S56. ^{13}C-HMQC NMR spectrum (600 MHz) of compound **5** in DMSO-d_6. Zoomed in region between 6.7-8.5 ppm.

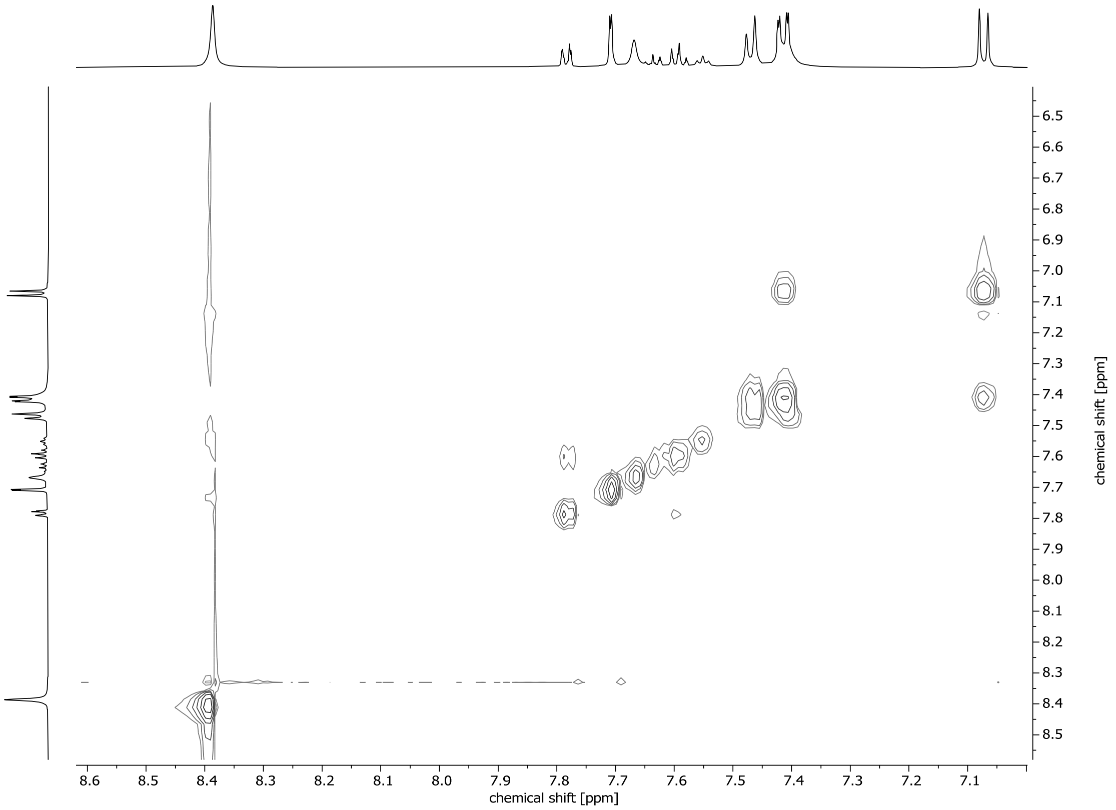

Figure S57. COSY NMR spectrum (600 MHz) of compound **5** in DMSO-d_6. Zoomed in region between 7.0-8.6 ppm.

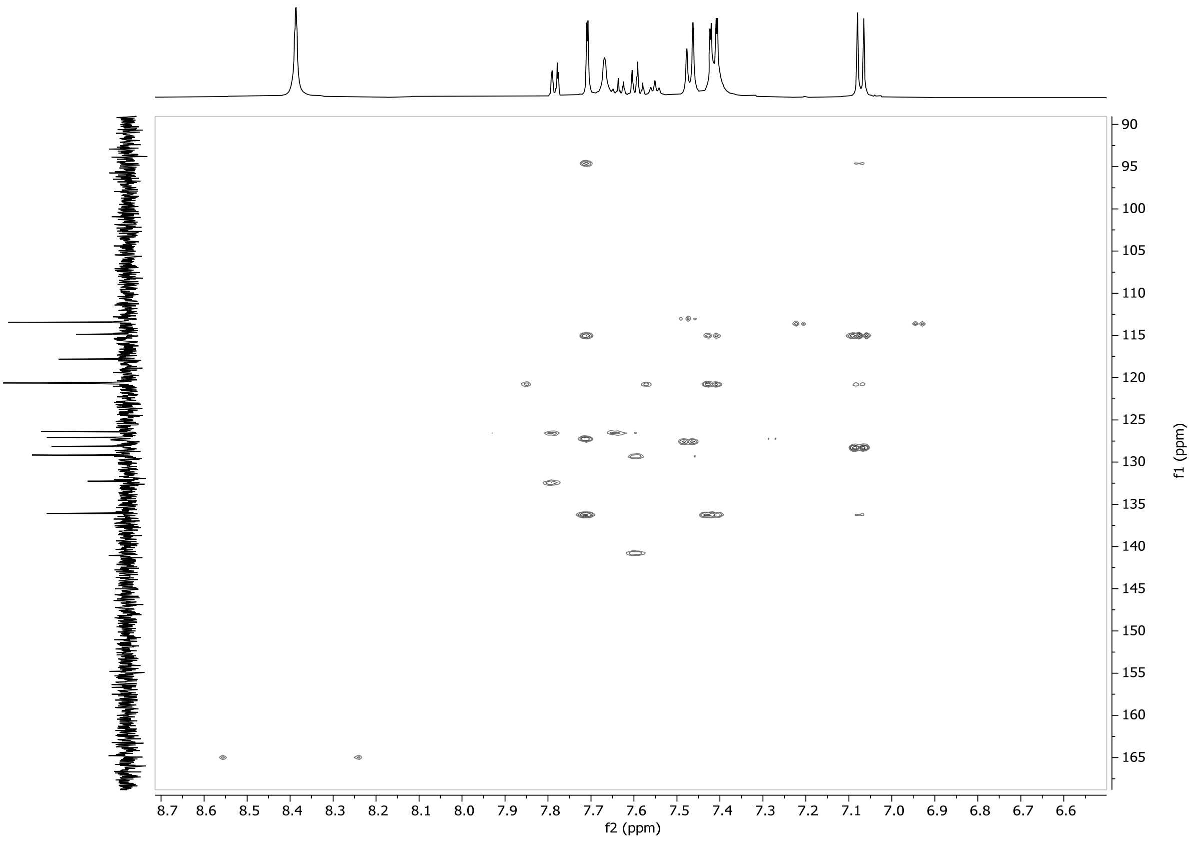

Figure S58. ^{13}C-HMBC NMR spectrum (600 MHz) of compound **5** in DMSO-d_6. Zoomed in region between 6.7-8.7 ppm.
